# A split ribozyme that links detection of a native RNA to orthogonal protein outputs

**DOI:** 10.1038/s41467-023-36073-3

**Published:** 2023-02-01

**Authors:** Lauren Gambill, August Staubus, Kim Wai Mo, Andrea Ameruoso, James Chappell

**Affiliations:** 1 PhD Program in Systems, Synthetic, and Physical Biology, Rice University, Houston, TX 77005 USA; 2 Department of Biosciences, Rice University, Houston, TX 77005 USA; 3 Department of Bioengineering, Rice University, Houston, TX 77005 USA

**Keywords:** Synthetic biology, Synthetic biology, Synthetic biology

## Abstract

Individual RNA remains a challenging signal to synthetically transduce into different types of cellular information. Here, we describe Ribozyme-ENabled Detection of RNA (RENDR), a plug-and-play strategy that uses cellular transcripts to template the assembly of split ribozymes, triggering splicing reactions that generate orthogonal protein outputs. To identify split ribozymes that require templating for splicing, we use laboratory evolution to evaluate the activities of different split variants of the *Tetrahymena thermophila* ribozyme. The best design delivers a 93-fold dynamic range of splicing with RENDR controlling fluorescent protein production in response to an RNA input. We further resolve a thermodynamic model to guide RENDR design, show how input signals can be transduced into diverse outputs, demonstrate portability across different bacteria, and use RENDR to detect antibiotic-resistant bacteria. This work shows how transcriptional signals can be monitored in situ and converted into different types of biochemical information using RNA synthetic biology.

## Abstract (web summary)

RNA provides a unique readout of a cell’s identity, physiologic status, and phenotype. Here the authors deliver an RNA sensing system that can use the information contained within cellular RNA to selectively control the activity of genetic programs.

## Introduction

Cells respond to environmental and intracellular signals by modulating the levels of a wide variety of biomolecules that ultimately determine cell physiology and phenotype. Regulatory mechanisms that operate at the transcriptional level represent a major control point in the process of converting instructions contained in the genome into proteins^1^. RNA thus provides a unique readout of a cell’s identity, physiologic status, and phenotype. The development of genetically-encoded technologies that are able to monitor and transduce individual RNA signals into orthogonal genetic outputs would advance cellular programming such that engineered components can be dynamically coupled with cell state to implement engineered functions^2,3^. Such technologies could, for example, realize genetic programs that are only active in response to species- and cell-specific signatures, link cell states to biochemical processes, and form the foundation for disease-state-sensing therapeutics. Likewise, transducing RNA signals into biomolecular reporters would allow for monitoring of RNA inside living cells to uncover quantitative temporal and spatial patterns of gene expression, and allow us to elucidate a deeper understanding of regulatory networks and the processes they control. Compared to monitoring proteins, RNA detection is an attractive option as it can be implemented using easily-designed Watson-Crick base pair interactions, operates on fast timescales^4^, expands the breadth of detection to include non-coding RNA^5^, and can theoretically access both geno- and phenotypic information of host cells. Thus, there is a strong motivation to create plug-and-play RNA detection platforms able to transduce intracellular RNA signatures into diverse, orthogonal biomolecular outputs for cell monitoring and genetic programming applications.

In protein engineering, technologies that use protein-protein interactions to control the functionality of an orthogonal protein output have been achieved using topological engineering of split proteins^6,7^. These systems are based on a protein output that is split into two inactive fragments, where interacting protein domains are translationally fused to each fragment. Upon interaction of the fused protein domains, the two fragments of the split-protein output are co-localized and the function of the protein is restored. As a result of the versatility of this approach, a wide range of protein outputs, from enzymes to transcription factors, have been engineered as split proteins^8–14^. Similarly, a variety of protein interaction domains have been used to sense and respond to specific chemical and cellular interactions, yielding an abundance of tools to detect protein-protein interactions^15^, sense small molecules^16^, and implement synthetic gene networks^14^.

When it comes to RNA, programming synthetic RNA to interact with a given RNA signature through Watson-Crick base pairing is relatively straightforward, but transducing this interaction into a biomolecular output in a controlled and orthogonal manner remains challenging. Most RNA sensors have leveraged strand displacement mechanisms that use an RNA input to displace an inhibitory RNA structure, to allow for activation of an output^17,18^. While the potential of using split-RNA reporters and split ribozymes for RNA sensing applications has been previously recognized^19–21^, these technologies typically suffer from limitations that include low dynamic range (on/off signal), a lack of quantitative design rules to guide the design of new detection interactions, and a lack of modularity for different classes of biomolecular outputs. To address these drawbacks, we have engineered a high-performing, plug-and-play RNA-sensing platform, which we call Ribozyme-ENabled Detection of RNA (RENDR). With RENDR, a cellular RNA input activates a splicing reaction that, in turn, produces an mRNA encoding any chosen orthogonal protein output. To achieve this regime, a splicing ribozyme is synthetically split into two non-functional fragments that are each appended with RNA guide sequences that are designed to interact with the RNA input. The split ribozyme is then inserted within the desired gene output. When present, the RNA input co-localizes the two transcribed ribozyme fragments, forming a functional ribozyme complex, and the ribozyme splices together the mRNA of the protein output. To create the RENDR platform, we first characterized nearly all potential split sites across the splicing ribozyme from *Tetrahymena thermophila* by applying for the first time a high-throughput laboratory evolution approach from protein engineering to engineer split RNA. Using this strategy, we profiled functional split sites across the ribozyme structure and identified split designs that allowed for high dynamic range of detection of up to 93-fold. We then investigated the design rules for RNA detection by systematically modifying the interactions between the RNA input and RENDR. By characterizing these variants, we derived a simple thermodynamic model that can guide the design of RENDR variants to transduce new RNA signals. To allow for the facile exchange of different genes encoding protein outputs, we explored a modular design strategy that we then harnessed to create RENDR variants that transduce RNA input signals into fluorescent, colorimetric, gaseous, and regulatory outputs. We then demonstrated that RENDR is functionally portable across different bacteria without the need for any genetic modifications. Finally, as a proof of principle application, we demonstrated that RENDR can be used to couple the production of pigment-producing enzymes to the presence of antibiotic resistance genes (ARG), creating a low-cost and facile approach to detecting antibiotic-resistant microbes.

## Results

### High-throughput identification of ribozyme split sites using in vitro transposon mutagenesis coupled with fluorescence-activated cell sorting

**Fig. 1:**
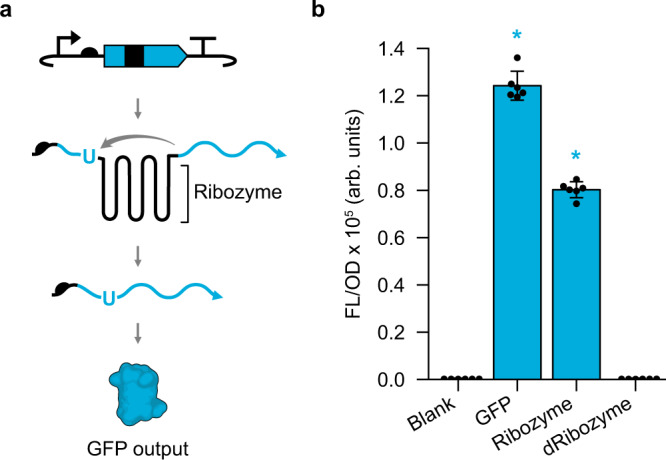
Fluorescence splicing assay enables convenient measurement of ribozyme splicing activity. **a** Splicing assay utilizes a plasmid containing an sfGFP coding sequence (blue) with the *Tetrahymena thermophila* ribozyme DNA sequence (black) inserted within codon Y66. When this DNA is transcribed into RNA, the ribozyme splices together the sfGFP exons at the 5’ splice site (blue U), located just upstream of the ribozyme, and the spliced product is translated into the native sfGFP (GFP output). **b** Fluorescence characterization (measured in units of fluorescence [FL]/optical density [OD] at 600 nm) performed in *E. coli* transformed with an empty plasmid (Blank), a plasmid containing sfGFP (GFP), a plasmid containing sfGFP with an inserted wild-type ribozyme (Ribozyme), and a plasmid containing sfGFP with an inserted catalytically dead G264A mutant ribozyme (dRibozyme). Bars show mean values and error bars represent s.d. of *n* = 6 biological replicates shown as points. To determine significance, a two-tailed t-test (alpha = 0.05) resulted in *p*-values of 2.6e−13, 5.5e−14, and 0.86 for GFP, Ribozyme, and dRibozyme relative to Blank, respectively. Statistically significant differences (*p* < 0.05) are indicated by asterisks above bars. Source data for (**b**) are provided as a Source Data file.

To enable convenient measurement of the ribozyme splicing reaction, we first established a fluorescence-based splicing assay in *Escherichia coli (E. coli)*. To do this, a splicing ribozyme from *Tetrahymena thermophila* was inserted within the coding sequence (CDS) of a super folder green fluorescent protein (*sfGFP*) gene such that translation of the full-length protein is disrupted in the absence of splicing. Upon transcription and splicing, the ribozyme removes itself from the flanking exons and ligates together the exons to form a functional *sfGFP* mRNA, which is then translated to produce a fluorescent protein output (Fig. 1a). To decide where to insert the ribozyme within the *sfGFP* CDS, two design criteria were used: (1) the ribozyme must be inserted immediately downstream of a uracil, which is required for splicing, and (2) the ribozyme should be inserted at a position within *sfGFP* such that translation of the first exon does not produce a fluorescent signal. Based on these criteria, we constructed a series of plasmids using several ribozyme insertion sites and measured the fluorescence produced by each in *E. coli* (Supplementary Fig. 2). From these data, we chose to proceed with the ribozyme inserted after the first nucleotide of amino acid Y66 in the *sfGFP* CDS (Fig. 1b and Supplementary Fig. 3).

**Fig. 2:**
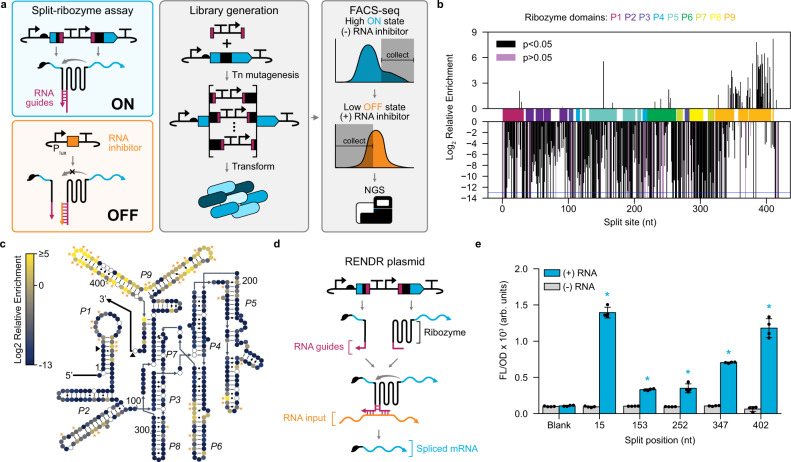
Screening of split ribozymes generates RENDR designs with a high dynamic range of detection. **a** High-throughput screening approach. Left, a split-ribozyme assay to determine splicing from each split ribozyme in the absence (on) and presence (off) of additional stabilizing RNA interactions. RNA guides (magenta) are used to facilitate interactions between ribozyme fragments, which are blocked with an RNA inhibitor (orange). Center, in vitro transposon mutagenesis is used to create a library of split sites. Right, FACS-seq is used to select for split sites with high fluorescence in the on state and low fluorescence in the off state. **b** Relative enrichment of ribozyme split sites following FACS-seq (P1–P9 domains and *p*-values indicated). A two-tailed t-test (alpha = 0.05) was performed on sorted and unsorted DNA frequencies at each split site and resulting *p*-values are provided in the Source Data file. Data below the blue line represent sites lost during selection. **c** Secondary structure of the ribozyme containing enrichment data for each split site (circle fill) and split sites identified through colony screening (orange asterisks). Unfilled circles represent split sites absent from the initial library. **d** RENDR uses an RNA input to template a split ribozyme, producing a spliced mRNA. **e** Characterization of 5 RENDR designs split at identified split sites. Fluorescence characterization (measured in units of fluorescence [FL]/optical density [OD] at 600 nm) was performed in *E. coli* transformed with the RENDR plasmid in the presence and absence of RNA input. Autofluorescence of *E. coli* was determined using cells transformed with empty plasmids (blank). Bars show mean values and error bars represent s.d. of *n* = 4 biological replicates shown as points. To determine significance, a two-tailed t-test (alpha = 0.05) resulted in *p*-values of 0.12, 3.89e−8, 2.89e−8, 1.58e−4, 1.83e−11, and 3.09e−6 for (+) RNA samples Blank, 15, 153, 252, 347, and 402 relative to their (−) RNA counterparts, respectively. Statistically significant differences (*p* < 0.05) are indicated by asterisks above bars. Source data for (**c**) and (**e**) are provided as a Source Data file.

Next, to create a RENDR platform with a high dynamic range of detection, we turned our attention to identifying the optimal ribozyme split sites. An ideal ribozyme split site would destabilize complex formation between the two ribozyme fragments in the absence of RNA input, leading to off states with low signal output. In the presence of the RNA input, this site would have a high level of functional complementation, leading to on states with high signal output. While a few viable split sites within the splicing ribozyme have been identified^22,23^, a systematic analysis of split sites has not been performed. Given the large number of possible split sites (419 variants), we applied a high-throughput, transposon-mediated approach to create a near-complete library of split-ribozyme variants^6^. While this method has widely been used to optimize split proteins, it has not yet been utilized to engineer split RNA. In brief, this method uses a Mu transposase to incorporate a synthetic transposon into every position within the ribozyme sequence. Subsequent cloning steps replace the synthetic transposon with complementary RNA interaction sequences, which we call RNA guides, to create split ribozymes (Fig. 2a and Supplementary Fig. 4). While our ultimate goal was to create a split-ribozyme system that uses an RNA input to template the splicing reaction, we first elected to use two complementary RNA guides that directly bring together the split-ribozyme fragments, a reaction we reasoned would be more efficient and produce a stronger output signal. To allow for screening in the absence of these interactions, we created a ligand-inducible RNA inhibitor sequence to block the interaction between the RNA guides (Fig. 2a). This RNA inhibitor was designed to be the reverse complement of one of the RNA guides and leverages a toehold strategy to favor the disassociation of the functional ribozyme complex (Supplementary Fig. 5). We performed in vitro transposon mutagenesis, confirmed the diversity of this library with next-generation sequencing (NGS), co-transformed the library into *E. coli* with a ligand-inducible RNA inhibitor, and performed fluorescence-activated cell sorting (FACS) to identify variants with high on and low off states in the absence and presence of the RNA inhibitor, respectively (Fig. 2a and Supplementary Fig. 6). Sorted libraries were then sequenced by NGS and the relative enrichment was calculated for each split site (Fig. 2b and Supplementary Fig. 7). To provide additional validation of this approach, we performed manual screening of individual colonies after sorting using bulk fluorescence measurements. Individual variants validated in this way were pooled based on their functionality and sequenced to identify functional split sites (Supplementary Figs. 8, 9). Overlaying these data onto the secondary and tertiary structure of the splicing ribozyme^24,25^, we observed many functional split sites (Fig. 2c and Supplementary Fig. 10). As has been observed for split proteins, functional split sites appear to be particularly enriched in surface-accessible regions within the ribozyme structure^26^. For example, functional split sites are particularly enriched in the P9 domain, a structure that has been experimentally shown to be solvent-accessible^27^. Similarly, split sites appear to be enriched in regions with low sequence conservation^26^. For example, this family of splicing ribozymes (group I introns) are characterized by a conserved core region composed of the helical domains P4–P6 and P3–P7^28^. In general, we observed these conserved regions to have a lower tolerance for split sites, with the exception of the P6 paired region. Taken together, these results suggest that the splicing ribozyme has a tolerance for split sites in specific regions across its sequence, particularly at sites that are surface accessible with low sequence conservation. Additionally, these results demonstrate the value of applying high-throughput protein engineering methodologies to RNA engineering applications.

### Engineering RENDR devices that transduce cellular RNA signals into detectable outputs

Having identified functional ribozyme split sites, we next aimed to create RENDR devices able to convert cellular RNA signals into orthogonal protein outputs. The basic principle of the RENDR platform is to modify a split ribozyme by appending an RNA guide sequence onto each of the two ribozyme fragments. These RNA guide sequences are designed to base pair with an RNA input—which can be a cellular or synthetic RNA—such that, when present, the RNA input co-localizes the two ribozyme fragments, stabilizes the overall structure, and permits splicing (Fig. 2d). To test this, we designed a series of 7 RENDR devices using split sites identified from our data and RNA guides designed to interact with a synthetic RNA input through a total of 163 bp of interaction. Split sites were chosen throughout the structure at hot spots of enrichment and within the P1 domain that has previously been shown to be a functional site for splitting^20^. Fluorescence characterization revealed that five of these RENDR designs were functional, leading to higher fluorescence levels in the presence of the RNA input (Fig. 2e). We note, that two RENDR designs tested showed a relatively low dynamic range of detection (Supplementary Fig. 11). Interestingly, across all designs, we observed consistently low off state levels of fluorescence, indistinguishable from autofluorescence of blank cells, in the absence of the RNA input. Likewise, we observed the highest activation for RENDR designs utilizing splits located at the ends of the ribozyme sequence, which we posit could be due to the structural accessibility of these regions. To confirm that these RENDR designs functioned at the RNA level, as well as to attain a more accurate measure of the dynamic range, we performed RT-qPCR on the high-performing RENDR design, split site 15. We observed a 93-fold increase in the abundance of spliced mRNA in cells expressing both RENDR and RNA input compared to control cells lacking the RNA input (Supplementary Fig. 12). Finally, amplicon sequencing by NGS was used to confirm that the majority of the output was the intended spliced product (Supplementary Fig 13). Taken together, these results confirm the efficacy of using split ribozymes to create high-performing, RNA signal transduction devices.

### Modularity and design rules of RNA input detection

**Fig. 3:**
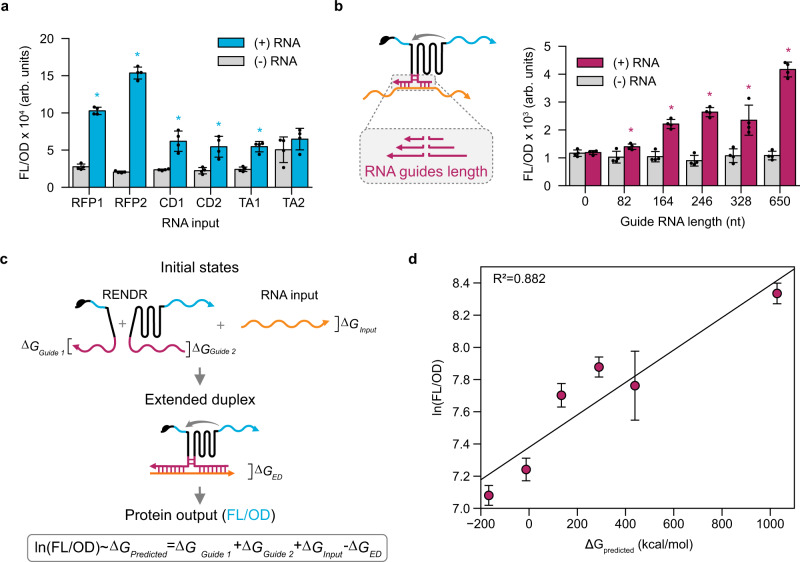
RENDR displays input modularity and RNA guide length predictively influences output. **a** Fluorescence characterization of RENDR variants sensing different RNA inputs from RFP (RFP1/2), CatDe (CD1/2), and TetA (TA1/2). **b** Schematic of RENDR with varying RNA guide lengths and fluorescence characterization of RENDR variants. Fluorescence characterization (measured in units of fluorescence [FL]/optical density [OD] at 600 nm) was performed on *E. coli* transformed with each RENDR design in the presence (magenta) and absence (gray) of a plasmid expressing the RNA input. Autofluorescence of *E. coli* was determined using cells transformed with empty plasmids (blank). **c** Schematic of a thermodynamic model that considers the free energy of the RNA guides and RNA input in their unbound initial states (IS) and an extended duplex (ED) in which they are fully hybridized. Under the hypothesis that the formation of the ED is required for splicing, the natural log of the observed protein output (FL/OD) is proportional to the ΔG_Predicted_, which is the difference in free energies between the IS and the ED. **d** Observed correlations between the fluorescence characterization of protein output in panel (**b**) (natural log FL/OD) and ΔG_Predicted_ of the different RNA guide lengths. Coefficient of determination (*R*^2^) is displayed in the top left corner. Bars in (**a**, **b**) and points in (**d**) represent mean values and error bars represent s.d. of *n* = 4 biological replicates. For panel (**a**), significance was determined using a two-tailed t-test (alpha = 0.05), which resulted in *p*-values of 1.95e−5, 5.4e−8, 1.33e−3, 4.5e−3, 1.97e−4, and 0.25 for (+) RNA samples RFP1, RFP2, CD1, CD2, TA1, TA2 relative to their (−) RNA counterparts, respectively. The same test was used in panel (**b**), with *p*-values of 0.76, 0.019, 7.56e−5, 9.09e−6, 5.1e−3, and 9.7e−7 for (+) RNA samples with guide length 0, 82, 164, 246, 328, and 650 relative to their (−) RNA counterparts, respectively. Statistically significant differences (*p* < 0.05) are indicated by asterisks above bars. Source data for (**a**), (**b**), and (**d**) are provided as a Source Data file.

Having explored the design rules governing how to split the catalytic unit of RENDR, we next investigated the modularity of the RNA input detection reaction. To do this, we created a panel of RENDR variants to detect three different RNA derived from red fluorescent protein (RFP), catechol 2,3-dioxygenase (CatDe), and tetracycline resistance protein (TetA). To allow for the facile redesign of RENDR, a modular cloning strategy was created that allowed for the convenient exchange of different RNA guides (Supplementary Note 1). These RENDR variants were then characterized using an sfGFP output and fluorescence measurements (Fig. 3a). For 5 out of 6 designs, we observed a significant increase in fluorescence when the cognate RNA input was present. To investigate the specificity of RENDR detection, we also performed an orthogonality matrix in which RENDR variants were challenged with non-cognate RNA inputs (Supplementary Fig. 14). Only in the presence of the cognate RNA input did we observe significant activation of RENDR. Taken together these results confirm that RENDR allows for modular and specific detection of different RNA inputs.

We next aimed to investigate how the length of the interaction between RNA guides and the RNA input affects the detection reaction. To do this, we constructed a panel of RENDR devices with different RNA guide lengths against an RNA input. We then used the sfGFP-splicing assay to characterize their performance (Fig. 3b). While we observed a consistently low off state fluorescence compared to the autofluorescence for each design, there were significant differences in the on-state fluorescence values of RENDR devices with different RNA guide lengths. Specifically, we observed an increase in fluorescence as RNA interaction lengths increased. To gain a more quantitative insight into this observation, we constructed a simple thermodynamic sequence-function model that uses available RNA free energy and structure prediction tools^29^, and builds upon the success of other RNA mechanistic models^30–32^. This model relies on the predicted free energies of several states in the RNA detection reaction (Fig. 3c). The initial state (IS) accounts for all RNA species present individually and the extended duplex (ED) represents the state where the RNA guides and the RNA input are fully hybridized. Based on our experimental data, we hypothesized that formation of the ED would promote complementation and folding of the split ribozyme. With these assumptions, we predicted that the rate of splicing, and hence gene expression, is proportional to the formation of the ED between the RNA input and the two RNA guides (Supplementary Note 2).

Deriving this model, we predicted that the natural log of the observed gene expression (fluorescence (FL)/optical density at 600 nm (OD)) is linearly related to the difference in free energy between the IS and the ED. For this analysis, we only considered folding and interactions of the two RNA guides attached to each ribozyme fragment and the RNA input:

1 $${{{{{\rm{ln}}}}}}({{{{{\rm{FL}}}}}}/{{{{{\rm{OD}}}}}}) \sim {\Delta {G}}_{{G}{{{{{\rm{uide}}}}}}1}+{\Delta {G}}_{{G}{{{{{\rm{uide}}}}}}2}+{\Delta {G}}_{{{{{{\rm{RNA}}}}}}{{{{{\rm{input}}}}}}}-{\Delta {G}}_{{{{{{\rm{ED}}}}}}}.$$

This free energy difference naturally reflects the competing effects of intramolecular base pairs that need to be broken before the formation of intermolecular base pairs that lead to the ED and, ultimately, the active ribozyme state. A comparison of the expression levels predicted by the model to the corresponding experimentally observed fluorescence levels shows a strong correlation (*R*^2^ = 0.882) (Fig. 3d). To validate this observation and model, we performed two additional experiments. First, we performed an analogous experiment using a distinct RNA input derived from a dCas9 gene. From this experiment, we observed a linear relation between RNA guide length and output, and a strong correlation from our model (*R*^2^ = 0.846) (Supplementary Fig. 15a, b). Second, we characterized a panel of RENDR variants with a fixed RNA guide length that contained different percentages of base pair mismatch to the RNA input. We observed an increase in output with increasing base pair interactions in the ED, which resulted in a strong correlation with our model (*R*^2^ = 0.776) (Supplementary Fig. 15c, d). Finally, to begin to understand the input-output relationship of RENDR, we measured RENDR outputs with varying levels of input RNA produced from an inducible promoter (Supplementary Fig. 16). Taken together, these results begin to uncover the quantitative design rules of RENDR and provide a theoretical basis for future design rules and predictive models.

### A modular protein output strategy for RENDR

**Fig. 4:**
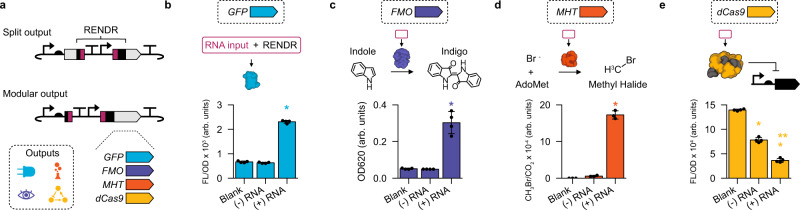
A modular RENDR design allows for interchangeable protein outputs in response to RNA inputs. **a** Schematic of a non-modular output design that depends upon gene-specific splits in the CDS (top) and a schematic of a modular output design that uses RENDR to separate the RBS from any CDS output (bottom). The modular design facilitates the simple exchange of different outputs that produce fluorescence, gaseous, colorimetric, and regulatory outputs. **b** Fluorescent characterization (measured in units of fluorescence [FL]/optical density [OD] at 600 nm) from RENDR using an sfGFP output. **c** Characterization of indigo production (measured in optical density [OD] at 620 nm) from RENDR using a flavin monooxygenase (FMO) output. **d** Characterization of methyl-bromine gas production (measured in CH_3_Br/CO_2_) from RENDR using a methyl-halide transferase (MHT) output. **e** Characterization of a dCas9 protein output from RENDR measured using an sfGFP-repressing CRISPR interference assay in cells containing a genomically integrated sfGFP expression cassette. For (**b**–**e**), characterization was performed in *E. coli* transformed with each RENDR design in the presence (+) and absence (−) of a plasmid expressing the RNA input. Background signal from *E. coli* was determined using cells transformed with empty plasmids (blank) for each output. In (**e**), blank uses *E. coli* containing chromosomally integrated sfGFP expression cassette. Bars show mean values and error bars represent s.d. of *n* = 4 biological replicates shown as points. Asterisks indicate *p*-value <0.05 relative to blank. Significance was determined with a two-tailed t-test (alpha = 0.05), which resulted in *p*-values of 3.27e−9, 1.52e−4, and 1.47e−5 for (+) RNA samples in (**b**)–(**d**) relative to their (−) RNA counterparts, respectively. The same test was used in (**e**), where *p*-values were 4.34e−7, 6.87e−9, and 1.33e−5 for (−) RNA relative to Blank, (+) RNA relative to Blank, and (+) RNA relative to (−) RNA, respectively. Statistically significant differences (*p* < 0.05) are indicated by asterisks and double asterisks (for (+) RNA relative to Blank in panel (**e**)) above bars. Source data for (**b**)−(**e**) are provided as a Source Data file.

We next aimed to create a modular gene output design for the RENDR platform that would allow for simple exchange of the output protein being used. To do this, we redesigned the split ribozyme to be located between the RBS and the CDS of the output protein (Fig. 4a), resulting in a more modular design that eliminates the need to identify a ribozyme insertion site in each new protein-output gene. This design greatly simplifies and generalizes the process of coupling RENDR with different biochemical processes to adapt the platform for different applications. To confirm the functionality of the modular design, we first tested it using a fluorescent sfGFP output (Fig. 4b). Following this, we used RENDR to control the biosynthesis of indigo, a chemical commodity that can also serve as a colorimetric output. We combined RENDR with the flavin-containing monooxygenase (*FMO*) gene from *Methylophaga aminisulfidivorans st. MP*^33^, which catalyzes the conversion of tryptophan-derived indole into indoxyl, which in turn spontaneously forms indigo. Characterization of this design (RENDR-FMO) in *E. coli* revealed tight control of indigo production in the absence of the RNA input, and strong induction of indigo synthesis in the presence of the RNA input (Fig. 4c and Supplementary Fig. 17). Next, we aimed to use RENDR to control a gas-producing enzyme, methyl halide transferase (MHT), which produces methyl-bromide (CH_3_Br) from S-adenosyl methionine (AdoMet) and bromide, and has been used as a reporter system for measuring gene expression in complex and non-transparent matrices like soils^34^. Characterization by GC/MS revealed RENDR-MHT could effectively control the production of CH_3_Br (Fig. 4d). We then coupled RENDR with a CRISPR-Cas system, which is widely used in a variety of gene regulation and editing applications^35,36^. Specifically, we employed a CRISPR interference (CRISPRi) assay, which uses a catalytically dead Cas9 (dCas9) protein to transcriptionally repress a genomically-encoded *sfGFP* gene, placing production of the dCas9 under the control of RENDR (RENDR-dCas9). This assay demonstrated strong repression in the presence of the RNA input, suggesting that dCas9 production can be regulated by an RNA signal using RENDR (Fig. 4e). We did observe slight repression from RENDR in the absence of the RNA input, implying that RENDR does not fully prevent the expression of dCas9 and that these small levels are sufficient to repress the single *sfGFP* copy within the *E. coli* genome. Additionally, we coupled RENDR to the production of T7 RNA polymerase (T7 RNAP)—a widely used component for gene expression control and genetic circuit design^13,37,38^. Measuring T7 RNAP production from a modular RENDR design using a T7 promoter-sf*GFP* reporter, we initially noticed a high leak in the off state and significant toxicity (e.g., slow growth rates). To resolve this, we opted for a split T7 RNAP design^13^ containing an R632S mutation previously shown to reduce toxicity^39^. These modifications allowed us to successfully couple RENDR to control the production of T7 RNAP (Supplementary Fig. 18). Taken together, these results show that RENDR offers output modularity and can transduce cellular RNA input signals into the production of fluorescent, colorimetric, gaseous, and regulatory outputs.

### RENDR is functionally portable across different bacteria

**Fig. 5:**
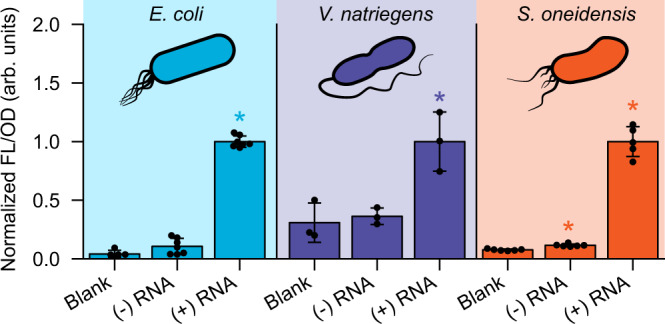
RENDR is portable across different Gram-negative bacteria. Characterization of RENDR across three different Gram-negative bacteria. Fluorescence characterization (measured in units of fluorescence [FL]/optical density [OD] at 600 nm) was performed in *E. coli*, *V. natriegens*, and *S*. *oneidensis* transformed with the RENDR plasmid in the presence (+) and absence (−) of RNA input. Autofluorescence was determined using cells transformed with empty plasmids (blank). Bars show normalized mean values and error bars represent s.d. of *n* = 7, *n* = 3, and *n* = 6 biological replicates shown as points for *E. coli*, *V. natriegens*, and *S. oneidensis*, respectively. For the Blank *E. coli* condition and the (+) *S. oneidensis* condition, *n* = 5, Significance was determined using a two-tailed t-test (alpha=0.05), which resulted in *p*-values of 2.2e−12, 0.013, and 3.4e−8 for (+) RNA samples in *E. coli*, *V. natriegens*, and *S. oneidensis* relative to (−) RNA, respectively. Statistically significant differences (*p* < 0.05) are indicated by asterisks above bars. Source data are provided as a Source Data file.

As the splicing ribozyme depends upon universal RNA interactions and catalysis, we posited that RENDR could be used in other bacterial species with minimal customization. To test this, we characterized a RENDR-GFP plasmid in three different Gram-negative species: *E. coli, Shewanella oneidensis, and Vibrio natriegens*. No genetic modifications were made to the RENDR-encoding plasmid that was transformed into each species. Fluorescence characterization revealed that across all three species, RENDR was functional—showing high fluorescence in the presence of the RNA input and low fluorescence in absence of the RNA input (Fig. 5). Taken together, these results show that RENDR is portable across different Gram-negative bacteria and likely will require minimal customization to be ported into new microbial hosts.

### RENDR allows for facile colorimetric detection of antibiotic-resistant microbes

**Fig. 6:**
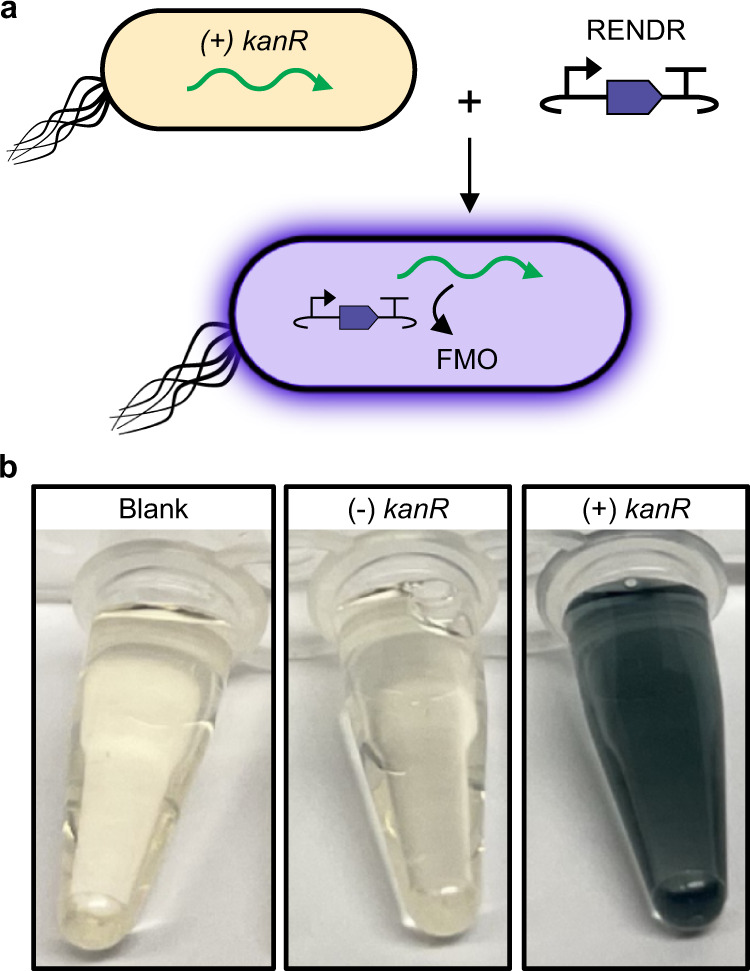
RENDR enables robust colorimetric detection of antibiotic-resistant microbes. **a** Schematic of using RENDR to detect kanamycin-resistant cells. RENDR was transformed into *E. coli* containing (+kanR) or lacking (−kanR) a plasmid expressing a kanamycin resistance gene. When kanR mRNA is present, it activates RENDR, allowing for the production of an FMO output that catalyzes the production of indigo. **b** Photos of indigo extracted from each sample culture. Source data for (**b**) are provided as a Source Data file.

As a proof of principle application, we wanted to demonstrate that RENDR could be used to create a simple, low-cost system that detects antibiotic-resistant microbes. Our idea was to create a DNA plasmid that when introduced into a target bacteria would sense the presence of an antibiotic resistance gene (ARG) and in response output a pigment-producing enzyme that would allow for simple colorimetric detection by eye. Leveraging the plug-and-play nature of RENDR, we exchanged the RNA sensing components to detect a kanamycin resistance (*kanR*) gene and used the indigo-producing gene, *FMO*, as an output (Fig. 6a). When *E. coli* cells containing the *kanR* gene were transformed with this plasmid and grown, we observed dramatic indigo production that was clearly visible to the naked eye, whereas in the absence of ARG we saw no visible indigo production (Fig. 6b and Supplementary Fig. 19). Thus, these results show that RENDR can be used to sense antibiotic resistance phenotypes through the detection of native cellular mRNAs.

## Discussion

Here we show the development and characterization of RENDR, a ribozyme-based platform that uses individual cellular RNA to template splicing reactions, which in turn produce orthogonal protein outputs. Inspired by protein engineering efforts, we used a laboratory evolution approach to construct a library of split-ribozyme variants and identified high-performing split sites using a high-throughput FACS-seq screen. To allow for easy redesign of RENDR, we determined the optimal RNA guide length and derived a thermodynamic model to further characterize the RENDR splicing reaction. We then demonstrated the modularity of the system by repositioning the split ribozyme upstream of the CDS and replacing *sfGFP* with other protein-coding sequences, yielding an array of fluorescent, colorimetric, gaseous, and regulatory outputs. We showed that RENDR is functionally portable across different Gram-negative bacteria without any modification to the expression cassette. Thus, RENDR is a highly modular, designable, portable, and high-performing platform that addresses the limitations of existing split-aptamer and split-ribozyme RNA detection platforms^20^. Finally, we used our optimized system to detect native mRNA that encodes an antibiotic resistance protein, creating a facile approach for the detection of antibiotic-resistant microbes. This work shows how specific transcripts can be detected in situ and converted into different types of biochemical information using an RNA synthetic biology tool.

RENDR delivers a high-performing, plug-and-play RNA detection platform that complements current technologies for transducing RNA signals for cellular programming applications. For example, RNA-responsive CRISPR-Cas single guide RNAs (sgRNAs) have been engineered to form self-inhibiting structures that can be released by a strand displacement reaction with a target RNA^17,40–42^, providing a route for coupling CRISPR-Cas activity to a specific RNA target. While powerful, the broad utility of this tool is limited because it can only control the activity of the corresponding Cas protein. These mechanisms have also been limited in the length of sequence they can detect (~30–55 bp), which we posit could limit the specificity of detection. Our RENDR platform addresses these limitations by allowing for the control of theoretically any protein output and yields a high dynamic range of sensing of longer RNA sequences. RENDR could also be used in conjunction with RNA-responsive sgRNAs to exert an additional layer of control. For example, a multiple RNA input AND gate can be generated by pairing current RNA-regulated sgRNA technologies with RENDR-activated Cas proteins. This implementation, and other combinations of RNA tools, could be impactful in adding regulatory specificity to these systems and reducing Cas-derived burden on cells.

The toehold switch, another RNA-sensing device, allows access to the RBS, and consequently translation, of an mRNA sequence in response to an RNA target through a programmable strand displacement reaction^18^. Like RENDR, toehold switches can control a diversity of protein outputs and offer a high dynamic range of sensing in a variety of contexts. However, toehold switch designs require the addition of amino acids to the start of the CDS, which could interfere with protein structure-function and consequently limit the modularity of these systems. RENDR is not bound by this requirement and may be better suited to regulate a wide variety of protein outputs, Additionally, unlike translation-based RNA-sensor technologies, RENDR is not limited to protein outputs and can in principle be coupled to outputs mediated by nucleic acids (e.g., CRISPR RNAs, non-coding small RNA regulators, and fluorescent aptamers). Finally, toehold switches have only been functionally demonstrated in *E. coli*. As we have shown, RENDR is functionally portable across different Gram-negative bacteria without any genetic modification, and we anticipate that it could be easily ported into other classes of microbes and eventually mammalian cells. This is an important advantage of RENDR, which we believe will facilitate efforts to expand synthetic biology into non-model chassis and consortia. While powerful, we recognize further work is required to achieve the full potential of RENDR. Using RENDR to detect low-abundant endogenous transcripts will require more detailed investigations of the sensitivity and potentially require optimization of the system. Taken together, RENDR expands and complements the RNA synthetic biology toolbox for the transduction of native RNA for cellular programming.

Within the field of RNA synthetic biology, topological engineering of large RNA represents a currently untapped route to diversify and optimize this biomolecule. To date, most synthetic RNA technologies have focused on the rational and computational design of small, non-coding RNA regulators such as small RNA, riboswitches, small self-cleaving ribozymes, and CRISPR RNA^43^. Here we highlight the potential of engineering large catalytic RNA for synthetic biology applications using laboratory evolution. Specifically, we demonstrate how theoretical and experimental approaches from protein engineering can be easily leveraged to rapidly engineer structurally complex RNA for new capabilities. For example, while in vitro transposon mutagenesis coupled with high-throughput screening has been widely applied to create split, fused, and circularly permutated proteins^6^, our work represents the first attempt to systematically apply this method for engineering synthetic RNA functions. While we have demonstrated the power of applying these approaches toward creating RNA-sensing split splicing ribozymes, we expect that this is just the beginning. Circular permutation of RNA, for example, would offer distinct topological modifications, which in protein engineering have been used to yield proteins that are optimized for fusion^44^, have synthetic sensing capabilities^45^, or offer enhanced and expanded functional characteristics compared to wild-type topologies^46,47^. Similarly, topological engineering could be applied to other natural and synthetic ribozymes offering different chemistries or catalytic properties^48^. Thus, in the coming years, we envision topological engineering of large catalytic RNA to be used to further expand the structure-function landscape of RNA tools.

Dynamic monitoring of RNA in living cells is a valuable sensing modality. Understanding temporal patterns of transcription is critical to uncovering a deeper understanding of gene regulatory networks and the processes they control^49^. While this can be achieved by labeling endogenous genes with fluorescent markers (e.g., fluorescent proteins or aptamers), RENDR provides an alternative approach to achieve this that circumvents the need for time-consuming and arduous genome engineering. Compared to other RNA-templated detection reactions, such as fluorescence in situ hybridization (FISH) that depend upon the use of fixed cells, RENDR can be deployed in living cells and allows for dynamic measurements. Beyond direct RNA monitoring, we anticipate RENDR will see broad utility for implementing genetic programs in response to cell phenotype, physiology, and identity. For example, in bacteria, coupling genetic programs to species-specific RNA signatures using RENDR would provide a route to create genetic programs that only function in the desired target host, a goal that is difficult to achieve using traditional regulatory elements (e.g., promoters and RBSs) that often function across related species. Similarly, by coupling genetic programs to specific RNA signatures, we anticipate it will be possible to use RENDR to implement burden- and growth-driven feedback within genetic programs. Such feedback control systems are of critical importance to achieve robustness in engineered functions under fluctuating demands for resources and differing environmental conditions^50^, and to optimize metabolic processes^51^. Finally, we anticipate that RENDR could be an impactful tool for mammalian synthetic biology to, for example, create therapeutic genetic programs that sense and respond to biomarkers of disease^52^. Taken together, we predict that the broad utility of RENDR will advance both basic science and application-driven research in the coming years.

## Methods

### Plasmid assembly and strains

All plasmids used in this study are listed in Supplementary Table 1, with key sequences provided in Supplementary Tables 2–5 and Supplementary Fig. 1. Bacterial strains used are listed in Supplementary Table 6. Methods used to clone plasmids included inverse PCR, Gibson Assembly, and Golden Gate assembly. All plasmid constructs were verified using Sanger sequencing or full-plasmid sequencing.

### Fluorescence measurements

Fluorescence measurements were performed in various transformed *E. coli, V. natriegens, and S. oneidensis* strains according to Supplementary Table 6 and as described below. For experiments using *E. coli*, plasmids were transformed into chemically competent *Escherichia coli* str. K-12 substr. MG1655 (*E. coli* MG1655) or TG1 cells, plated on LB-agar plates containing the appropriate antibiotics (LB-agar-Ab) according to Supplementary Table 7, and incubated overnight at 37 °C. Following incubation, colonies were used to inoculate 300 µL of LB media containing appropriate antibiotics (LB-Ab) in a deep 96-well plate and incubated at 37 °C at 1000 rpm overnight. Following incubation, 10 µL of each overnight culture was used to inoculate 290 µL of pre-warmed LB-Ab and grown at 37 °C and 1000 rpm for 8 h (hr). For experiments using N-(β-ketocaproyl)-L-Homoserine lactone (AHL)- and arabinose-inducible plasmids, 4 µL of overnight culture was added to 296 μL of pre-warmed LB-Ab and grown at 37 °C at 1000 rpm for 4 h. This pre-culture was then diluted 1:50 in fresh, pre-warmed LB-Ab with either 1 µM AHL or 1% (w/v) arabinose (Alfa Aesar) and grown at 37 °C at 1000 rpm for 6 h. Bulk fluorescence measurements were performed on 50 µL of experimental culture diluted in 50 µL of phosphate buffered saline (PBS, (Fisher)) in a 96-well plate. For experiments using *V. natriegens*, plasmids were electroporated into electrocompetent cells made from *V. natriegens* cells mixed in electroporation buffer (680 mM sucrose, 7 mM K_2_HPO_4_, pH 7) and pulsed at 0.9 kV using a MicroPulser Electroporator (Bio-rad). Electroporated cells were recovered at 37 °C for 1 h, plated onto LB3-agar (25 g/L LB broth (Fisher), 20 g/L NaCl (Fisher), 15 g/L Agar (Fisher)) with antibiotics according to Supplementary Table 7, and incubated for 48 h at 37 °C. Colonies were picked in biological triplicate and grown for 24 h in LB3-Ab media at 37 °C. Following growth, cells were washed by centrifuging at 15,800 × *g* for 1 min and resuspending in with the same volume of PBS. Bulk fluorescence measurements were performed on 50 µL of washed culture diluted in 50 µL of PBS. For experiments using *S. oneidensis*, plasmids were electroporated into electrocompetent cells made from *S. oneidensis* cells washed 3 times with 10% glycerol and pulsed at 1.2 kV using the same electroporator as above. Electroporated cells were recovered at 30 °C for 2 h, plated onto LB-agar-Ab plates according to Supplementary Table 7, and incubated at 30 °C for 24 h. Six biological replicate colonies were picked from plates and inoculated into 300 µL of LB-Ab in a deep 96-well plate and incubated at 30 °C at 1000 rpm overnight. Following incubation, 10 µL of each overnight culture was used to inoculate 290 µL of pre-warmed LB-Ab and grown at 30 °C and 1000 rpm for 9 h. Bulk fluorescence measurements were performed on 50 µL of washed culture diluted in 50 µL of PBS. For all bulk fluorescence measurements, optical density (OD) at 600 nm and sfGFP fluorescence (FL) (excitation: 485 nm, emission: 520 nm) was measured using a Tecan M1000 (Tecan i-control common software (v3.7.3.0)) or Tecan SPARK (SPARKCONTROL Method Editor software (v3.0)) plate reader. For flow cytometry measurements, cultures were diluted 1:100 in PBS and analyzed on a Sony Biotechnology SH800 Cell Sorter. 50,000 events were collected unless otherwise noted.

### Bulk fluorescence data analysis

Each 96-well block included at least two sets of controls; a media blank and *E. coli, V. natriegens, or S. oneidensis* transformed with combinations of empty control plasmids pJEC101, pJEC102, or pJEC103, referred to here as blank cells. Blank cells do not express reporter genes, only antibiotic resistance genes, and were used to determine autofluorescence levels. OD and FL values for each colony were first corrected for by subtracting the mean value of the media blank from the respective value of the experimental conditions. The ratio of the corrected FL to the corrected OD (FL/OD) was then calculated for each well.

### Flow cytometry data analysis

FCS files containing flow cytometry data collected on the Sony Biotechnology SH800 Cell Sorter were analyzed using a custom Python script. In brief, FCS files were imported and cells were gated using a high-low gate on forward scatter vs side scatter plots using the FlowCal (v1.3.0) package [https://taborlab.github.io/FlowCal/]. Gated biological replicates were combined into a single dataset and resulting data was plotted using the Matplotlib (v3.6.2) package [https://matplotlib.org/].

### Split-ribozyme library

The library of split-ribozyme variants was created using a previously described Mu transposase method^6,53^ (Supplementary Fig. 4). In brief, transposon DNA was prepared by digesting 2 µg of plasmid pBW001 (addgene #131529) with HindIII and Bglll at 37 °C for 6 h, followed by purification. Transposon insertion reactions were then prepared using 50 fmol of purified transposon, 100 fmol of a ribozyme-containing plasmid, 5× MuA buffer, and 0.22 µg of MuA transposase (ThermoFisher Scientific). The reaction was incubated at 30 °C for 16 h, followed by heat inactivation at 75 °C for 10 min and purification. Purified transposon-inserted ribozyme library was then transformed into chemically competent *E. coli* MG1655 cells. A small sample (0.5% of total volume) of the transformation was plated on LB-agar-Ab and the resulting colonies were used to calculate transformation efficiency. The remaining transformation was used to inoculate 50 mL of LB media containing spectinomycin (50 µg/mL) and kanamycin (100 µg/mL), which was grown at 37 °C at 250 rpm overnight, after which library plasmids were isolated using a DNA midi prep kit. Transposon-inserted ribozyme fragments were isolated by digesting 2 µg of transposon-inserted ribozyme plasmids with BbsI-HF at 37 °C for 2 h, followed by agarose gel purification. The purified transposon-inserted ribozyme variants were then cloned into a plasmid containing a sfGFP gene, which was split after the first nucleotide in amino acid Y66. The transposon was replaced with a linear DNA sequence containing the interacting RNA guide sequences and transformed using the same method as above. The diversity of the cloned library was determined by using PCR to generate amplicons containing the 5’ fragment of the split-ribozyme. After column purification, the PCR product was sent for next generation sequencing (NGS) (Amplicon-EZ, GeneWiz) (Supplementary Fig. 6).

### FACS-seq

The split-ribozyme library was co-transformed into *E. coli* MG1655 with plasmid pJEC758, which encodes an AHL-inducible RNA inhibitor. These cells were then subjected to three rounds of fluorescence activated cell sorting (FACS) to enrich for functional split variants. In the first round of sorting, 100 µL of co-transformed library was used to inoculate 5 mL of LB-Ab and grown overnight at 37 °C at 250 rpm. Cells were diluted 1:50 and grown in a pre-culture for 4 h, then re-diluted 1:50, and grown in culture for 6 h without AHL. Cells were placed on ice and diluted 1:100 in PBS before being analyzed on a Sony Biotechnology SH800 Cell Sorter. A gate was set to isolate the most fluorescent cells (top 14% of the population) and 673,881 cells were collected in 5 mL LB-Ab. Sorted cells were then incubated at 37 °C at 250 rpm for 1 h, the volume adjusted to 15 mL using LB-Ab, and grown overnight at 37 °C at 250 rpm. In the second round of sorting, 100 µL of the overnight culture from the first sort were grown in 5 mL LB-Ab using the same protocol used in the first round, with the addition of AHL to the culture at a final concentration of 1 µM for the 6 h growth period. After growth and dilution in PBS, a gate was set containing the least fluorescent cells (bottom 85% of the population) and 1 million cells were collected in 5 mL LB-Ab. The culture was adjusted to 10 mL with LB-Ab, recovered at 37 °C at 250 rpm for 1 h, re-adjusted to 15 mL with LB-Ab, and grown overnight at 37 °C at 250 rpm. In the third round of sorting, 100 μL of saturated culture started from cells collected in the second round of sorting were used to inoculate a 5 mL LB-Ab pre-culture and the same protocol was followed that was used in the first round of sorting. A gate was set to isolate the most highly fluorescent cells and 500,000 cells were collected and incubated as in the first round of sorting. A sample of the sorted cells was serially diluted and plated on LB-agar-Ab overnight at 37 °C. The remaining sorted cells were grown overnight in LB-Ab at 37 °C at 250 rpm, after which glycerol stocks were made and stored at −80 °C.

To perform individual variant screening (Supplementary Fig. 8), colonies were picked from serial dilution plates or from plates streaked from glycerol stocks of the sorted library, used to inoculate 300 µL LB-Ab in 96 deep-well plates, and incubated overnight at 37 °C at 1000 rpm. Following overnight incubation, 4 µL of each overnight culture was used to inoculate 196 µL of pre-warmed LB-Ab with or without 0.2 µM AHL and grown for 7 h at 37 °C at 1000 rpm. After growth, glycerol stocks were made of each culture and duplicate bulk fluorescence measurements performed. We note that some variants were isolated from cultures subjected to a fourth round of sorting following the protocol outlined above, which was used to isolate highly fluorescent cells. From these measurements, each colony was scored based on their on and off states (Supplementary Fig. 8). A distance from the *x* = *y* line value was calculated for each variant, where Distance = ([FL/OD (-AHL)] - [FL/OD (+AHL)])/sqrt(2), and variants were split into tertiles based on their distance. Tertiles for each replicate were compared across duplicates and variants in the top two tertiles in both replicates were considered functional. Functionally-validated variants were pooled from glycerol stocks into 45 mL LB-Ab cultures and grown overnight at 37 °C at 250 rpm. Plasmid DNA of the pooled culture was isolated using a Midiprep Kit (Qiagen) and PCR was used to generate amplicons containing the 5’ fragment of the split-ribozyme. After column purification, the PCR product was sent for NGS (Amplicon-EZ, GeneWiz) (Supplementary Fig. 9B).

### Next generation sequencing data analysis

FASTQ files from NGS were processed using a custom Python script described in Supplementary Note 3.

### Calculation of free energies for guide length variants

A locally-installed version of the Nucleic Acids Package (NUPACK) version 4.0.0.27^54^ was used to calculate free energy parameters for the thermodynamic model (Supplementary Note 2).

### FMO output characterization

Plasmids were transformed as described for fluorescence measurements. Colonies (biological quadruplicates) were used to inoculate 300 µL of LB-Ab in a 96 deep-well plate overnight at 37 °C at 1000 rpm. For each overnight culture, 100 µL was transferred to a 5 mL LB-Ab culture containing 5 mM tryptophan and grown to saturation at 37 °C at 250 rpm for 24 h. Indigo was extracted from cultures using dimethyl sulfoxide (DMSO)^55^. Briefly, 1.5 mL of each cell culture was centrifuged for 10 min at 13,000 × *g*. Pellets were resuspended in 100 µL water, 1 mL of DMSO was added, and samples were vortexed. To quantify the yield, 100 µL of extracted indigo was added to a 96-well plate and the OD at 620 nm was measured for all samples. An optimized growth protocol was used for characterization in the experiment performed in Fig. 6 where cells were grown under the same conditions, except M9 media (1× M9 salts, 1 mM thiamine hydrochloride, 0.4% glycerol, 0.2% casamino acids, 2 mM MgSO_4_, 0.1 mM CaCl_2_) with appropriate antibiotics was used, cultures were grown for 48 h instead of 24 h, and indigo was extracted from 5 mL of culture.

### MHT output characterization

Plasmids were transformed as described for fluorescence measurements. Colonies (biological triplicates) were used to inoculate 50 mL tubes with 5 mL LB-Ab and grown overnight at 37 °C at 250 rpm. Cells were washed and normalized to a fixed OD before being transferred to GC-MS vials. Briefly, 1 mL of each overnight culture washed three times by centrifugation at 13,000 × *g* for 1 min followed by resuspension in 1 mL M63 medium (250 µL 1 M MgSO_4_, 2.5 mL 20% glucose, 25 µL 0.5% thiamine, 1.25 mL 10% Bacto casamino acids, 250 µL 1000× spectinomycin, 250 µL 1000× carbenicillin, 6.25 mL 4 M NaBr, 50 mL 5× M63 salts [10 g (NH_4_)_2_SO_4_, 68 g KH_2_PO_4_, 0.00025 g FeSO_4_.7H_2_O, 30 mL 6 M KOH to pH = 7, water up to 1 L], and water up to 250 mL). Washed cells were diluted to OD 600 = 0.05 with M63 medium in a final volume of 1 mL in 2 mL Phenomenex glass vials and subsequently crimped every 2.5 min to account for the time it takes the Gas Chromatography/Mass Spectrometry (GC/MS) instrument to process each sample. Crimped glass vials were grown at 37 °C at 250 rpm for 6 h. A GC/MS containing an Agilent 8890 gas chromatograph fit with an Agilent 7693A autosampler and a 5977B mass spectrometer was used to measure samples. The Agilent MassHunter Workstation Quantitative Analysis software (v10.0) was used to quantify resulting methyl-bromide and carbon dioxide (CO_2_) production. To account for cell density, the methyl-bromide production was normalized to CO_2_ concentration for each sample.

#### Statistical analysis

The sample average and standard deviation (s.d.) were calculated from the replicates of each sample in all experiments. Significance in all experiments was assessed using a homoscedastic two-sample t-Test with an alpha of 0.05. In all cases, resulting *p*-values of less than 0.05 for experimental sample measurements compared to blank cell measurements were considered significantly different.

### Total RNA extraction for reverse transcription quantitative PCR (RT-qPCR)

RNA extractions and quantitative PCR measurements were performed in biological triplicate using *E. coli* strain MG1655. Plasmids were transformed and colonies were used to inoculate 5 mL of LB-Ab and incubated at 37 °C at 250 rpm overnight. Following incubation, 100 µL of each overnight culture was used to inoculate 5 mL of pre-warmed LB-Ab and grown for 6 h. After growth, 500 µL of each culture was centrifuged at 13,000 × *g* for 1 min and the supernatant removed. Pellets were resuspended in 750 µL of Trizol reagent (Thermo Fisher Scientific) and incubated at room temperature for 5 min, then 150 µL of chloroform was added, mixed by vortexing for 15 s, and incubated at room temperature for 3 min. After incubation, samples were centrifuged for 15 min at 12,000 × *g* at 4 °C and the top aqueous layer isolated and 1 µL of RNA-grade glycogen (Thermo Fisher Scientific, 20 mg/mL) and 375 µL of isopropanol were added. The solution was then incubated at room temperature for 10 min and centrifuged at 4 °C at 13,000 × *g* for 15 min. After centrifugation, the isopropanol was carefully removed, pellets were washed in 600 µL of chilled 70% ethanol, and samples were centrifuged at 4 °C at 13,000 × *g* for 2 min. Following removal of the ethanol, the pellets were centrifuged again at 4 °C at 13,000 × *g* for 2 min to ensure the complete removal of the ethanol. Pellets were air dried for 5 min and resuspended in 18 µL of RNase-free water.

### DNase treatment of total RNA for RT-qPCR

The concentration of all samples was measured with a Qubit fluorometer (Thermo Fisher Scientific). For each sample, 1.5 µg of total RNA (for a final concentration of 30 ng/µL) was digested by Turbo DNase (Thermo Fisher Scientific) according to the manufacturer’s instructions. After digestion, 150 µL of RNase-free water and 200 µL of phenol-chloroform were added to each tube. The samples were then mixed by vortexing for 10 s, incubated at room temperature for 3 min, and centrifuged at 13,000 × *g* at 4 °C for 10 min. After centrifugation, 190 µL of the top aqueous layer was removed and added to 190 µL of chloroform. Samples were vortexed for 10 s, incubated for 3 min at room temperature, and centrifuged at 13,000 × *g* at 4 °C for 10 min. After centrifugation, 170 µL of the top aqueous layer was removed and added to 170 µL of chloroform. Samples were vortexed for 10 s, incubated for 3 min at room temperature, and centrifuged at 13,000 × *g* at 4 °C for 10 min. After centrifugation, 120 µL of the top aqueous layer was removed and mixed with 1 µL of RNA-grade glycogen, 360 µL of chilled 100% ethanol, and 12 µL of 3 M sodium acetate. Samples were mixed by pipetting and incubated at −80 °C for at least 1 h. After incubation, the samples were centrifuged for 30 min at 13,000 × *g* at 4 °C. After removing the supernatant, the resulting RNA pellets were washed with 600 µL of chilled 70% ethanol and centrifuged at 4 °C at 13,000 × *g* for 10 min. After centrifugation, the ethanol was removed, and the pellets were centrifuged at 4 °C at 13,000 × *g* for 2 min. Ethanol wash was repeated a second time to ensure the complete removal of any remaining ethanol. Pellets were air dried for 5 min and resuspended in 12 µL of RNase-free water.

### Reverse transcription and qPCR measurements

The concentration of all samples was measured with a Qubit fluorometer. To anneal primers for cDNA synthesis reactions, 75 ng of total RNA, 0.5 µL of 2 µM reverse transcription primer, 1 µL of 10 mM dNTPs (New England BioLabs), and RNase-free water (up to 6.5 µl) were combined. This mixture was then incubated at 65 °C for 5 min and cooled on ice for 5 min. For reverse transcription, 0.25 µL of Superscript III reverse transcriptase (Thermo Fisher Scientific), 1 μL of 100 mM Dithiothreitol (DTT), 1× first-strand buffer (Thermo Fisher Scientific), 0.5 µL RNaseOUT (Thermo Fisher Scientific), and RNase-free water up to 3.5 µL were then added to the annealed primer reaction, and the total mixture was incubated at 55 °C for 1 h, 75 °C for 15 min, and then stored at −20 °C. The resulting cDNA samples were subsequently analyzed via qPCR on a CFX Connect Real-Time PCR system (Bio-Rad). Each biological sample for each condition was analyzed in three technical replicates in a 96-well microplate covered with an optically clear seal. Total RNA samples were also analyzed as non-reverse transcription controls (NRT) to confirm that no DNA could be detected under the conditions used. Additionally, a non-template control was run to confirm that no non-specific amplification was present. For each technical replicate, a reaction was prepared by mixing 5 µL of Maxima SYBR green qPCR master mix (Thermo Fisher Scientific), 2 µL of cDNA and 0.5 µL of 2 µM forward and reverse sfGFP qPCR primers, and RNase-free water up to 10 µL. For the reaction, the following program was used: 50 °C for 2 min, 95 °C for 10 min, followed by 30 cycles of 95 °C for 15 s and 60 °C for 1 min. Melting curve analysis was run after all measurements to ensure that only a single product was amplified. For quantification, a standard curve covering four ten-fold serial dilutions of an *sfGFP* standard was analyzed in parallel. The standard curve was used to determine the relative abundance of sfGFP cDNA in all tested samples and to infer the efficiency of the qPCR primer set (85%). Results were analyzed using CFX Manager Software (v3.1) (Biorad). Briefly, the standard curve was used to determine the starting quantity (SQ) of the sfGFP cDNA in each reaction. The SQ values of technical replicates for each sample were then averaged to give a single value for each biological replicate. The mean and s.d of biological replicates were then calculated for each sample type.

### Reporting summary

Further information on research design is available in the Nature Portfolio Reporting Summary linked to this article.

## Supplementary information

Supplementary Information

Reporting Summary

## Data Availability

Source data are provided with this paper. The sequencing data generated in this study have been deposited in the NCBI Sequence Read Archive database under accession code PRJNA922232. The processed sequencing data generated in this study are provided in the Source Data file. Source data are provided with this paper.

## Source data

Source data

## Code availability

Jupyter Notebook Python files associated with processing sequencing data have been deposited on GitHub [https://github.com/aos2/7_Plug_and_play_RNA_Detection].
