# Supplementary material for: A split ribozyme that links detection of a native RNA to orthogonal protein outputs
Source: Nat Commun. 2023 Feb 1;14:543. doi: 10.1038/s41467-023-36073-3 (PMC9892565; doi:10.1038/s41467-023-36073-3)
Supplement: Supplementary file 1 — Supplementary Information [file 41467_2023_36073_MOESM1_ESM.pdf]

Supplementary Information

A split ribozyme that links detection of a native RNA to orthogonal protein outputs

Lauren Gambill[1], August Staubus[2], Kim Wai Mo[2], Andrea Ameruoso[2], and James Chappell[1,2,3*]

1 – PhD Program in Systems, Synthetic, and Physical Biology, Rice University, Houston, TX, USA 77005
2 – Department of Biosciences, Rice University, Houston, TX, USA 77005
3 – Department of Bioengineering, Rice University, Houston, TX, USA 77005
*Corresponding author: Dr James Chappell, jc125@rice.edu

Table of contents

Tables	Title	Page
1	All plasmids used in this study	2 - 6
2	Example DNA plasmid sequence	6 - 7
3	RNA inputs used in this study	7 - 8
4	Guide sequences used in this study	8 - 10
5	Sequences of other biological parts used in this study	10 - 18
6	Strains used in this study	19
7	Species growth conditions	19
**Figures**		
1	Schematic of representative DNA plasmids used in this study	20
2	Identifying functional ribozyme insertion sites in sfGFP	21
3	Single cell fluorescence characterization of fluorescence-based splicing assay	22
4	Schematic of split-ribozyme library generation using transposon mutagenesis	23
5	Schematic of RNA inhibitor	24
6	Diversity of the split-ribozyme library	25
7	FACS of the split-ribozyme library results in enrichment at specific split sites	26
8	Identification of functional split ribozymes	27
9	Comparison of split variants identified through FACS-seq and individual colony screening	28
10	Three-dimensional structure shows functional ribozyme split sites in surface accessible regions	29
11	Two enriched split sites resulted in RENDR variants with low-fold activation	30
12	Reverse transcription quantitative PCR (RT-qPCR) of RENDR spliced output in the presence and absence of RNA input	31
13	Amplicon sequencing of RENDR spliced output	32
14	RENDR variants are specifically activated by their cognate RNA inputs	33
15	RNA guide length influences RENDR output levels and can be used as a predictive parameter	34
16	Dose-response curve of RENDR	35
17	RENDR variant using a flavin-containing monooxygenase (FMO) output produces visually discernable indigo levels upon detection of RNA	36
18	RENDR can couple the production of T7 RNAP to an RNA input	37
19	Cell culture turns blue in response to the presence of antibiotic resistance	38
20	Schematic of RENDR design and cloning	39
21	Gating strategy for flow cytometry experiments	40
**Notes**		
1	Designing RENDR for the detection of a new RNA input	41
2	Describing RENDR design principles with a thermodynamic model	42 - 43
3	NGS data analysis pipeline	44

Supplementary Table 1. All plasmids used in this study. CmR: chloramphenicol resistance gene, AmpR: ampicillin resistance gene, SpecR: spectinomycin resistance gene, p15A: origin of replication, ColE1: origin of replication, CDF: CloDF origin of replication, P: promoter, T: terminator, HP14: stability hairpin, RBS: ribosome binding site, P1_loop: P1 domain in *T. thermophila* ribozyme, IGS: internal guide sequence, sfGFP(X-YntZ) = sfGFP gene from amino acid X to amino acid Y at nucleotide position Z.

Plasmid	Figure	Name	Plasmid Architecture
pBW001	Na	Transposon	AmpR - ColE1 - Transposon (containing KanR)
pJEC101	All	Empty	CmR - p15A - TrrnB
pJEC102	All	Empty	AmpR - ColE1 - PJ23119 - TrrnB
pJEC103	All	Empty	SpecR - CDF - PJ23119 - TrrnB
pJEC752	F1	GFP	SpecR - CDF - PJ23119 - HP14 - RBS_01 - sfGFP(1-239) - TrrnB
pJEC753	F1	Ribozyme	SpecR - CDF - PJ23119 - HP14 - RBS_01 - sfGFP(1-66nt1) - P1_loop_WT - IGS_01 - Ribozyme - sfGFP(66nt2-239) - TrrnB
pJEC754	F1	Catalytically dead ribozyme	SpecR - CDF - PJ23119 - HP14 - RBS_01 - sfGFP(1-66nt1) - P1_loop_WT - IGS_01 - G264A dRibozyme - sfGFP(66nt2-239) - TrrnB
pJEC757	F2	Split ribozyme with toehold sequence	SpecR - CDF - PJ23119 - HP14 - RBS_01 - sfGFP(1-66nt1) - P1_loop_01 - Guide_01 - T500 - PJ23119 - Guide_02 - P1_loop_03 - IGS_01 - Ribozyme - sfGFP(66nt2-239) - T500
pJEC758	F2	AHL-inducible RNA inhibitor	AmpR - ColE1 - PTet - RBS_02 - LuxR - PLuxR - RNA inhibitor - T500
pJEC759	F2, SI 12, SI 15	RENDR split 15 nt into ribozyme (guide/input pair 2)	SpecR - CDF - PJ23119 - HP14 - RBS_01 - sfGFP(1-66nt1) - P1_loop_01 - Stem_01 - Guide_03 - T500 - PJ23119 - Guide_04 - Stem_02 - P1_loop_03 - IGS - Ribozyme - sfGFP(66nt2-239) - T500
pJEC760	F2, SI 11	RENDR split 26 nt into ribozyme	SpecR - CDF - TL3S2P55 - mCherry2 - RBS_02 - PJ23115 - PJ23119 - HP14 - RBS_01 - sfGFP(1-66nt1) - P1_loop_WT - Stem_01 - Guide_03 - T500 - PJ23119 - Guide_04 - Stem_02 - IGS_01 - Ribozyme - sfGFP(66nt2-239) - T500
pJEC761	F2	RENDR split 153 nt into ribozyme	SpecR - CDF - TL3S2P55 - mCherry2 - RBS_02 - PJ23115 - PJ23119 - HP14 - RBS_01 - sfGFP(1-66nt1) - P1_loop_WT - IGS_01 - Ribozyme(1-121) - Stem_01 - Guide_03 - T500 - PJ23119 - Guide_04 - Stem_02 - Ribozyme(122-388) - sfGFP(66nt2-239) - T500
pJEC762	F2	RENDR split 252 nt into ribozyme	SpecR - CDF - TL3S2P55 - mCherry2 - RBS_02 - PJ23115 - PJ23119 - HP14 - RBS_01 - sfGFP(1-66nt1) - P1_loop_WT - IGS_01 - Ribozyme(1-220) - Stem_01 - Guide_03 - T500 - PJ23119 - Guide_04 - Stem_02 - Ribozyme(221-388) - sfGFP(66nt2-239) - T500
pJEC763	F2, SI 11	RENDR split 327 nt into ribozyme	SpecR - CDF - TL3S2P55 - mCherry2 - RBS_02 - PJ23115 - PJ23119 - HP14 - RBS_01 - sfGFP(1-66nt1) - P1_loop_WT - IGS_01 - Ribozyme(1-295) - Stem_01 - Guide_03 - T500 - PJ23119 - Guide_04 -

			Stem_02 - Ribozyme(296-388) - sfGFP(66nt2-239) - T500
pJEC764	F2	RENDR split 347 nt into ribozyme	SpecR - CDF - TL3S2P55 - mCherry2 - RBS_02 - PJ23115 - PJ23119 - HP14 - RBS_01 - sfGFP(1-66nt1) - P1_loop_WT - IGS_01 - Ribozyme(1-315) - Stem_01 - Guide_03 - T500 - PJ23119 - Guide_04 - Stem_02 - Ribozyme(316-388) - sfGFP(66nt2-239) - T500
pJEC765	F2	RENDR split 369 nt into ribozyme	SpecR - CDF - TL3S2P55 - mCherry2 - RBS_02 - PJ23115 - PJ23119 - HP14 - RBS_01 - sfGFP(1-66nt1) - P1_loop_WT - IGS_01 - Ribozyme(1-337) - Stem_01 - Guide_03 - T500 - PJ23119 - Guide_04 - Stem_02 - Ribozyme(338-388) - sfGFP(66nt2-239) - T500
pJEC766	F2	RENDR split 402 nt into ribozyme	SpecR - CDF - TL3S2P55 - mCherry2 - RBS_02 - PJ23115 - PJ23119 - HP14 - RBS_01 - sfGFP(1-66nt1) - P1_loop_WT - IGS_01 - Ribozyme(1-370) - Stem_01 - Guide_03 - T500 - PJ23119 - Guide_04 - Stem_02 - Ribozyme(371-388) - sfGFP(66nt2-239) - T500
pJEC767	F2	Constitutive RNA input	AmpR - ColE1 - PJ23119 - Input_01 - T500
pJEC768	F3, SI15	RENDR (non-interacting guide/input pair)	SpecR - CDF - PJ23119 - HP14 - RBS_01 - sfGFP(1-66nt1) - P1_loop_01 - Guide_05 - T500 - PJ23119 - Guide_06 - P1_loop_03 - IGS_01 - Ribozyme - sfGFP(66nt2-239) - T500
pJEC769	S15	RENDR (guide/input pair 1)	SpecR - CDF - PJ23119 - HP14 - RBS_01 - sfGFP(1-66nt1) - P1_loop_01 - Guide_07 - T500 - PJ23119 - Guide_08 - P1_loop_03 - IGS_01 - Ribozyme - sfGFP(66nt2-239) - T500
pJEC770	S15	RENDR (guide/input pair 3)	SpecR - CDF - PJ23119 - HP14 - RBS_01 - sfGFP(1-66nt1) - P1_loop_01 - Guide_09 - T500 - PJ23119 - Guide_10 - P1_loop_03 - IGS_01 - Ribozyme - sfGFP(66nt2-239) - T500
pJEC776	SI 15	RNA input (guide/input pair 3)	AmpR - ColE1 - TTonB - araC - PBad - Input_04 - T500
pJEC779	F4	Modular RENDR-GFP	SpecR - CDF - PJ23119 - HP14 - RBS_01 - P1_helix_01- P1_loop_01 - Stem_01 - Guide_15 - T500 - PJ23119 - Guide_16 - Stem_02 - P1_loop_03 - IGS_01 - Ribozyme - sfGFP - TrrnB
pJEC780	F4	Constitutive synthetic RNA input	AmpR - ColE1 - PJ23119 - Input_07 - T500
pJEC781	F4, SI 17	Modular RENDR-FMO	SpecR - CDF - PJ23119 - HP14 - RBS_01 - P1_helix_01- P1_loop_01 - Stem_01 - Guide_15 - T500 - PJ23119 - Guide_16 - Stem_02 - P1_loop_03 - IGS_01 - Ribozyme - FMO - TrrnB
pJEC782	F4	Modular RENDR-MHT	SpecR - CDF - PJ23119 - HP14 - RBS_01 - P1_helix_01 - P1_loop_01 - Stem_01 - Guide_15 - T500 - PJ23119 - Guide_16 - Stem_02 - P1_loop_03 - IGS_01 - Ribozyme - MHT - TrrnB
pJEC783	F4	Modular RENDR-dCas9	SpecR - CDF - PJ23119 - HP14 - RBS_01 - P1_helix_01 - P1_loop_01 - Stem_01 - Guide_15 - T500 - PJ23119 - Guide_16 - Stem_02 - P1_loop_03 - IGS_01 - Ribozyme - dCas9 - TrrnB
pJEC784	F4	sgRNA	CmR - p15A - PJ23119 - sgRNA_01 - TrrnB

pJEC785	F5	KanR sensing RENDR-GFP	SpecR - CDF - PJ23119 - HP14 - RBS_01 - P1_helix_01 - P1_loop_01 - Stem_01 - Guide_17 - T500 - PJ23119 - Guide_18 - Stem_02 - P1_loop_03 - IGS_01 - Ribozyme - sfGFP - TrrnB
pJEC786	F5, F6, SI 18, SI 19	KanR RNA input	AmpR - ColE1 - PJ23119 - RBS_01 - Input_08 - T500
pJEC787	F6, SI 19	KanR sensing RENDR-FMO	SpecR - CDF - PJ23119 - HP14 - RBS_01 - P1_helix_01 - P1_loop_01 - Stem_01 - Guide_17 - T500 - PJ23119 - Guide_18 - Stem_02 - P1_loop_03 - IGS_01 - Ribozyme - FMO - TrrnB
pJEC788	SI 2	Ribozyme split at E172	SpecR - CDF - PJ23119 - HP14 - RBS_01 - sfGFP(1-172nt3) - P1_helix_02 - P1_loop_WT - IGS_02 - Ribozyme - sfGFP(173nt1-239) - TrrnB
pJEC789	SI 2	Catalytically dead ribozyme split at E172	SpecR - CDF - PJ23119 - HP14 - RBS_01 - sfGFP(1-172nt3) - P1_helix_02 - P1_loop_WT - IGS_02 - dRibozyme - sfGFP(173nt1-239) - TrrnB
pJEC790	SI 2	Ribozyme split at D21	SpecR - CDF - PJ23119 - HP14 - RBS_01 - sfGFP(1-21nt3) - P1_loop_WT - IGS_03 - Ribozyme - sfGFP(22nt1-239) - TrrnB
pJEC791	SI 2	Catalytically dead ribozyme split at D21	SpecR - CDF - PJ23119 - HP14 - RBS_01 - sfGFP(1-21nt3) - P1_loop_WT - IGS_03 - dRibozyme - sfGFP(22nt1-239) - TrrnB
pJEC792	SI 17	Ribozyme with FMO output	SpecR - CDF - PJ23119 - HP14 - RBS_01 - P1_helix_01- P1_loop_WT - IGS_01 - Ribozyme - FMO - TrrnB
pJEC793	SI 18	KanR-sensing RENDR with T7 RNAP	SpecR - CDF - PJ23119 - RBS_03 - T7RNAP(1-66) - P1_loop_01 - Stem_01 - Guide_17 - T500 - PJ23119 - Guide_18 - Stem_02 - P1_loop_03 – IGS_04 - Ribozyme – T7RNAP(67-884) - T500
pJEC794	SI 18	T7-inducible GFP	CmR - p15A - PT7 - HP14 - RBS_01 - sfGFP - TrrnB
pJEC847	N/A	RENDR staging vector	SpecR - CDF - PJ23119 - HP14 - RBS_04 - sfGFP_1(1-66nt1) - P1_loop_01 - Insert_01 - T500 - PJ23119 - Insert_02 - P1_loop_03 - IGS_01 - Ribozyme - sfGFP(66nt2-239) - T500
pJEC866	SI 16	RNA input (guide/input pair 6)	AmpR - ColE1 - AraC - pBad - Input_07 - t500
pJEC867	SI 16	RENDR (guide/input pair 6)	SpecR - CDF - PJ23119 - HP14 - RBS_04 - sfGFP_1(1-66nt1) - P1_loop_01 - Stem_01 - Guide_15 - T500 - PJ23119 - Guide_16 - Stem_02 - P1_loop_03 - IGS_01 - Ribozyme - sfGFP(66nt2-239) - T500
pJEC910	SI 16	Arabinose-inducible GFP	AmpR - ColE1 - AraC - pBad - RBS_01 - sfGFP - T500
pJEC964	F3, SI 15	RNA input RFP (guide/input pair 7,8,9,10,11)	AmpR - ColE1 - AraC - pBad - Input_09 - T500
pJEC949	F3	RENDR RFP (guide/input pair 7)	SpecR - CDF - PJ23119 - HP14 - RBS_04 - sfGFP_1(1-66nt1) - P1_loop_01 - Stem_01 - Guide_19 - T500 - PJ23119 - Guide_20 - Stem_02 - P1_loop_03 - IGS_01 - Ribozyme - sfGFP(66nt2-239) - T500
pJEC950	F3	RENDR RFP (guide/input pair 8)	SpecR - CDF - PJ23119 - HP14 - RBS_04 - sfGFP_1(1-66nt1) - P1_loop_01 - Stem_01 - Guide_21 - T500 - PJ23119 - Guide_22 - Stem_02 -

			P1_loop_03 - IGS_01 - Ribozyme - sfGFP(66nt2-239) - T500
pJEC951	F3	RENDR RFP (guide/input pair 9)	SpecR - CDF - PJ23119 - HP14 - RBS_04 - sfGFP_1(1-66nt1) - P1_loop_01 - Stem_01 - Guide_23 - T500 - PJ23119 - Guide_24 - Stem_02 - P1_loop_03 - IGS_01 - Ribozyme - sfGFP(66nt2-239) - T500
pJEC952	F3	RENDR RFP (guide/input pair 10)	SpecR - CDF - PJ23119 - HP14 - RBS_04 - sfGFP_1(1-66nt1) - P1_loop_01 - Stem_01 - Guide_25 - T500 - PJ23119 - Guide_26 - Stem_02 - P1_loop_03 - IGS_01 - Ribozyme - sfGFP(66nt2-239) - T500
pJEC953	F3	RENDR RFP (guide/input pair 11)	SpecR - CDF - PJ23119 - HP14 - RBS_04 - sfGFP_1(1-66nt1) - P1_loop_01 - Stem_01 - Guide_27 - T500 - PJ23119 - Guide_28 - Stem_02 - P1_loop_03 - IGS_01 - Ribozyme - sfGFP(66nt2-239) - T500
pJEC954	F3, SI 14	RENDR RFPv2 (guide/input pair 12)	SpecR - CDF - PJ23119 - HP14 - RBS_04 - sfGFP_1(1-66nt1) - P1_loop_01 - Stem_01 - Guide_29 - T500 - PJ23119 - Guide_30 - Stem_02 - P1_loop_03 - IGS_01 - Ribozyme - sfGFP(66nt2-239) - T500
pJEC955	F3	CatDev1	SpecR - CDF - PJ23119 - HP14 - RBS_04 - sfGFP_1(1-66nt1) - P1_loop_01 - Stem_01 - Guide_31 - T500 - PJ23119 - Guide_32 - Stem_02 - P1_loop_03 - IGS_01 - Ribozyme - sfGFP(66nt2-239) - T500
pJEC956	F3, SI 14	CatDev2	SpecR - CDF - PJ23119 - HP14 - RBS_04 - sfGFP_1(1-66nt1) - P1_loop_01 - Stem_01 - Guide_33 - T500 - PJ23119 - Guide_34 - Stem_02 - P1_loop_03 - IGS_01 - Ribozyme - sfGFP(66nt2-239) - T500
pJEC957	F3, SI 14	TetAv1	SpecR - CDF - PJ23119 - HP14 - RBS_04 - sfGFP_1(1-66nt1) - P1_loop_01 - Stem_01 - Guide_35 - T500 - PJ23119 - Guide_36 - Stem_02 - P1_loop_03 - IGS_01 - Ribozyme - sfGFP(66nt2-239) - T500
pJEC958	F3	TetAv2	SpecR - CDF - PJ23119 - HP14 - RBS_04 - sfGFP_1(1-66nt1) - P1_loop_01 - Stem_01 - Guide_37 - T500 - PJ23119 - Guide_38 - Stem_02 - P1_loop_03 - IGS_01 - Ribozyme - sfGFP(66nt2-239) - T500
pJEC959	S15	RENDR 11.1% mismatch	SpecR - CDF - PJ23119 - HP14 - RBS_04 - sfGFP_1(1-66nt1) - P1_loop_01 - Stem_01 - Guide_39 - T500 - PJ23119 - Guide_40 - Stem_02 - P1_loop_03 - IGS_01 - Ribozyme - sfGFP(66nt2-239) - T500
pJEC960	S15	RENDR 16.7% mismatch	SpecR - CDF - PJ23119 - HP14 - RBS_04 - sfGFP_1(1-66nt1) - P1_loop_01 - Stem_01 - Guide_41 - T500 - PJ23119 - Guide_42 - Stem_02 - P1_loop_03 - IGS_01 - Ribozyme - sfGFP(66nt2-239) - T500
pJEC961	S15	RENDR 66% mismatch	SpecR - CDF - PJ23119 - HP14 - RBS_04 - sfGFP_1(1-66nt1) - P1_loop_01 - Stem_01 - Guide_47 - T500 - PJ23119 - Guide_48 - Stem_02 -

			P1_loop_03 - IGS_01 - Ribozyme - sfGFP(66nt2-239) - T500
pJEC962	S15	RENDR 100% mismatch	SpecR - CDF - PJ23119 - HP14 - RBS_04 - sfGFP_1(1-66nt1) - P1_loop_01 - Stem_01 - Guide_49 - T500 - PJ23119 - Guide_50 - Stem_02 - P1_loop_03 - IGS_01 - Ribozyme - sfGFP(66nt2-239) - T500
pJEC975	F3, S14, S15	Constitutive RFP	CmR - P15A - PJ23119 - RBS_05 - RFP - Tdbl
pJEC976	F3, S14	Constitutive CatDe	CmR - P15A - PJ23119 - RBS_05 - Catechol 2,3-diooxygenase - Tdbl
pJEC977	F3, S14	Constitutive TetA	CmR - P15A - PJ23119 - RBS_05 - TetA - Tdbl
pJEC978	S15	RENDR 25% mismatch	SpecR - CDF - PJ23119 - HP14 - RBS_04 - sfGFP_1(1-66nt1) - P1_loop_01 - Stem_01 - Guide_43 - T500 - PJ23119 - Guide_44 - Stem_02 - P1_loop_03 - IGS_01 - Ribozyme - sfGFP(66nt2-239) - T500
pJEC979	S15	RENDR 33% mismatch	SpecR - CDF - PJ23119 - HP14 - RBS_04 - sfGFP_1(1-66nt1) - P1_loop_01 - Stem_01 - Guide_45 - T500 - PJ23119 - Guide_46 - Stem_02 - P1_loop_03 - IGS_01 - Ribozyme - sfGFP(66nt2-239) - T500

Supplementary Table 2. Example DNA plasmid sequence.

Name	DNA sequence
Example of cis-acting ribozyme inserted within sfGFP, pJEC753 [SpecR - CDF - PJ23119 - HP14 - RBS_01 - sfGFP(1-66nt1) - P1_loop_WT - IGS_01 - Ribozyme - sfGFP(66nt2-239) - TrrnB]	TTATTTGCCGACTACCTTGGTGATCTCGCCTTTCACGTAGTGGACAAATTCTTCCAA<br>CTGATCTGCGCGCGAGGCCAAGCGATCTTCTTCTTGTCCAAGATAAGCCTGTCTAG<br>CTTCAAGTATGACGGGCTGATACTGGGCCGGCAGGCGCTCCATTGCCCAGTCGG<br>CAGCGACATCCTTCGGCGCGATTTTGCCGGTTACTGCGCTGTACCAAATGCGGGA<br>CAACGTAAGCACTACATTTCGCTCATCGCCAGCCCAGTCGGGCGGCGAGTTCCAT<br>AGCGTTAAGGTTTCATTTAGCGCCTCAAATAGATCCTGTTCAGGAACCGGATCAAA<br>GAGTTCCTCCGCCGCTGGACCTACCAAGGCAACGCTATGTTCTCTTGCTTTTGTCA<br>GCAAGATAGCCAGATCAATGTCGATCGTGGCTGGCTCGAAGATACCTGCAAGAAT<br>GTCATTGCGCTGCCATTCTCCAAATTGCAGTTCGCGCTTAGCTGGATAACGCCACG<br>GAATGATGTCGTCGTGCACAACAATGGTGACTTCTACAGCGCGGAGAATCTCGCT<br>CTCTCCAGGGGAAGCCGAAGTTTCCAAAAGGTCGTTGATCAAAGCTCGCCGCGTT<br>GTTTCATCAAGCCTTACGGTCACCGTAACCAGCAAATCAATATCACTGTGTGGCTT<br>CAGGCCGCCATCCACTGCGGAGCCGTACAAATGTACGGCCAGCAACGTCGGTTC<br>GAGATGGCGCTCGATGACGCCAACTACCTCTGATAGTTGAGTCGATACTTCGGCG<br>ATCACCGCTTCCCTCATACTCTTCCTTTTTCAATATTATTGAAGCATTTATCAGGGTT<br>ATTGTCTCATGAGCGGATACATATTTGAATGTATTTAGAAAAATAAACAAATAGCTA<br>GCTCACTCGGTCGCTACGCTCCGGGCGTGAGACTGCGGCGGGCGCTGCGGACAC<br>ATACAAAGTTACCCACAGATTCCGTGGATAAGCAGGGGACTAACATGTGAGGCAA<br>AACAGCAGGGCCGCGCCGGTGGCGTTTTTCCATAGGCTCCGCCCTCCTGCCAGA<br>GTTCACATAAACAGACGCTTTTCCGGTGCATCTGTGGGAGCCGTGAGGCTCAACC<br>ATGAATCTGACAGTACGGGCGAAACCCGACAGGACTTAAAGATCCCCACCGTTTC<br>CGGCGGGTCGCTCCCTCTTGCGCTCTCCTGTTCCGACCCTGCCGTTTACCGGATA<br>CCTGTTCCGCCTTTCTCCCTTACGGGAAGTGTGGCGCTTTCTCATAGCTCACACAC<br>TGGTATCTCGGCTCGGTGTAGGTCGTTCGCTCCAAGCTGGGCTGTAAGCAAGAAC<br>TCCCCGTTCAGCCCGACTGCTGCGCCTTATCCGGTAACTGTTCACTTGAGTCCAAC<br>CCGGAAAAGCACGGTAAAACGCCACTGGCAGCAGCCATTGGTAACTGGGAGTTCG<br>CAGAGGATTTGTTTAGCTAAACACGCGGTTGCTCTTGAAGTGTGCGCCAAAGTCCG

	GCTACACTGGAAGGACAGATTTGGTTGCTGTGCTCTGCGAAAGCCAGTTACCACG<br>GTTAAGCAGTTCCCCAACTGACTTAACCTTCGATCAAACCACCTCCCCAGGTGGTT<br>TTTTCGTTTACAGGGCAAAAGATTACGCGCAGAAAAAAAGGATCTCAAGAAGATCC<br>TTTGATCTTTTCTACTGAACCGCTCTAGATTTCAGTGCAATTTATCTCTTCAAATGTA<br>GCACCTGAAGTCAGCCCCATACGATATAAGTTGTAATTCTCATGTTAGTCATGCCC<br>CGCGCCCACCGGAAGGAGCTGACTGGGTTGAAGGCTCTCAAGGGCATCGGTCGA<br>GATCCCGGTGCCTAATGAGTGAGCTAACTTACACCCGCAGTTCCAGTACGGTTCC<br>ATTAATTGCGTTGCGCTGCTTCCGGCTGAATTCTAAAGATCTTTGACAGCTAGCTC<br>AGTCCTAGGTATAATACTAGTACGTCGACTCTCGAGTGAGATTGTTGACGGTACCG<br>TATTTTGGATCTAGGAGGAAGGATCTATGAGCAAAGGAGAAGAACTTTTCACTGGA<br>GTTGTCCCAATTCTTGTTGAATTAGATGGTGATGTTAATGGGCACAAATTTTCTGTC<br>CGTGGAGAGGGTGAAGGTGATGCTACAAACGGAAAACTCACCCTTAAATTTATTTG<br>CACTACTGGAAAACTACCTGTTCCGTGGCCAACACTTGTCACTACTCTGACCTAAA<br>TAGCAATATTTACCTTTGGGTCAAAAAGTTATCAGGCATGCACCTGGTAGCTAGTCT<br>TTAAACCAATAGATTGCATCGGTTTAAAAGGCAAGACCGTCAAATTGCGGGAAAGG<br>GGTCAACAGCCGTTCAGTACCAAGTCTCAGGGGAAACTTTGAGATGGCCTTGCAA<br>AGGGTATGGTAATAAGCTGACGGACATGGTCCTAACCACGCAGCCAAGTCCTAAG<br>TCAACAGATCTTCTGTTGATATGGATGCAGTTCACAGACTAAATGTCGGTCGGGGA<br>AGATGTATTCTTCTCATAAGATATAGTCGGACCTCTCCTTAATGGGAGCTAGCGGA<br>TGAAGTGATGCAACACTGGAGCCGCTGGGAACTAATTTGTATGCGAAAGTATATTG<br>ATTAGTTTTGGAGTACTCGATGGTGTTCAATGCTTTTCCCGTTATCCGGATCACATG<br>AAACGGCATGACTTTTTCAAGAGTGCCATGCCCGAAGGTTATGTACAGGAACGCA<br>CTATATCTTTCAAAGATGACGGGACCTACAAGACGCGTGCTGAAGTCAAGTTTGAA<br>GGTGATACCCTTGTTAATCGTATCGAGTTAAAGGGTATTGATTTTAAAGAAGATGGA<br>AACATTCTTGGACACAAACTCGAGTACAACTTTAACTCACACAATGTATACATCACG<br>GCAGACAAACAAAAGAATGGAATCAAAGCTAACTTCAAAATTCGCCACAACGTTGA<br>AGATGGTTCCGTTCAACTAGCAGACCATTATCAACAAAATACTCCAATTGGCGATG<br>GCCCTGTCCTTTTACCAGACAACCATTACCTGTCGACACAATCTGTCCTTTCGAAA<br>GATCCCAACGAAAAGCGTGACCACATGGTCCTTCTTGAGTTTGTAACTGCTGCTGG<br>GATTACACATGGCATGGATGAGCTCTACAAATAAGGATCTGAAGCTTGGGCCCGA<br>ACAAAAACTCATCTCAGAAGAGGATCTGAATAGCGCCGTCGACCATCATCATCATC<br>ATCATTGAGTTTAAACGGTCTCCAGCTTGGCTGTTTTGGCGGATGAGAGAAGATTT<br>TCAGCCTGATACAGATTAAATCAGAACGCAGAAGCGGTCTGATAAAACAGAATTTG<br>CCTGGCGGCAGTAGCGCGGTGGTCCCACCTGACCCCATGCCGAACTCAGAAGTG<br>AAACGCCGTAGCGCCGATGGTAGTGTGGGGTCTCCCCATGCGAGAGTAGGGAAC<br>TGCCAGGCATCAAATAAAACGAAAGGCTCAGTCGAAAGACTGGGCCTTTCGTTTTA<br>TCTGTTGTTTGTCGGTGAACT

Supplementary Table 3. RNA inputs used in this study.

Name	Length (nt)	Sequence
Input_01	169	TTTTACCAAAAAGAAATTCGGACAAGCTTATTGCTCGTAAAAAAGACTGGG<br>ATCCAAAAAAAATATGGTGGTTTTGATAGTCCAACGGTAGCTTATTCAGTCCT<br>AGTGGTTGCTAAGGTGGAAAAAGGGAAATCGAAGAAGTTAAAATCCGTTAA<br>AGAGTTACTAGGGAT
Input_04	251	GAAAACAGAAGTACAGACAGGCGGATTCTCCAAGGAGTCAATTTTACCAAA<br>AAGAAATTCGGACAAGCTTATTGCTCGTAAAAAAGACTGGGATCCAAAAAA<br>ATATGGTGGTTTTGATAGTCCAACGGTAGCTTATTCAGTCCTAGTGGTTGC<br>TAAGGTGGAAAAAGGGAAATCGAAGAAGTTAAAATCCGTTAAAGAGTTACT<br>AGGGATCACAATTATGGAAAGAAGTTCCTTTGAAAAAAATCCGATTG
Input_07	169	CCACTCAACGATGTGGGGACGCCGTTGCAACTTCGAGGACCTAATGTGAC<br>CGACCTAGATTCGTCATTGTGGGCAGAATGAAGTATTGGCAGACATTGAGT

		GCCGAACAAGACCTGACCTAACGGTAAGAGAGTCTCATAATACGTCCGGCCTCTTGCGCAGGtTATAT
Input_08	816	ATGAGCCATATTCAACGGGAAACGTCTTGCTCCAGGCCGCGATTAAATTCCAACATGGATGCTGATTTATATGGGTATAAATGGGCTCGCGATAATGTCGGGCAATCAGGTGCGACAATCTATCGATTGTATGGGAAGCCCGATGCGCCAGAGTTGTTTCTGAAACATGGCAAAGGTAGCGTTGCCAATGATGTTACAGATGAGATGGTCAGACTAAACTGGCTGACGGAATTTATGCCTCTTCCGACCATCAAGCATTTTATCCGTACTCCTGATGATGCATGGTTACTCACCACTGCGATCCCCGGGAAAACAGCATTCCAGGTATTAGAAGAATATCCTGATTCAGGTGAAAATATTGTTGATGCGCTGGCAGTGTTCCTGCGCCGGTTGCATTCGATTCCTGTTTGTAATTGTCCTTTTAACAGCGATCGCGTATTTCGTCTCGCTCAGGCGCAATCACGAATGAATAACGGTTTGGTTGATGCGAGTGATTTTGATGACGAGCGTAATGGCTGGCCTGTTGAACAAGTCTGGAAAGAAATGCATAAGCTTTTGCCATTCTCACCGGATTCAGTCGTCACTCATGGTGATTTCTCACTTGATAACCTTATTTTTGACGAGGGGAAATTAATAGGTTGTATTGATGTTGGACGAGTCGGAATCGCAGACCGATACCAGGATCTTGCCATCCTATGGAACTGCCTCGGTGAGTTTTCTCCTTCATTACAGAAACGGCTTTTTCAAAAATATGGTATTGATAATCCTGATATGAATAAATTGCAGTTTCATTTGATGCTCGATGAGTTTTTCTAA
Input_09	655	CGAAGACGTTATCAAAGAGTTCATGCGTTTCAAAGTTCGTATGGAAGGTTCCGTTAACGGTCACGAGTTCGAAATCGAAGGTGAAGGTGAAGGTCGTCCGTACGAAGGTACCCAGACCGCTAAACTGAAAGTTACCAAAGGTGGTCCGCTGCCGTTCGCTTGGGACATCCTGTCCCCGCAGTTCCAGTACGGTTCCAAAGCTTACGTTAAACACCCGGCTGACATCCCGGACTACCTGAAACTGTCCTTCCCGGAAGGTTTCAAATGGGAACGTGTTATGAACTTCGAAGACGGTGGTGTTGTTACCGTTACCCAGGACTCCTCCCTGCAAGACGGTGAGTTCATCTACAAAGTTAAACTGCGTGGTACCAACTTCCCGTCCGACGGTCCGGTTATGCAGAAAAAAACCATGGGTTGGGAAGCTTCCACCGAACGTATGTACCCGGAAGACGGTGCTCTGAAAGGTGAAATCAAAATGCGTCTGAAACTGAAAGACGGTGGTCACTACGACGCTGAAGTTAAAACCACCTACATGGCTAAAAAACCGGTTCAGCTGCCGGGTGCTTACAAAACCGACATCAAACTGGACATCACCTCCCACAACGAAGACTACACCATCGTTGAACAGTACGAACGTGCTGAAGGTCGTCACTCC

Supplementary Table 4. Guide sequences used in this study.

Name	Length (nt)	Sequence
Guide_01	164	CGTTGACGAATCTTGGAGCTCCCGCTGCTTTTAAATATTTTGATACAACAATTGATCGTAAACGATATACGTCTACAAAAGAAGTTTTAGATGCCACTCTTATCCATCAATCCATCACTGGTCTTTATGAAACACGCATTGATTTGAGTCAGCTAGGAGGTGAC
Guide_02	184	ACCAATACACGAACAAGCAGGTCACCTCCTAGCTGACTCAAATCAATGCGTGTTTCATAAAGACCAGTGATGGATTGATGGATAAGAGTGGCATCTAAAACTTCTTTTGTAGACGTATATCGTTTACGATCAATTGTTGTATCAAAATATTTAAAAGCAGCGGGAGCTCCAAGATTCGTCAACG
Guide_03	82	GGACTATCAAAACCACCATATTTTTTTGGATCCCAGTCTTTTTTACGAGCAATAAGCTTGTCCGAATTTCTTTTTGGTAAAA
Guide_04	82	ATCCCTAGTAACTCTTTAACGGATTTTAACTTCTTCGATTTCCCTTTTTCCACCTTAGCAACCACTAGGACTGAATAAGCTA
Guide_05	20	ATCACACTCCCACTCCACCT
Guide_06	20	CACACGCACATTCTCATACC

Guide_07	41	GGACTATCAAAACCACCATATTTTTTGGATCCCAGTCTTT
Guide_08	41	CCCTTTTTCCACCTTAGCAACCACTAGGACTGAATAAGCTA
Guide_09	123	GGACTATCAAAACCACCATATTTTTTGGATCCCAGTCTTTTTTACGAGCAATAAGCTTGTCCGAATTTCTTTTTGGTAAAATTGACTCCTTGGAGAATCCGCCTGTCTGTACTTCTGTTTTC
Guide_10	123	CAATCGGATTTTTTTCAAAGGAACTTCTTTCCATAATTGTGATCCCTAGTAACTCTTTAACGGATTTTAACTTCTTCGATTTCCCTTTTTCCACCTTAGCAACCACTAGGACTGAATAAGCTA
Guide_15	82	TTCATTCTGCCCACAATGACGAATCTAGGTCGGTCACATTAGGTCCTCGAAGTTGCAACGGCGTCCCCACATCGTTGAGTGG
Guide_16	82	ATATAACCTGCGCAAGAGGCCGGACGTATTATGAGACTCTCTTACCGTTAGGTCAGGTCTTGTTCGGCACTCAATGTCTGCC
Guide_17	82	GAGCCCATTTATACCCATATAAATCAGCATCCATGTTGGAATTTAATCGCGGCCTGGAGCAAGACGTTTCCCGTTGAATATG
Guide_18	82	CTTTGCCATGTTTCAGAAACAACTCTGGCGCATCGGGCTTCCCATACAATCGATAGATTGTCGCACCTGATTGCCCGACATT
Guide_19	41	GGAGGAGTCCTGGGTAACGGTAACAACACCACCGTCTTCGA
Guide_20	41	TGGTACCACGCAGTTTAACTTTGTAGATGAACTCACCGTCT
Guide_21	82	GGAGGAGTCCTGGGTAACGGTAACAACACCACCGTCTTCGAAGTTCATAACACGTTCCCATTTGAAACCTTCCGGGAAGGAC
Guide_22	82	CATGGTTTTTTTCTGCATAACCGGACCGTCGGACGGGAAGTTGGTACCACGCAGTTTAACTTTGTAGATGAACTCACCGTCT
Guide_23	123	GGAGGAGTCCTGGGTAACGGTAACAACACCACCGTCTTCGAAGTTCATAACACGTTCCCATTTGAAACCTTCCGGGAAGGACAGTTTCAGGTAGTCCGGGATGTCAGCCGGGTGTTTAACGTA
Guide_24	123	CCGTCTTCCGGGTACATACGTTCGGTGGAAGCTTCCCAACCCATGGTTTTTTTCTGCATAACCGGACCGTCGGACGGGAAGTTGGTACCACGCAGTTTAACTTTGTAGATGAACTCACCGTCT
Guide_25	164	GGAGGAGTCCTGGGTAACGGTAACAACACCACCGTCTTCGAAGTTCATAACACGTTCCCATTTGAAACCTTCCGGGAAGGACAGTTTCAGGTAGTCCGGGATGTCAGCCGGGTGTTTAACGTAAGCTTTGGAACCGTACTGGAACTGCGGGGACAGGATGTCCC
Guide_26	164	CTTTCAGTTTCAGACGCATTTTGATTTCACCTTTCAGAGCACCGTCTTCCGGGTACATACGTTCGGTGGAAGCTTCCCAACCCATGGTTTTTTTCTGCATAACCGGACCGTCGGACGGGAAGTTGGTACCACGCAGTTTAACTTTGTAGATGAACTCACCGTCT
Guide_27	331	GGAGGAGTCCTGGGTAACGGTAACAACACCACCGTCTTCGAAGTTCATAACACGTTCCCATTTGAAACCTTCCGGGAAGGACAGTTTCAGGTAGTCCGGGATGTCAGCCGGGTGTTTAACGTAAGCTTTGGAACCGTACTGGAACTGCGGGGACAGGATGTCCCAAGCGAACGGCAGCGGACCACCTTTGGTAACTTTCAGTTTAGCGGTCTGGGTACCTTCGTACGGACGACCTTCACCTTCACCTTCGATTTCGAACTCGTGACCGTTAACGGAACCTTCCATACGAACTTTGAAACGCATGAACTCTTTGATAACGTCTTCG
Guide_28	331	GGAGTGACGACCTTCAGCACGTTCGTACTGTTCAACGATGGTGTAGTCTTCGTTGTGGGAGGTGATGTCCAGTTTGATGTCGGTTTTGTAAGCACCCGGCAGCTGAACCGGTTTTTTAGCCATGTAGGTGGTTTTAACTTCAGCGTCGTAGTGACCACCGTCTTTCAGTTTCAGACGCATTTTGATTTCACCTTTCAGAGCACCGTCTTCCGGGTACATACGTTCGGTGGAAGCTTCCCAACCCATGGTTTTTTTCTGCATAACCGGACCGTCGGACGGGAAGTTGGTACCACGCAGTTTAACTTTGTAGATGAACTCACCGTCT

Guide_29	82	GATGTCGGTTTTGTAAGCACCCGGCAGCTGAACCGGTTTTTTAGCCATGTAGGTGGTTTTAACTTCAGCGTCGTAGTGACCA
Guide_30	82	TTAAGCACCGGTGGAGTGACGACCTTCAGCACGTTCGTACTGTTCAACGATGGTGTAGTCTTCGTTGTGGGAGGTGATGTCC
Guide_31	82	ACAACCTTGAAACCCATAAAATCCATGCCCGGCTCGTCAGCCTCGCGTAGCACCAGGGAAAACTTATCCACTTCGGTCCAAG
Guide_32	82	CTGTTCAGTTCACCTGCGGGTAGCTGCTCAACGGCACAGCCATATGCCATCAGATCCCGCTCCAGTTGCCGGAGAGCATCCT
Guide_33	82	CGGTTTGTGGTCCGGGTAGTTGTAATCTCCCCCGCAGAACACTTCGTTGCGGTTACCGGACGGGTCGAAGAAGTAGATGGTC
Guide_34	82	TTAGGTCAGCACGGTCATGAATCGTTCGTTGAGAATGCGGTCGTGGTAAAAGATCGCCTTGCCCAGCTGGTCGGTGGTCCAG
Guide_35	82	GTACCGGCATAACCAAGCCTATGCCTACAGCATCCAGGGTGACGGTGCCGAGGATGACGATGAGCGCATTGTTAGATTTCAT
Guide_36	82	ATTGCATCAACGCATATAGCGCTAGCAGCACGCCATAGTGACTGGCGATGCTGTCGGAATGGACGATATCCCGCAAGAGGCC
Guide_37	82	CCCAGTAGTAGGTTGAGGCCGTTGAGCACCGCCGCCGCAAGGAATGGTGCATGCAAGGAGATGGCGCCCAACAGTCCCCCGG
Guide_38	82	GCCCACCGGAAGGAGCTGACTGGGTTGAAGGCTCTCAAGGGCATCGGTCGACGCTCTCCCTTATGCGACTCCTGCATTAGGA
Guide_39	82	CGACTATCATAACCACCAAATTTTTTTCGATCCCAGACTTTTTTAGGAGCAATATGCTTGTCCCAATTTCTTATTGGTAAAT
Guide_40	82	TTCCCTAGTTACTCTTTATCGGATTTTTACTTCTTCCATTTCCCTATTTCCACCATAGCAACCTCTAGGACTCAATAAGCTT
Guide_41	82	CGACTAACAAAAGCACCAAATTTTATTGGAACCCAGACTTTTATACGACCAATATGCTTGACCGAAATTCTTATTGGTTAAA
Guide_42	82	TTCCCTTGTAACACTTTATCGGATATTAACATCTTCCATTTCGCTTTTACCACCATAGCATCCACTTGGACTCAATAACCTA
Guide_43	82	CGACAATCTAAAGCACGATAATTTATTGCATCGCAGACTTATTTTCGACCAAAAAGGTTGACCGTATTACTTATTGCTAATA
Guide_44	82	TTCCGTAGAAACACTTAAACCGATATTATCTTGTTCCATTACCCATTTACCAGCTTTGCATCCAGTAGCACTCAATTAGCAA
Guide_45	82	CGAGTAACATAAGCAGCAAATATTATTCGAACCGAGACTATTATAGGACCATTATGCATGACCCAAATTGTTATTCGTTAAT
Guide_46	82	TTCGCTTGTTACACTATATCGCATATTTACATCATCCATATCGCTATTACCTCCATACCATCCTCTTGGTCTCAAAAACCTT
Guide_47	82	CCAGAAAGATTAGGAGGAATTAATAATCCAAGCGTGAGTAATAAAGCACGATAATCCAAGAGCCTAAATGATAATCCTTTAT
Guide_48	82	TACGGTTCTTTCAGTAAATGGCTTAATTTCAACAACCTTAACGGTAATAGCTGCAAACGATGCTGTTCGTGTCTAATACGTT
Guide_49	82	CCTGATAGTTTTGGTGGTATAAAAAAACCTAGGGTCAGAAAAAATGCTCGTTATTCGAACAGGCTTAAAGAAAAACCATTTT
Guide_50	82	TAGGGATCATTGAGAAATTGCCTAAAATTGAAGAAGCTAAAGGGAAAAAGGTGGAATCGTTGGTGATCCTGACTTATTCGAT

Supplementary Table 5. Sequences of other biological parts used in this study.

Part	DNA sequence (5’ to 3’)	Protein Sequence
IGS_01	GGGTCA	
IGS_02	GGAGGG	
IGS_03	GTCACC	
IGS_04	GTCCGC	

P1_helix_01	TGACCT
P1_helix_02	CCCTCT
RBS_01	AGGAGGAA
RBS_02	AAAGAGGAGAAA
RBS_03	AAAGAGGAGAAATACTAG
RBS_04	AGGAGGAAGGATCT
RBS_05	AGACGGACATCTAAGATTAAGGAGAGATTTT
HP14	ACGTCGACTCTCGAGTGAGATTGTTGACGGTACCGTATTTT
Toehold	ACCAATACACGAACAAGCAG
RNA inhibitor	CGTTGACGAATCTTGGAGCTCCCGCTGCTTTTAAATATTTTGATACAACAATTGATCGTAAACGATATACGTCTACAAAAGAAGTTTTAGATGCCACTCTTATCCATCAATCCATCACTGGTCTTTATGAAACACGCATTGATTTGAGTCAGCTAGGAGGTGACCTGCTTGTTCGTGTATTGGT
Insert_01	TGAGACGGACATGGCAACGTCTCA
Insert_02	TGAGACGCAGCATACTTCGTCTCA
Stem_01	GGATCA
Stem_02	TGATCC
P1_loop_WT	AAATAGCAATATTTACCTTT
P1_loop_01	AAATAGCAA
P1_loop_02	TATTTACCTTT
P1_loop_03	TAGTTACCTTT
PJ23119	TTGACAGCTAGCTCAGTCCTAGGTATAATACTAGT
PJ23115	TTTATAGCTAGCTCAGCCCTTGGTACAATGCTAGC
Pbad	GCCGTCACTGCGTCTTTTACTGGCTCTTCTCGCTAACCAAACCGGTAACCCCGCTTATTAAAAGCATTCTGTAACAAAGCGGGACCAAAGCCATGACAAAAACGCGTAACAAAAGTGTCTATAATCACGGCAGAAAAGTCCACATTGATTATTTGCACGGCGTCACACTTTGCTATGCCATAACATTTTTATCCATAAGATTAGCGGATCCTACCTGACGCTTTTTATCGCAACTCTCTACTGTTTCTCCATACCCGTTTTTTTGGGCTAGC
T500	CAAAGCCCGCCGAAAGGCGGGCTTTTTTTT
TL3S2P55	CTCGGTACCAAAGACGAACAATAAGACGCTGAAAAGCGTCTTTTTTCGTTTTGGTCC
TTonB	CCTCCGACCGGAGGCTTTTGACT
TrrnB	GAAGCTTGGGCCCGAACAAAAACTCATCTCAGAAGAGGATCTGAATAGCGCCGTCGACCATCATCATCATCATCATTGAGTTTAAACGGTCTCCAGCTTGGCTGTTTTGGCGGATGAGAGAAGATTTTCAGCCTGATACAGATTAAATCAGAACGCAGAAGCGGTCTGATAAAACAGAATTTGCCTGGCGGCAGTAGCGCGGTGGTCCCACCTGACCCCATGCCGAACTCAGAAGTGAAACGCCGTAGCGCCGATGGTAGTGTGGGGTCTCCCCATGCGAGAGTAGGGAACTGCCAGGCATCAAATAAAACGAAAGGCTCAGTCGAAAGACTGGGCCTTTCGTTTTATCTGTTGTTTGTCGGTGAACT
Tdbl	CCAGGCATCAAATAAAACGAAAGGCTCAGTCGAAAGACTGGGCCTTTCGTTTTATCTGTTGTTTGTCGGTGAACGCTCTCTACTAGAGTCACACTGGCTCACCTTCGGGTGGGCCTTTCTGCGTTTATA
Ribozyme	AAAAGTTATCAGGCATGCACCTGGTAGCTAGTCTTTAAACCAATAGATTGCATCGGTTTAAAAGGCAAGACCGTCAAATTGCGGGAAAGGGGTCAACAGCCGTTCAGTACCAAGTCTCAGGGGAAACTTTGAGATGGCCTTGCAAAGGGTATGGTAATAAGCTGACGGACATGGTCCTAACCACGCAGCCAA

	GTCCTAAGTCAACAGATCTTCTGTTGATATGGATGCAGT<br>TCACAGACTAAATGTCGGTCGGGGAAGATGTATTCTTCT<br>CATAAGATATAGTCGGACCTCTCCTTAATGGGAGCTAGC<br>GGATGAAGTGATGCAACACTGGAGCCGCTGGGAACTAA<br>TTTGTATGCGAAAGTATATTGATTAGTTTTGGAGTACTCG	
dRibozyme	AAAAGTTATCAGGCATGCACCTGGTAGCTAGTCTTTAAA<br>CCAATAGATTGCATCGGTTTAAAAGGCAAGACCGTCAAA<br>TTGCGGGAAAGGGGTCAACAGCCGTTCAGTACCAAGTC<br>TCAGGGGAAACTTTGAGATGGCCTTGCAAAGGGTATGG<br>TAATAAGCTGACGGACATGGTCCTAACCACGCAGCCAA<br>GTCCTAAGTCAACAGATCTTCTGTTGATATGGATGCAGT<br>TCACAAACTAAATGTCGGTCGGGGAAGATGTATTCTTCT<br>CATAAGATATAGTCGGACCTCTCCTTAATGGGAGCTAGC<br>GGATGAAGTGATGCAACACTGGAGCCGCTGGGAACTAA<br>TTTGTATGCGAAAGTATATTGATTAGTTTTGGAGTACTCG	
sgRNA_01	AATTAGATGGTGATGTTAATGTTTTAGAGCTAGAAATAGC<br>AAGTTAAAATAAGGCTAGTCCGTTATCAACTTGAAAAAGT<br>GGCACCGAGTCGGTGCTTTTTTT	
mCherry2	ATGGTGAGTAAAGGAGAAGAAAACAACTTAGCTATCATT<br>AAAGAGTTCATGCGCTTCAAAGTTCACATGGAGGGTTCT<br>GTTAACGGTCACGAGTTCGAGATCGAAGGCGAAGGCGA<br>GGGCCGTCCGTATGAAGGCACCCAGACCGCCAAACTGA<br>AAGTGACTAAAGGCGGCCCGCTGCCTTTTGCGTGGGAC<br>ATCCTGAGCCCGCAATTTATGTACGGTTCTAAAGCGTAT<br>GTTAAACACCCAGCGGATATCCCGGACTATCTGAAGCTG<br>TCTTTTCCGGAAGGTTTCAACTGGGAACGCGTAATGAAT<br>TTTGAAGATGGTGGTGTCGTGACCGTCACTCAGGACTCC<br>TCCCTTCAGGATGGCGAGTTCATCTATAAAGTTAAACTG<br>CGTGGTACTAATTTTCCATCTGATGGCCCGGTGATGCAG<br>TGTAGGACGATGGGTTGGGAGGCGTCTACCGAACGCAT<br>GTATCCGGAAGATGGTGCGCTGAAAGGCGAAATTAAAC<br>AGCGCCTGAAACTGAAAGATGGCGGCCATTATGACGCT<br>GAAGTGAAAACCACGTACAAAGCCAAGAAACCTGTGCA<br>GCTGCCTGGCGCGTACAATGTGGATATTAAACTGGACAT<br>CTTATCTCATAATGAAGATTATACGATCGTAGAGCAATAT<br>GAGCGCGCGGAGGGTCGTCATTCTACCGGTGGCATGGA<br>TGAACTATACAAATAA	MVSKGEENNLAIIKEF<br>MRFKVHMEGSVNGH<br>EFEIEGEGEGRPYEG<br>TQTAKLKVTKGGPLP<br>FAWDILSPQFMYGSK<br>AYVKHPADIPDYLKL<br>SFPEGFNWERVMNF<br>EDGGVVTVTQDSSL<br>QDGEFIYKVKLRGTN<br>FPSDGPVMQCRTMG<br>WEASTERMYPEDGA<br>LKGEIKQRLKLKDGG<br>HYDAEVKTTYKAKKP<br>VQLPGAYNVDIKLDIL<br>SHNEDYTIVEQYERA<br>EGRHSTGGMDELYK<br>*
sfGFP	ATGAGCAAAGGAGAAGAACTTTTCACTGGAGTTGTCCCA<br>ATTCTTGTTGAATTAGATGGTGATGTTAATGGGCACAAAT<br>TTTCTGTCCGTGGAGAGGGTGAAGGTGATGCTACAAAC<br>GGAAAACTCACCCTTAAATTTATTTGCACTACTGGAAAAC<br>TACCTGTTCCGTGGCCAACACTTGTCACTACTCTGACCT<br>ATGGTGTTCAATGCTTTTCCCGTTATCCGGATCACATGA<br>AACGGCATGACTTTTTCAAGAGTGCCATGCCCGAAGGTT<br>ATGTACAGGAACGCACTATATCTTTCAAAGATGACGGGA<br>CCTACAAGACGCGTGCTGAAGTCAAGTTTGAAGGTGATA<br>CCCTTGTTAATCGTATCGAGTTAAAGGGTATTGATTTTAA<br>AGAAGATGGAAACATTCTTGGACACAAACTCGAGTACAA<br>CTTTAACTCACACAATGTATACATCACGGCAGACAAACA<br>AAAGAATGGAATCAAAGCTAACTTCAAAATTCGCCACAA<br>CGTTGAAGATGGTTCCGTTCAACTAGCAGACCATTATCA<br>ACAAAATACTCCAATTGGCGATGGCCCTGTCCTTTTACC<br>AGACAACCATTACCTGTCGACACAATCTGTCCTTTCGAA<br>AGATCCCAACGAAAAGCGTGACCACATGGTCCTTCTTGA<br>GTTTGTAACTGCTGCTGGGATTACACATGGCATGGATGA<br>GCTCTACAAATAA	MSKGEELFTGVVPIL<br>VELDGDVNGHKFSV<br>RGEGEGDATNGKLT<br>LKFICTTGKLPVPWP<br>TLVTTLTYGVQCFSR<br>YPDHMKRHDFFKSA<br>MPEGYVQERTISFKD<br>DGTYKTRAEVKFEG<br>DTLVNRIELKGIDFKE<br>DGNILGHKLEYNFNS<br>HNVYITADKQKNGIK<br>ANFKIRHNVEDGSVQ<br>LADHYQQNTPIGDGP<br>VLLPDNHYLSTQSVL<br>SKDPNEKRDHMVLL<br>EFVTAAGITHGMDEL<br>YK*

FMO	ATGGCGACCCGTATTGCAATTCTGGGCGCAGGCCCATC<br>GGGTATGGCGCAATTGCGTGCGTTTCAAAGCGCACAAG<br>AGAAAGGCGCTGAGATCCCGGAGTTGGTTTGTTTTGAGA<br>AACAGGCGGACTGGGGTGGCCAGTGGAACTATACTTGG<br>CGTACCGGTCTGGACGAAAACGGCGAACCGGTCCACAG<br>CTCCATGTACCGTTACCTGTGGTCCAACGGTCCGAAAGA<br>ATGTTTGGAGTTTGCTGATTACACCTTTGATGAACACTTT<br>GGTAAGCCAATTGCCAGCTACCCACCGCGTGAAGTGCT<br>GTGGGACTATATCAAGGGTCGCGTGGAAAAGGCGGGTG<br>TCCGCAAATACATCCGTTTCAATACCGCGGTTCGTCATG<br>TTGAGTTCAATGAGGATTCTCAGACCTTTACTGTGACGG<br>TTCAGGACCATACCACTGACACCATCTATAGCGAAGAAT<br>TTGACTATGTGGTTTGCTGTACCGGTCACTTCAGCACCC<br>CGTATGTCCCGGAGTTCGAAGGCTTCGAAAAGTTCGGT<br>GGTCGTATTCTGCATGCCCACGACTTTCGTGATGCGCTG<br>GAGTTCAAGGATAAGACCGTTCTGTTGGTGGGCAGCTC<br>GTACTCTGCGGAAGATATTGGCAGCCAGTGCTACAAGTA<br>TGGCGCGAAGAAACTGATTAGCTGCTATCGCACCGCAC<br>CGATGGGTTACAAATGGCCGGAGAACTGGGACGAGCGT<br>CCGAACCTGGTGCGTGTGGATACCGAGAATGCTTACTTC<br>GCAGATGGTTCTTCGGAGAAAGTTGATGCCATCATCCTG<br>TGCACCGGTTACATCCACCACTTCCCGTTTCTGAATGAC<br>GACTTGCGCCTGGTGACCAACAATCGCCTGTGGCCGCT<br>GAACCTGTACAAGGGCGTTGTTTGGGAGGATAATCCGA<br>AGTTCTTCTACATTGGTATGCAAGACCAATGGTACAGCT<br>TCAACATGTTCGATGCCCAAGCTTGGTATGCGCGTGATG<br>TGATCATGGGCCGTTTGCCGTTGCCGAGCAAAGAAGAA<br>ATGAAGGCCGACAGCATGGCGTGGCGCGAGAAAGAGCT<br>GACGCTGGTCACGGCTGAAGAGATGTATACCTACCAGG<br>GTGACTATATCCAGAACCTGATCGACATGACCGATTATC<br>CGAGCTTTGATATTCCGGCGACGAACAAAACGTTCCTGG<br>AATGGAAACATCATAAGAAAGAGAACATCATGACGTTTC<br>GCGACCACAGCTATCGCTCCCTGATGACCGGCACGATG<br>GCTCCAAAGCACCATACCCCGTGGATCGATGCTCTGGA<br>CGACAGCCTGGAGGCTTACCTGAGCGACAAGTCCGAAA<br>TCCCGGTGGCAAAAGAGGCCTGATAA	MATRIAILGAGPSGM<br>AQLRAFQSAQEKGA<br>EIPELVCFEKQADWG<br>GQWNYTWRTGLDE<br>NGEPVHSSMYRYLW<br>SNGPKECLEFADYTF<br>DEHFGKPIASYPPRE<br>VLWDYIKGRVEKAGV<br>RKYIRFNTAVRHVEF<br>NEDSQTFTVTVQDHT<br>TDTIYSEEFDYVVCC<br>TGHFSTPYVPEFEGF<br>EKFGGRILHAHDFRD<br>ALEFKDKTVLLVGSS<br>YSAEDIGSQCYKYGA<br>KKLISCYRTAPMGYK<br>WPENWDERPNLVRV<br>DTENAYFADGSSEKV<br>DAIILCTGYIHHFPFLN<br>DDLRLVTNNRLWPLN<br>LYKGVVWEDNPKFF<br>YIGMQDQWYSFNMF<br>DAQAWYARDVIMGR<br>LPLPSKEEMKADSM<br>AWREKELTLVTAEEM<br>YTYQGDYIQNLIDMT<br>DYPSFDIPATNKTFLE<br>WKHHKKENIMTFRD<br>HSYRSLMTGTMAPK<br>HHTPWIDALDDSLEA<br>YLSDKSEIPVAKEA**
MHT	ATGAGCACCGTGGCGAACATTGCGCCGGTGTTTACCGG<br>CGATTGCAAAACCATTCCGACCCCGGAAGAATGCGCGA<br>CCTTTCTGTATAAAGTGGTGAACAGCGGCGGCTGGGAA<br>AAATGCTGGGTGGAAGAAGTGATTCCGTGGGATCTGGG<br>CGTGCCGACCCCGCTGGTGCTGCATCTGGTGAAAAACA<br>ACGCGCTGCCGAACGGCAAAGGCCTGGTGCCGGGCTG<br>CGGCGGCGGCTATGATGTGGTGGCGATGGCGAACCCG<br>GAACGCTTTATGGTGGGCCTGGATATTAGCGAAAACGC<br>GCTGAAAAAAGCGCGCGAAACCTTTAGCACCATGCCGA<br>ACAGCAGCTGCTTTAGCTTTGTGAAAGAAGATGTGTTTA<br>CCTGGCGCCCGGAACAGCCGTTTGATTTTATTTTTGATT<br>ATGTGTTTTTTTGCGCGATTGATCCGAAAATGCGCCCGG<br>CGTGGGGCAAAGCGTATGAACTGCTGAAACCGGATGGC<br>GAACTGATTACCCTGATGTATCCGATTACCAACCATGAA<br>GGCGGCCCGCCGTTTAGCGTGAGCGAAAGCGAATATGA<br>AAAAGTGCTGGTGCCGCTGGGCTTTAAACAGCTGAGCC<br>TGGAAGATTATAGCGATCTGGCGGTGGAACCGCGCAAA<br>GGCAAAGAAAAACTGGCGCGCTGGAAAAAAATGAACAA<br>CTGA	MSTVANIAPVFTGDC<br>KTIPTPEECATFLYKV<br>VNSGGWEKCWVEE<br>VIPWDLGVPTPLVLH<br>LVKNNALPNGKGLVP<br>GCGGGYDVVAMANP<br>ERFMVGLDISENALK<br>KARETFSTMPNSSCF<br>SFVKEDVFTWRPEQ<br>PFDFIFDYVFFCAIDP<br>KMRPAWGKAYELLK<br>PDGELITLMYPITNHE<br>GGPPFSVSESEYEK<br>VLVPLGFKQLSLEDY<br>SDLAVEPRKGKEKLA<br>RWKKMNN*

dCas9	ATGGACAAGAAGTATTCTATCGGACTGGCTATCGGGACT	MDKKYSIGLAIGTNS
	AATAGCGTCGGGTGGGCCGTGATCACTGACGAGTACAA	VGWAVITDEYKVPSK
	GGTGCCCTCTAAGAAGTTCAAGGTGCTCGGGAACACCG	KFKVLGNTDRHSIKK
	ACCGGCATTCCATCAAGAAAAATCTGATCGGAGCTCTCC	NLIGALLFDSGETAEA
	TCTTTGATTCAGGGGAAACCGCTGAAGCAACCCGCCTCA	TRLKRTARRRYTRRK
	AGCGGACTGCTAGACGGCGGTACACCAGGAGGAAGAAC	NRICYLQEIFSNEMA
	CGGATTTGTTACCTTCAAGAGATATTCTCCAACGAAATG	KVDDSFFHRLEESFL
	GCAAAGGTCGACGACAGCTTCTTCCATAGGCTGGAAGA	VEEDKKHERHPIFGN
	ATCATTCCTCGTGGAAGAGGATAAGAAGCATGAACGGCA	IVDEVAYHEKYPTIYH
	TCCCATCTTCGGTAATATCGTCGACGAGGTGGCCTATCA	LRKKLVDSTDKADLR
	CGAGAAATACCCAACCATCTACCATCTTCGCAAAAAGCT	LIYLALAHMIKFRGHF
	GGTGGACTCAACCGACAAGGCAGACCTCCGGCTTATCT	LIEGDLNPDNSDVDK
	ACCTGGCCCTGGCCCACATGATCAAGTTCAGAGGCCAC	LFIQLVQTYNQLFEE
	TTCCTGATCGAGGGCGACCTCAATCCTGACAATAGCGAT	NPINASGVDAKAILSA
	GTGGATAAACTGTTCATCCAGCTGGTGCAGACTTACAAC	RLSKSRRLENLIAQL
	CAGCTCTTTGAAGAGAACCCCATCAATGCAAGCGGAGTC	PGEKKNGLFGNLIAL
	GATGCCAAGGCCATTCTGTCAGCCCGGCTGTCAAAGAG	SLGLTPNFKSNFDLA
	CCGCAGACTTGAGAATCTTATCGCTCAGCTGCCGGGTG	EDAKLQLSKDTYDDD
	AAAAGAAAAATGGACTGTTCGGGAACCTGATTGCTCTTT	LDNLLAQIGDQYADL
	CACTTGGGCTGACTCCCAATTTCAAGTCTAATTTCGACC	FLAAKNLSDAILLSDIL
	TGGCAGAGGATGCCAAGCTGCAACTGTCCAAGGACACC	RVNTEITKAPLSASMI
	TATGATGACGATCTCGACAACCTCCTGGCCCAGATCGGT	KRYDEHHQDLTLLKA
	GACCAATACGCCGACCTTTTCCTTGCTGCTAAGAATCTT	LVRQQLPEKYKEIFF
	TCTGACGCCATCCTGCTGTCTGACATTCTCCGCGTGAAC	DQSKNGYAGYIDGG
	ACTGAAATCACCAAGGCCCCTCTTTCAGCTTCAATGATT	ASQEEFYKFIKPILEK
	AAGCGGTATGATGAGCACCACCAGGACCTGACCCTGCT	MDGTEELLVKLNRED
	TAAGGCACTCGTCCGGCAGCAGCTTCCGGAGAAGTACA	LLRKQRTFDNGSIPH
	AGGAAATCTTCTTTGACCAGTCAAAGAATGGATACGCCG	QIHLGELHAILRRQED
	GCTACATCGACGGAGGTGCCTCCCAAGAGGAATTTTATA	FYPFLKDNREKIEKIL
	AGTTTATCAAACCTATCCTTGAGAAGATGGACGGCACCG	TFRIPYYVGPLARGN
	AAGAGCTCCTCGTGAAACTGAATCGGGAGGATCTGCTG	SRFAWMTRKSEETIT
	CGGAAGCAGCGCACTTTCGACAATGGGAGCATTCCCCA	PWNFEEVVDKGASA
	CCAGATCCATCTTGGGGAGCTTCACGCCATCCTTCGGC	QSFIERMTNFDKNLP
	GCCAAGAGGACTTCTACCCCTTTCTTAAGGACAACAGGG	NEKVLPKHSLLYEYF
	AGAAGATTGAGAAAATTCTCACTTTCCGCATCCCCTACTA	TVYNELTKVKYVTEG
	CGTGGGACCCCTCGCCAGAGGAAATAGCCGGTTTGCTT	MRKPAFLSGEQKKAI
	GGATGACCAGAAAGTCAGAAGAAACTATCACTCCCTGGA	VDLLFKTNRKVTVKQ
	ACTTCGAAGAGGTGGTGGACAAGGGAGCCAGCGCTCAG	LKEDYFKKIECFDSV
	TCATTCATCGAACGGATGACTAACTTCGATAAGAACCTC	EISGVEDRFNASLGT
	CCCAATGAGAAGGTCCTGCCGAAACATTCCCTGCTCTAC	YHDLLKIIKDKDFLDN
	GAGTACTTTACCGTGTACAACGAGCTGACCAAGGTGAAA	EENEDILEDIVLTLTLF
	TATGTCACCGAAGGGATGAGGAAGCCCGCATTCCTGTC	EDREMIEERLKTYAH
	AGGCGAACAAAAGAAGGCAATTGTGGACCTTCTGTTCAA	LFDDKVMKQLKRRR
	GACCAATAGAAAGGTGACCGTGAAGCAGCTGAAGGAGG	YTGWGRLSRKLINGI
	ACTATTTCAAGAAAATTGAATGCTTCGACTCTGTGGAGAT	RDKQSGKTILDFLKS
	TAGCGGGGTCGAAGATCGGTTCAACGCAAGCCTGGGTA	DGFANRNFMQLIHDD
	CTTACCATGATCTGCTTAAGATCATCAAGGACAAGGATTT	SLTFKEDIQKAQVSG
	TCTGGACAATGAGGAGAACGAGGACATCCTTGAGGACA	QGDSLHEHIANLAGS
	TTGTCCTGACTCTCACTCTGTTCGAGGACCGGGAAATGA	PAIKKGILQTVKVVDE
	TCGAGGAGAGGCTTAAGACCTACGCCCATCTGTTCGAC	LVKVMGRHKPENIVI
	GATAAAGTGATGAAGCAACTTAAACGGAGAAGATATACC	EMARENQTTQKGQK
	GGATGGGGACGCCTTAGCCGCAAACTCATCAACGGAAT	NSRERMKRIEEGIKE
	CCGGGACAAACAGAGCGGAAAGACCATTCTTGATTTCCT	LGSQILKEHPVENTQ
	TAAGAGCGACGGATTCGCTAATCGCAACTTCATGCAACT	LQNEKLYLYYLQNGR
	TATCCATGATGATTCCCTGACCTTTAAGGAGGACATCCA	DMYVDQELDINRLSD
	GAAGGCCCAAGTGTCTGGACAAGGTGACTCACTGCACG	YDVDAIVPQSFLADD

	AGCATATCGCAAATCTGGCTGGTTCACCCGCTATTAAGA<br>AGGGTATTCTCCAGACCGTGAAAGTCGTGGACGAGCTG<br>GTCAAGGTGATGGGTCGCCATAAACCAGAGAACATTGTC<br>ATCGAGATGGCCAGGGAAAACCAGACTACCCAGAAGGG<br>ACAGAAGAACAGCAGGGAGCGGATGAAAAGAATTGAGG<br>AAGGGATTAAGGAGCTCGGGTCACAGATCCTTAAAGAG<br>CACCCGGTGGAAAACACCCAGCTTCAGAATGAGAAGCT<br>CTATCTGTACTACCTTCAAAATGGACGCGATATGTATGT<br>GGACCAAGAGCTTGATATCAACAGGCTCTCAGACTACGA<br>CGTGGACGCCATCGTCCCTCAGAGCTTCCTCGCAGACG<br>ACTCAATTGACAATAAGGTGCTGACTCGCTCAGACAAGA<br>ACCGGGGAAAGTCAGATAACGTGCCCTCAGAGGAAGTC<br>GTGAAAAAGATGAAGAACTATTGGCGCCAGCTTCTGAAC<br>GCAAAGCTGATCACTCAGCGGAAGTTCGACAATCTCACT<br>AAGGCTGAGAGGGGCGGACTGAGCGAACTGGACAAAG<br>CAGGATTCATTAAACGGCAACTTGTGGAGACTCGGCAGA<br>TTACTAAACATGTCGCCCAAATCCTTGACTCACGCATGA<br>ATACCAAGTACGACGAAAACGACAAACTTATCCGCGAGG<br>TGAAGGTGATTACCCTGAAGTCCAAGCTGGTCAGCGATT<br>TCAGAAAGGACTTTCAATTCTACAAAGTGCGGGAGATCA<br>ATAACTATCATCATGCTCATGACGCATATCTGAATGCCGT<br>GGTGGGAACCGCCCTGATCAAGAAGTACCCAGCACTGG<br>AAAGCGAGTTCGTGTACGGAGACTACAAGGTCTACGAC<br>GTGCGCAAGATGATTGCCAAATCTGAGCAGGAGATCGG<br>AAAGGCCACCGCAAAGTACTTCTTCTACAGCAACATCAT<br>GAATTTCTTCAAGACCGAAATCACCCTTGCAAACGGTGA<br>GATCCGGAAGGCGCCGCTCATCGAGACTAATGGGGAGA<br>CTGGCGAAATCGTGTGGGACAAGGGCAGAGATTTCGCT<br>ACCGTGCGCAAAGTGCTTTCTATGCCTCAAGTGAACATC<br>GTGAAGAAAACCGAGGTGCAAACCGGAGGCTTTTCTAA<br>GGAATCAATCCTCCCCAAGCGCAACTCCGACAAGCTCAT<br>TGCAAGGAAGAAGGATTGGGACCCTAAGAAGTACGGCG<br>GATTCGATTCACCAACTGTGGCTTATTCTGTCCTGGTCG<br>TGGCTAAGGTGGAAAAAGGAAAGTCTAAGAAGCTCAAGA<br>GCGTGAAGGAACTGCTGGGTATCACCATTATGGAGCGC<br>AGCTCCTTCGAGAAGAACCCAATTGACTTTCTCGAAGCC<br>AAAGGTTACAAGGAAGTCAAGAAGGACCTTATCATCAAG<br>CTCCCAAAGTATAGCCTGTTCGAACTGGAGAATGGGCG<br>GAAGCGGATGCTCGCCTCCGCTGGCGAACTTCAGAAGG<br>GTAATGAGCTGGCTCTCCCCTCCAAGTACGTGAATTTCC<br>TCTACCTTGCAAGCCATTACGAGAAGCTGAAGGGGAGC<br>CCCGAGGACAACGAGCAAAAGCAACTGTTTGTGGAGCA<br>GCATAAGCATTATCTGGACGAGATCATTGAGCAGATTTC<br>CGAGTTTTCTAAACGCGTCATTCTCGCTGATGCCAACCT<br>CGATAAAGTCCTTAGCGCATACAATAAGCACAGAGACAA<br>ACCAATTCGGGAGCAGGCTGAGAATATCATCCACCTGTT<br>CACCCTCACCAATCTTGGTGCCCCTGCCGCATTCAAGTA<br>CTTCGACACCACCATCGACCGGAAACGCTATACCTCCAC<br>CAAAGAAGTGCTGGACGCCACCCTCATCCACCAGAGCA<br>TCACCGGACTTTACGAAACTCGGATTGACCTCTCACAGC<br>TCGGAGGGGATTGA	SIDNKVLTRSDKNRG<br>KSDNVPSEEVVKKM<br>KNYWRQLLNAKLITQ<br>RKFDNLTKAERGGLS<br>ELDKAGFIKRQLVET<br>RQITKHVAQILDSRM<br>NTKYDENDKLIREVK<br>VITLKSKLVSDFRKDF<br>QFYKVREINNYHHAH<br>DAYLNAVVGTALIKK<br>YPALESEFVYGDYKV<br>YDVRKMIAKSEQEIG<br>KATAKYFFYSNIMNF<br>FKTEITLANGEIRKAP<br>LIETNGETGEIVWDK<br>GRDFATVRKVLSMP<br>QVNIVKKTEVQTGGF<br>SKESILPKRNSDKLIA<br>RKKDWDPKKYGGFD<br>SPTVAYSVLVVAKVE<br>KGKSKKLKSVKELLG<br>ITIMERSSFEKNPIDF<br>LEAKGYKEVKKDLIIK<br>LPKYSLFELENGRKR<br>MLASAGELQKGNEL<br>ALPSKYVNFLYLASH<br>YEKLKGSPEDNEQK<br>QLFVEQHKHYLDEIIE<br>QISEFSKRVILADANL<br>DKVLSAYNKHRDKPI<br>REQAENIIHLFTLTNL<br>GAPAAFKYFDTTIDR<br>KRYTSTKEVLDATLIH<br>QSITGLYETRIDLSQL<br>GGD*
T7 RNAP	ATGAACACGATTAACATCGCTAAGAACGACTTCTCTGAC<br>ATCGAACTGGCTGCTATCCCGTTCAACACTCTGGCTGAC<br>CATTACGGTGAGCGTTTAGCTCGCGAACAGTTGGCCCTT<br>GAGCATGAGTCTTACGAGATGGGTGAAGCACGCTTCCG<br>CAAGATGTTTGAGCGTCAACTTAAAGCTGGTGAGGTTGC	MNTINIAKNDFSDIEL<br>AAIPFNTLADHYGER<br>LAREQLALEHESYEM<br>GEARFRKMFERQLK<br>AGEVADNAAAKPLIT

	GGATAACGCTGCCGCCAAGCCTCTCATCACTACCCTACT<br>CCCTAAGATGATTGCACGCATCAACGACTGGTTTGAGGA<br>AGTGAAAGCTAAGCGCGGCAAGCGCCCGACAGCCTTCC<br>AGTTCCTGCAAGAAATCAAGCCGGAAGCCGTAGCGTAC<br>ATCACCATTAAGACCACTCTGGCTTGCCTAACCAGTGCT<br>GACAATACAACCGTTCAGGCTGTAGCAAGCGCAATCGG<br>TCGGGCCATTGAGGACGAGGCTCGCTTCGGTCGTATCC<br>GTGACCTTGAAGCTAAGCACTTCAAGAAAAACGTTGAGG<br>AACAACTCAACAAGCGCGTAGGGCACGTCTACAAGAAA<br>GCATTTATGCAAGTTGTCGAGGCTGACATGCTCTCTAAG<br>GGTCTACTCGGTGGCGAGGCGTGGTCTTCGTGGCATAA<br>GGAAGACTCTATTCATGTAGGAGTACGCTGCATCGAGAT<br>GCTCATTGAGTCAACCGGAATGGTTAGCTTACACCGCCA<br>AAATGCTGGCGTAGTAGGTCAAGACTCTGAGACTATCGA<br>ACTCGCACCTGAATACGCTGAGGCTATCGCAACCCGTG<br>CAGGTGCGCTGGCTGGCATCTCTCCGATGTTCCAACCTT<br>GCGTAGTTCCTCCTAAGCCGTGGACTGGCATTACTGGT<br>GGTGGCTATTGGGCTAACGGTCGTCGTCCTCTGGCGCT<br>GGTGCGTACTCACAGTAAGAAAGCACTGATGCGCTACG<br>AAGACGTTTACATGCCTGAGGTGTACAAAGCGATTAACA<br>TTGCGCAAAACACCGCATGGAAAATCAACAAGAAAGTCC<br>TAGCGGTCGCCAACGTAATCACCAAGTGGAAGCATTGTC<br>CGGTCGAGGACATCCCTGCGATTGAGCGTGAAGAACTC<br>CCGATGAAACCGGAAGACATCGACATGAATCCTGAGGC<br>TCTCACCGCGTGGAAACGTGCTGCCGCTGCTGTGTACC<br>GCAAGGACAAGGCTCGCAAGTCTCGCCGTATCAGCCTT<br>GAGTTCATGCTTGAGCAAGCCAATAAGTTTGCTAACCAT<br>AAGGCCATCTGGTTCCCTTACAACATGGACTGGCGCGG<br>TCGTGTTTACGCTGTGTCAATGTTCAACCCGCAAGGTAA<br>CGATATGACCAAAGGACTGCTTACGCTGGCGAAAGGTA<br>AACCAATCGGTAAGGAAGGTTACTACTGGCTGAAAATCC<br>ACGGTGCAAACTGTGCGGGTGTCGACAAGGTTCCGTTC<br>CCTGAGCGCATCAAGTTCATTGAGGAAAACCACGAGAAC<br>ATCATGGCTTGCGCTAAGTCTCCACTGGAGAACACTTGG<br>TGGGCTGAGCAAGATTCTCCGTTCTGCTTCCTTGCGTTC<br>TGCTTTGAGTACGCTGGGGTACAGCACCACGGCCTGAG<br>CTATAACTGCTCCCTTCCGCTGGCGTTTGACGGGTCTTG<br>CTCTGGCATCCAGCACTTCTCCGCGATGCTCCGAGATG<br>AGGTAGGTGGTCGCGCGGTTAACTTGCTTCCTAGTGAAA<br>CCGTTCAGGACATCTACGGGATTGTTGCTAAGAAAGTCA<br>ACGAGATTCTACAAGCAGACGCAATCAATGGGACCGATA<br>ACGAAGTAGTTACCGTGACCGATGAGAACACTGGTGAAA<br>TCTCTGAGAAAGTCAAGCTGGGCACTAAGGCACTGGCT<br>GGTCAATGGCTGGCTTACGGTGTTACTCGCAGTGTGACT<br>AAGAGTTCAGTCATGACGCTGGCTTACGGGTCCAAAGA<br>GTTCGGCTTCCGTCAACAAGTGCTGGAAGATACCATTCA<br>GCCAGCTATTGATTCCGGCAAGGGTCTGATGTTCACTCA<br>GCCGAATCAGGCTGCTGGATACATGGCTAAGCTGATTTG<br>GGAATCTGTGAGCGTGACGGTGGTAGCTGCGGTTGAAG<br>CAATGAACTGGCTTAAGTCTGCTGCTAAGCTGCTGGCTG<br>CTGAGGTCAAAGATAAGAAGACTGGAGAGATTCTTCGCA<br>AGCGTTGCGCTGTGCATTGGGTAACTCCTGATGGTTTCC<br>CTGTGTGGCAGGAATACAAGAAGCCTATTCAGACGCGC<br>TTGAACCTGATGTTCCTCGGTCAGTTCCGCTTACAGCCT<br>ACCATTAACACCAACAAAGATAGCGAGATTGATGCACAC<br>AAACAGGAGTCTGGTATCGCTCCTAACTTTGTACACAGC	TLLPKMIARINDWFEE<br>VKAKRGKRPTAFQFL<br>QEIKPEAVAYITIKTTL<br>ACLTSADNTTVQAVA<br>SAIGRAIEDEARFGRI<br>RDLEAKHFKKNVEEQ<br>LNKRVGHVYKKAFM<br>QVVEADMLSKGLLG<br>GEAWSSWHKEDSIH<br>VGVRCIEMLIESTGM<br>VSLHRQNAGVVGQD<br>SETIELAPEYAEAIAT<br>RAGALAGISPMFQPC<br>VVPPKPWTGITGGG<br>YWANGRRPLALVRT<br>HSKKALMRYEDVYM<br>PEVYKAINIAQNTAW<br>KINKKVLAVANVITKW<br>KHCPVEDIPAIEREEL<br>PMKPEDIDMNPEALT<br>AWKRAAAAVYRKDK<br>ARKSRRISLEFMLEQ<br>ANKFANHKAIWFPYN<br>MDWRGRVYAVSMF<br>NPQGNDMTKGLLTL<br>AKGKPIGKEGYYWLK<br>IHGANCAGVDKVPFP<br>ERIKFIEENHENIMAC<br>AKSPLENTWWAEQD<br>SPFCFLAFCFEYAGV<br>QHHGLSYNCSLPLAF<br>DGSCSGIQHFSAMLR<br>DEVGGRAVNLLPSET<br>VQDIYGIVAKKVNEIL<br>QADAINGTDNEVVTV<br>TDENTGEISEKVKLG<br>TKALAGQWLAYGVT<br>RSVTKSSVMTLAYGS<br>KEFGFRQQVLEDTIQ<br>PAIDSGKGLMFTQPN<br>QAAGYMAKLIWESVS<br>VTVVAAVEAMNWLK<br>SAAKLLAAEVKDKKT<br>GEILRKRCAVHWVTP<br>DGFPVWQEYKKPIQT<br>RLNLMFLGQFRLQPT<br>INTNKDSEIDAHKQE<br>SGIAPNFVHSQDGSH<br>LRKTVVWAHEKYGIE<br>SFALIHDSFGTIPADA<br>ANLFKAVRETMVDTY<br>ESCDVLADFYDQFAD<br>QLHESQLDKMPALP<br>AKGNLNLRDILESDF<br>AFA*

	CAAGACGGTAGCCACCTTCGTAAGACTGTAGTGTGGGC<br>ACACGAGAAGTACGGAATCGAATCTTTTGCACTGATTCA<br>CGACTCCTTCGGTACGATTCCGGCTGACGCTGCGAACC<br>TGTTCAAAGCAGTGCGCGAAACTATGGTTGACACATATG<br>AGTCTTGTGATGTACTGGCTGATTTCTACGACCAGTTCG<br>CTGACCAGTTGCACGAGTCTCAATTGGACAAAATGCCAG<br>CACTTCCGGCTAAAGGTAACTTGAACCTCCGTGACATCT<br>TAGAGTCGGACTTCGCGTTCGCGTAA	
AraC	ATGGCTGAAGCGCAAAATGATCCCCTGCTGCCGGGATA<br>CTCGTTTAATGCCCATCTGGTGGCGGGTTTAACGCCGAT<br>TGAGGCCAACGGTTATCTCGATTTTTTTATCGACCGACC<br>GCTGGGAATGAAAGGTTATATTCTCAATCTCACCATTCG<br>CGGTCAGGGGGTGGTGAAAAATCAGGGACGAGAATTTG<br>TTTGCCGACCGGGTGATATTTTGCTGTTCCCGCCAGGAG<br>AGATTCATCACTACGGTCGTCATCCGGAGGCTCGCGAAT<br>GGTATCACCAGTGGGTTTACTTTCGTCCGCGCGCCTACT<br>GGCATGAATGGCTTAACTGGCCGTCAATATTTGCCAATA<br>CGGGGTTCTTTCGCCCGGATGAAGCGCACCAGCCGCAT<br>TTCAGCGACCTGTTTGGGCAAATCATTAACGCCGGGCAA<br>GGGGAAGGGCGCTATTCGGAGCTGCTGGCGATAAATCT<br>GCTTGAGCAATTGTTACTGCGGCGCATGGAAGCGATTAA<br>CGAGTCGCTCCATCCACCGATGGATAATCGGGTACGCG<br>AGGCTTGTCAGTACATCAGCGATCACCTGGCAGACAGC<br>AATTTTGATATCGCCAGCGTCGCACAGCATGTTTGCTTG<br>TCGCCGTCGCGTCTGTCACATCTTTTCCGCCAGCAGTTA<br>GGGATTAGCGTCTTAAGCTGGCGCGAGGACCAACGTAT<br>CAGCCAGGCGAAGCTGCTTTTGAGCACCACCCGGATGC<br>CTATCGCCACCGTCGGTCGCAATGTTGGTTTTGACGATC<br>AACTCTATTTCTCGCGGGTATTTAAAAAATGCACCGGGG<br>CCAGCCCGAGCGAGTTCCGTGCCGGTTGTGAAGAAAAA<br>GTGAATGATGTAGCCGTCAAGTTGTCATAA	MAEAQNDPLLPGYS<br>FNAHLVAGLTPIEAN<br>GYLDFFIDRPLGMKG<br>YILNLTIRGQGVVKN<br>QGREFVCRPGDILLF<br>PPGEIHHYGRHPEAR<br>EWYHQWVYFRPRAY<br>WHEWLNWPSIFANT<br>GFFRPDEAHQPHFS<br>DLFGQIINAGQGEGR<br>YSELLAINLLEQLLLR<br>RMEAINESLHPPMDN<br>RVREACQYISDHLAD<br>SNFDIASVAQHVCLS<br>PSRLSHLFRQQLGIS<br>VLSWREDQRISQAKL<br>LLSTTRMPIATVGRN<br>VGFDDQLYFSRVFKK<br>CTGASPSEFRAGCE<br>EKVNDVAVKLS*
RFP	ATGGCGAGTAGCGAAGACGTTATCAAAGAGTTCATGCGT<br>TTCAAAGTTCGTATGGAAGGTTCCGTTAACGGTCACGAG<br>TTCGAAATCGAAGGTGAAGGTGAAGGTCGTCCGTACGA<br>AGGTACCCAGACCGCTAAACTGAAAGTTACCAAAGGTG<br>GTCCGCTGCCGTTCGCTTGGGACATCCTGTCCCCGCAG<br>TTCCAGTACGGTTCCAAAGCTTACGTTAAACACCCGGCT<br>GACATCCCGGACTACCTGAAACTGTCCTTCCCGGAAGG<br>TTTCAAATGGGAACGTGTTATGAACTTCGAAGACGGTGG<br>TGTTGTTACCGTTACCCAGGACTCCTCCCTGCAAGACGG<br>TGAGTTCATCTACAAAGTTAAACTGCGTGGTACCAACTT<br>CCCGTCCGACGGTCCGGTTATGCAGAAAAAAACCATGG<br>GTTGGGAAGCTTCCACCGAACGTATGTACCCGGAAGAC<br>GGTGCTCTGAAAGGTGAAATCAAAATGCGTCTGAAACTG<br>AAAGACGGTGGTCACTACGACGCTGAAGTTAAAACCACC<br>TACATGGCTAAAAAACCGGTTCAGCTGCCGGGTGCTTAC<br>AAAACCGACATCAAACTGGACATCACCTCCCACAACGAA<br>GACTACACCATCGTTGAACAGTACGAACGTGCTGAAGGT<br>CGTCACTCCACCGGTGCTTAA	MASSEDVIKEFMRFK<br>VRMEGSVNGHEFEIE<br>GEGEGRPYEGTQTA<br>KLKVTKGGPLPFAW<br>DILSPQFQYGSKAYV<br>KHPADIPDYLKLSFP<br>EGFKWERVMNFEDG<br>GVVTVTQDSSLQDG<br>EFIYKVKLRGTNFPS<br>DGPVMQKKTMGWE<br>ASTERMYPEDGALK<br>GEIKMRLKLKDGGHY<br>DAEVKTTYMAKKPV<br>QLPGAYKTDIKLDITS<br>HNEDYTIVEQYERAE<br>GRHSTGA*
Catechol 2,3-<br>diooxygenas<br>e	ATGAACAAAGGTGTAATGCGACCGGGCCATGTGCAGCT<br>GCGTGTACTGGACATGAGCAAGGCCCTGGAACACTACG<br>TCGAGTTGCTGGGCCTGATCGAGATGGACCGTGACGAC<br>CAGGGCCGTGTCTATCTGAAGGCTTGGACCGAAGTGGA<br>TAAGTTTTCCCTGGTGCTACGCGAGGCTGACGAGCCGG<br>GCATGGATTTTATGGGTTTCAAGGTTGTGGATGAGGATG<br>CTCTCCGGCAACTGGAGCGGGATCTGATGGCATATGGC	MNKGVMRPGHVQLR<br>VLDMSKALEHYVELL<br>GLIEMDRDDQGRVYL<br>KAWTEVDKFSLVLRE<br>ADEPGMDFMGFKVV<br>DEDALRQLERDLMA<br>YGCAVEQLPAGELN

	TGTGCCGTTGAGCAGCTACCCGCAGGTGAACTGAACAG<br>TTGTGGCCGGCGCGTGCGCTTCCAGGCCCCCTCCGGG<br>CATCACTTCGAGTTGTATGCAGACAAGGAATATACTGGA<br>AAGTGGGGTTTGAATGACGTCAATCCCGAGGCATGGCC<br>GCGCGATCTGAAAGGTATGGCGGCTGTGCGTTTCGACC<br>ACGCCCTCATGTATGGCGACGAATTGCCGGCGACCTAT<br>GACCTGTTCACCAAGGTGCTCGGTTTCTATCTGGCCGAA<br>CAGGTGCTGGACGAAAATGGCACGCGCGTCGCCCAGTT<br>TCTCAGTCTGTCGACCAAGGCCCACGACGTGGCCTTCA<br>TTCACCATCCGGAAAAAGGCCGCCTCCATCATGTGTCCT<br>TCCACCTCGAAACCTGGGAAGACTTGCTTCGCGCCGCC<br>GACCTGATCTCCATGACCGACACATCTATCGATATCGGC<br>CCAACCCGCCACGGCCTCACTCACGGCAAGACCATCTA<br>CTTCTTCGACCCGTCCGGTAACCGCAACGAAGTGTTCTG<br>CGGGGGAGATTACAACTACCCGGACCACAAACCGGTGA<br>CCTGGACCACCGACCAGCTGGGCAAGGCGATCTTTTAC<br>CACGACCGCATTCTCAACGAACGATTCATGACCGTGCTG<br>ACCTAA	SCGRRVRFQAPSGH<br>HFELYADKEYTGKW<br>GLNDVNPEAWPRDL<br>KGMAAVRFDHALMY<br>GDELPATYDLFTKVL<br>GFYLAEQVLDENGTR<br>VAQFLSLSTKAHDVA<br>FIHHPEKGRLHHVSF<br>HLETWEDLLRAADLI<br>SMTDTSIDIGPTRHG<br>LTHGKTIYFFDPSGN<br>RNEVFCGGDYNYPD<br>HKPVTWTTDQLGKAI<br>FYHDRILNERFMTVL<br>T*
TetA	ATGAAATCTAACAATGCGCTCATCGTCATCCTCGGCACC<br>GTCACCCTGGATGCTGTAGGCATAGGCTTGGTTATGCC<br>GGTACTGCCGGGCCTCTTGCGGGATATCGTCCATTCCG<br>ACAGCATCGCCAGTCACTATGGCGTGCTGCTAGCGCTA<br>TATGCGTTGATGCAATTTCTATGCGCACCCGTTCTCGGA<br>GCACTGTCCGACCGCTTTGGCCGCCGCCCAGTCCTGCT<br>CGCTTCGCTACTTGGAGCCACTATCGACTACGCGATCAT<br>GGCGACCACACCCGTCCTGTGGATCCTCTACGCCGGAC<br>GCATCGTGGCCGGCATCACCGGCGCCACAGGTGCGGT<br>TGCTGGCGCCTATATCGCCGACATCACCGATGGGGAAG<br>ATCGGGCTCGCCACTTCGGGCTCATGAGCGCTTGTTTC<br>GGCGTGGGTATGGTGGCAGGCCCCGTGGCCGGGGGAC<br>TGTTGGGCGCCATCTCCTTGCATGCACCATTCCTTGCGG<br>CGGCGGTGCTCAACGGCCTCAACCTACTACTGGGCTGC<br>TTCCTAATGCAGGAGTCGCATAAGGGAGAGCGTCGACC<br>GATGCCCTTGAGAGCCTTCAACCCAGTCAGCTCCTTCCG<br>GTGGGCGCGGGGCATGACTATCGTCGCCGCACTTATGA<br>CTGTCTTCTTTATCATGCAACTCGTAGGACAGGTGCCGG<br>CAGCGCTCTGGGTCATTTTCGGCGAGGACCGCTTTCGC<br>TGGAGCGCGACGATGATCGGCCTGTCGCTTGCGGTATT<br>CGGAATCTTGCACGCCCTCGCTCAAGCCTTCGTCACTG<br>GTCCCGCCACCAAACGTTTCGGCGAGAAGCAGGCCATT<br>ATCGCCGGCATGGCGGCCGACGCGCTGGGCTACGTCTT<br>GCTGGCGTTCGCGACGCGAGGCTGGATGGCCTTCCCCA<br>TTATGATTCTTCTCGCTTCCGGCGGCATCGGGATGCCCG<br>CGTTGCAGGCCATGCTGTCCAGGCAGGTAGATGACGAC<br>CATCAGGGACAGCTTCAAGGATCGCTCGCGGCTCTTAC<br>CAGCCTAACTTCGATCATTGGACCGCTGATCGTCACGGC<br>GATTTATGCCGCCTCGGCGAGCACATGGAACGGGTTGG<br>CATGGATTGTAGGCGCCGCCCTATACCTTGTCTGCCTCC<br>CCGCGTTGCGTCGCGGTGCATGGAGCCGGGCCACCTC<br>GACCTAA	MKSNNALIVILGTVTL<br>DAVGIGLVMPVLPGL<br>LRDIVHSDSIASHYGV<br>LLALYALMQFLCAPV<br>LGALSDRFGRRPVLL<br>ASLLGATIDYAIMATT<br>PVLWILYAGRIVAGIT<br>GATGAVAGAYIADIT<br>DGEDRARHFGLMSA<br>CFGVGMVAGPVAGG<br>LLGAISLHAPFLAAAV<br>LNGLNLLLGCFLMQE<br>SHKGERRPMPLRAF<br>NPVSSFRWARGMTI<br>VAALMTVFFIMQLVG<br>QVPAALWVIFGEDRF<br>RWSATMIGLSLAVFG<br>ILHALAQAFVTGPATK<br>RFGEKQAIIAGMAAD<br>ALGYVLLAFATRGW<br>MAFPIMILLASGGIGM<br>PALQAMLSRQVDDD<br>HQGQLQGSLAALTSL<br>TSIIGPLIVTAIYAASA<br>STWNGLAWIVGAALY<br>LVCLPALRRGAWSR<br>ATST*

Supplementary Table 6. Strains used in this study.

***Species*/Strain Name**	**Description/Genotype**
Escherichia coli	
MG1655	Wild type: F⁻ λ⁻ ilvG⁻ rfb-50 rph-1
TG1	K-12 *supE thi-1 Δ(lac-proAB) Δ(mcrB-hsdSM)5, ($r_K^-m_K^-$)* F' [*traD36 proAB*$^+$ *lacI*^{q} *lacZΔM15*]
NEB Turbo	Cloning strain: K-12 *glnV44 thi-1* Δ(*lac-proAB*) *galE15 galK16 R*(*zgb-210*::Tn*10*)Tet^S *endA1 fhuA2* Δ(*mcrB-hsdSM)5*($r_K^-m_K^-$) F'[*traD36 proAB*$^+$ *lacI*^{q} *lacZΔM15*]
Vibrio natriegens	
Vmax	14048 dns::LacI-T7-RNAP
Shewanella oneidensis	
MR-1	Wild type

Supplementary Table 7. Species growth conditions.

Species	*Escherichia coli*	*Vibrio natriegens*	*Shewanella oneidensis*
Growth temperature	37 °C	37 °C	30 °C
Antibiotic concentrations			
Carbenicillin	100 µg/mL	50 µg/mL	100 µg/mL
Spectinomycin	50 µg/mL	200 µg/mL	200 µg/mL
Kanamycin	100 µg/mL		
Chloramphenicol	34 µg/mL		
Transformation	Chemical	Electroporation	Electroporation
Wash before measuring	No	Yes	Yes

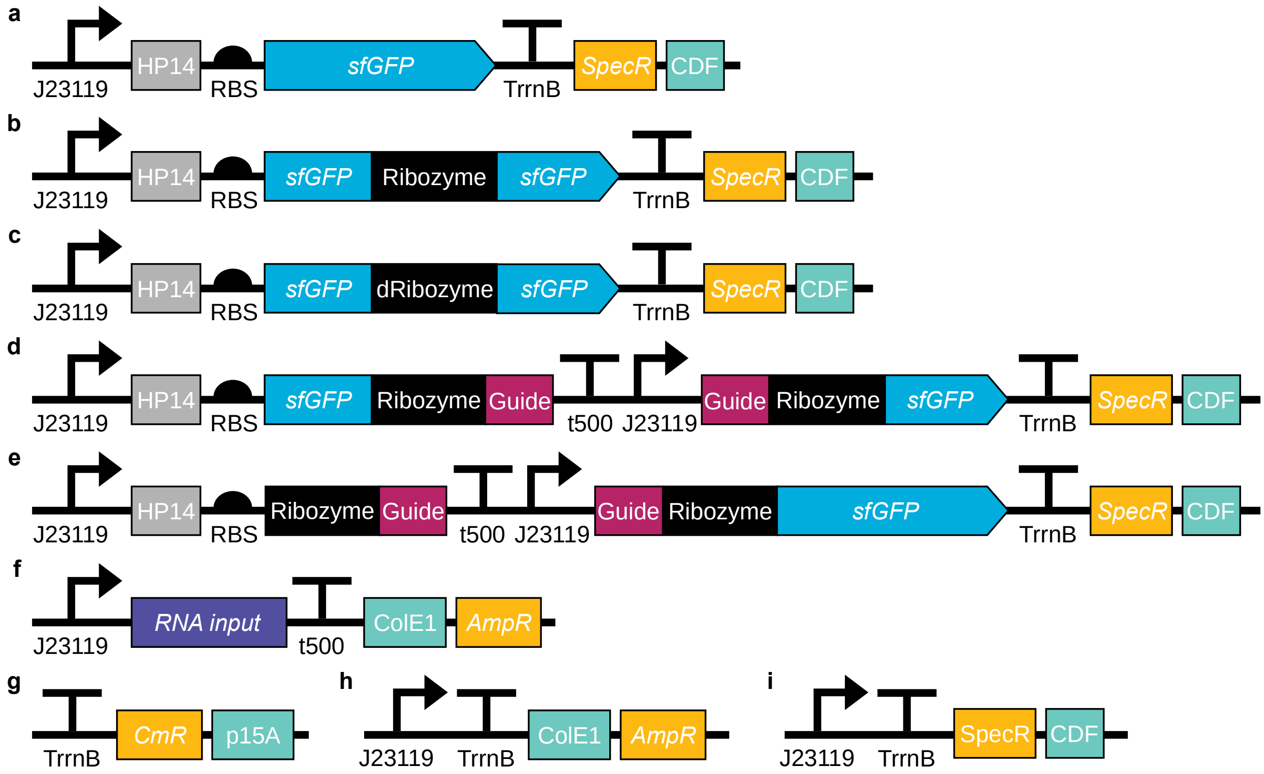

Supplementary Fig. 1. Schematic of representative DNA plasmids used in this study. (**a**) *sfGFP* expressing plasmid, (**b**) plasmid expressing the splicing ribozyme inserted within *sfGFP*, (**c**) plasmid expressing the catalytically dead splicing ribozyme within *sfGFP*, (**d**) plasmid expressing the split-output, RENDR-GFP system, (**e**) plasmid expressing the modular RENDR-GFP system, (**f**) plasmid expressing the RNA input, (**g**) Empty control plasmid for *CmR* p15A vectors, (**h**) Empty control plasmid for AmpR ColE1 vectors, (**i**) Empty control plasmid for *SpecR* CDF vectors.

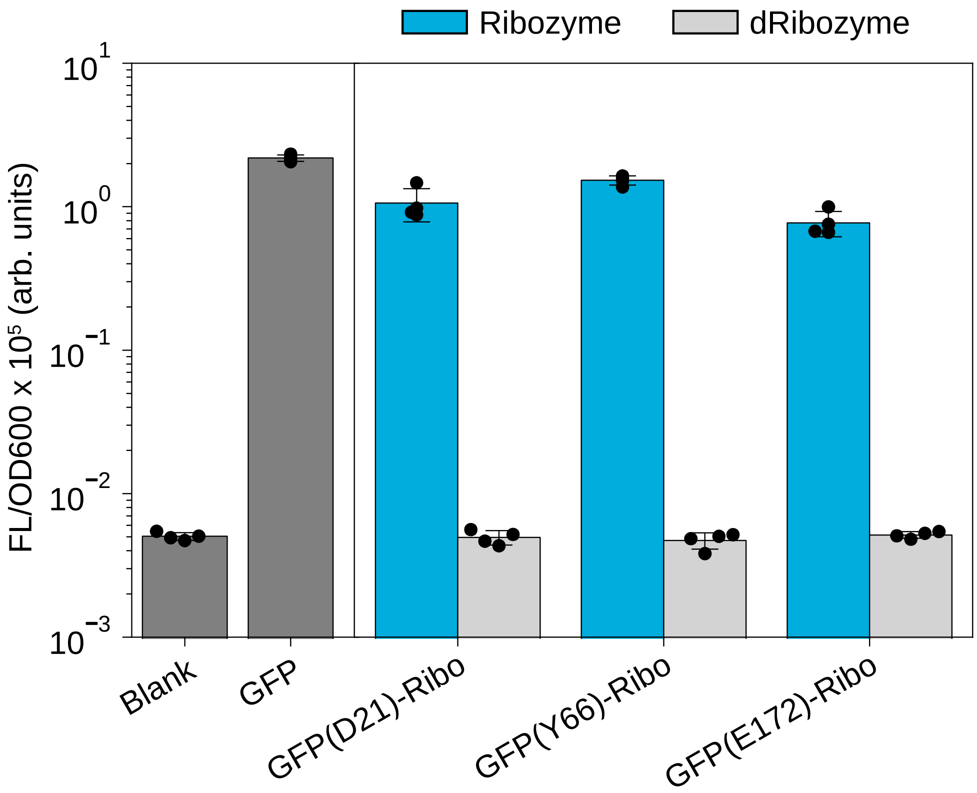

Supplementary Fig. 2. Identifying functional ribozyme insertion sites in *sfGFP*. Fluorescence characterization of ribozyme-inserted sfGFP variants (measured in units of fluorescence [FL]/optical density [OD] at 600 nm) was performed with *E. coli* transformed with an empty plasmid (Blank), a constitutively expressed sfGFP plasmid (GFP), and plasmids encoding the splicing ribozyme inserted within sfGFP at amino acid positions: D21 (GFP(D21)-Ribo), Y66 (GFP(Y66)-Ribo), and E172 (GFP(E172)-Ribo). Two variants of the ribozyme were used: a catalytically-active ribozyme (Ribozyme) and a catalytically-dead G264A mutant ribozyme (dRibozyme). Bars represent the mean values and error bars represent s.d. of n=4 biological replicates.

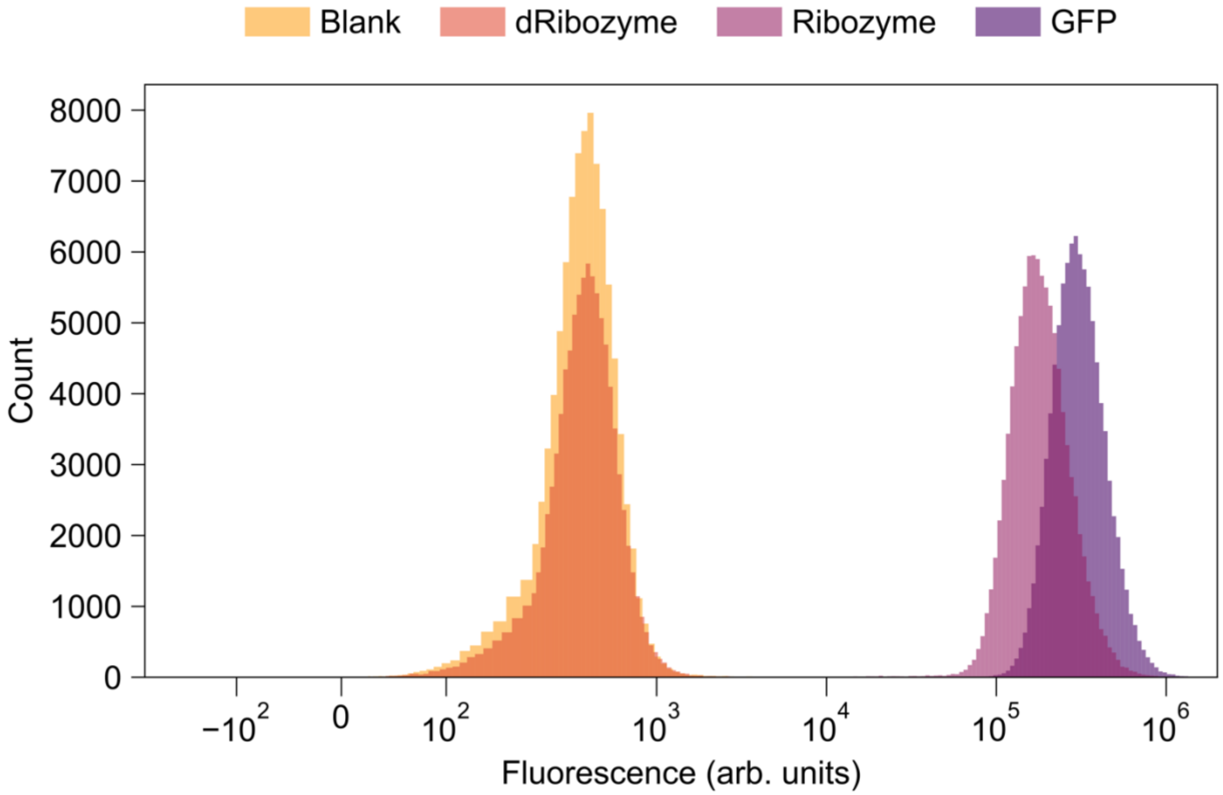

Supplementary Fig. 3. Single cell fluorescence characterization of fluorescence-based splicing assay. Single cell sfGFP fluorescence values of blank *E. coli* transformed with an empty control plasmid (Blank), a catalytically dead G264A mutant splicing ribozyme inserted after the first nucleotide of amino acid Y66 of sfGFP (dRibozyme), a catalytically active splicing ribozyme inserted after the first nucleotide of amino acid Y66 of GFP (Ribozyme), and an sfGFP positive control (GFP). Values measured in units of arbitrary fluorescence (arb. units). Data shows a histogram of $n = 3$ biological replicates plotted on a 'symlog' axis with a linear threshold of 1.8×10^2. The gating strategy used is described in **Supplementary Figure 21**.

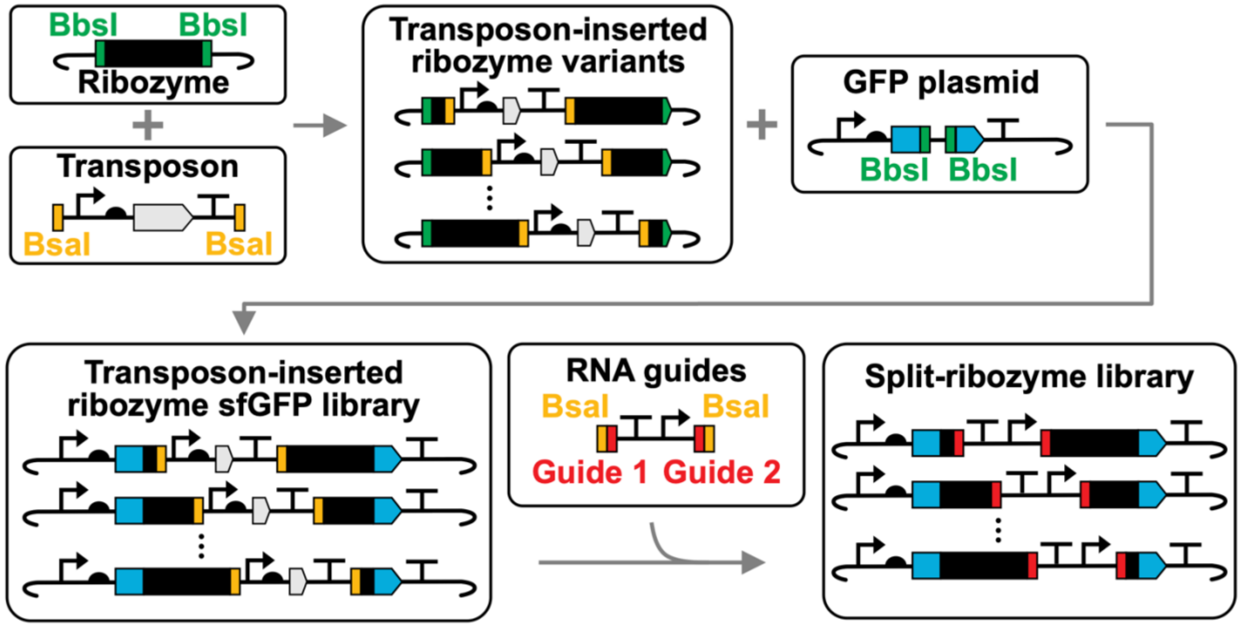

Supplementary Fig. 4. Schematic of split-ribozyme library generation using transposon mutagenesis. To perform *in vitro* transposon mutagenesis a synthetic transposon containing a kanamycin resistance cassette was isolated through restriction digestion and purification, and then randomly inserted into a ribozyme plasmid using Mu transposase. The subsequent transposon-inserted ribozyme variants were digested, gel purified, and cloned into a plasmid containing *sfGFP*. Finally, the synthetic transposon was replaced with a cassette containing RNA guide sequences, yielding the split-ribozyme library.

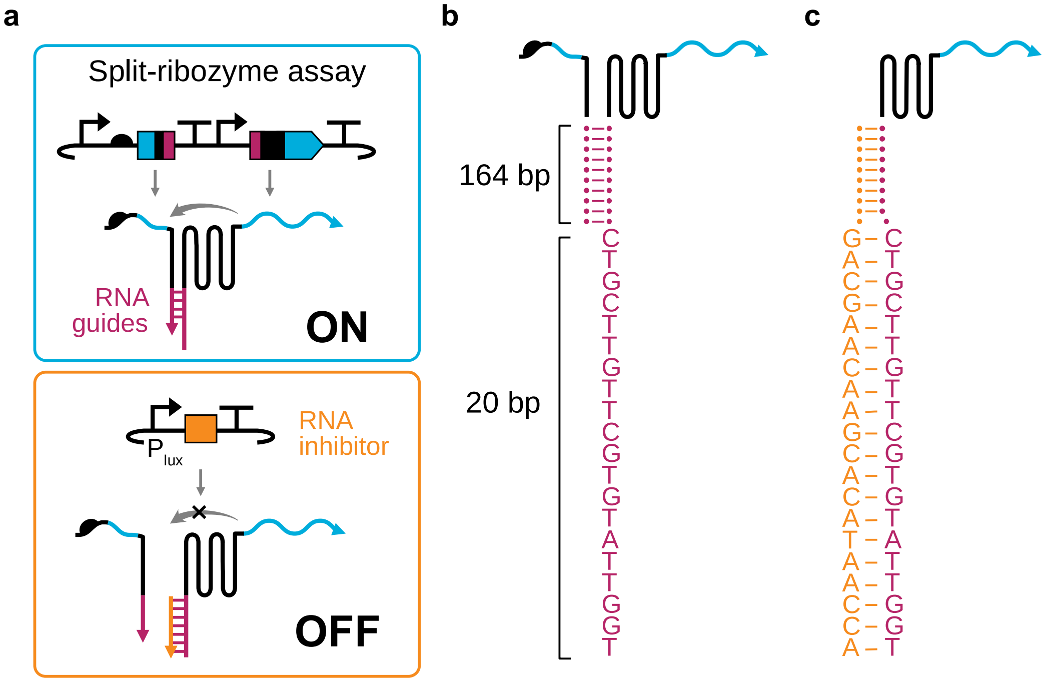

Supplementary Fig. 5. Schematic of RNA inhibitor. (**a**) Schematic of high-throughput screening approach. A split-ribozyme assay is used to determine splicing from each split ribozyme in the absence (off) and presence (on) of additional stabilizing RNA interactions. RNA guides (magenta) are used to facilitate RNA interactions between ribozyme fragments, which are blocked with an RNA inhibitor (orange). (**b**) Detailed schematic of the RNA guide sequence composition. The 5' ribozyme fragment contains a guide RNA that is 164 nt long and the 3' ribozyme fragment contains a guide RNA that is 184 nt long, and contains an additional toehold sequence (20 bp long). (**c**) The RNA inhibitor is designed to bind the 3' ribozyme fragment RNA guide and the additional toehold sequence.

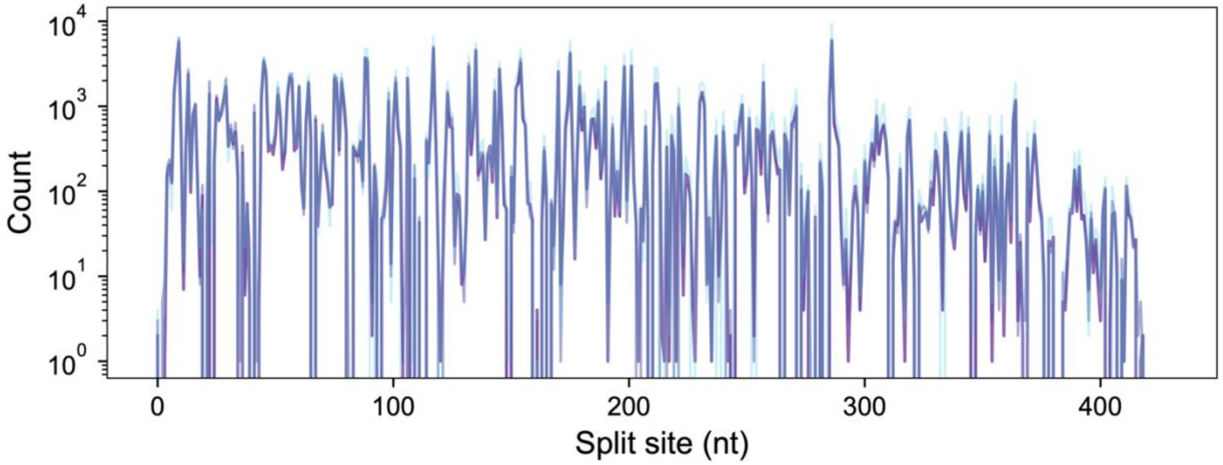

Supplementary Fig. 6. Diversity of the split-ribozyme library. Abundance of different ribozyme split sites within the split-ribozyme library. NGS was performed on the split-ribozyme library and the read counts processed to identify split sites. Read counts were measured from three technical replicates (blue, light purple, dark purple). This library had 93.6% coverage across all possible split sites, with 392 of 419 possible split sites present.

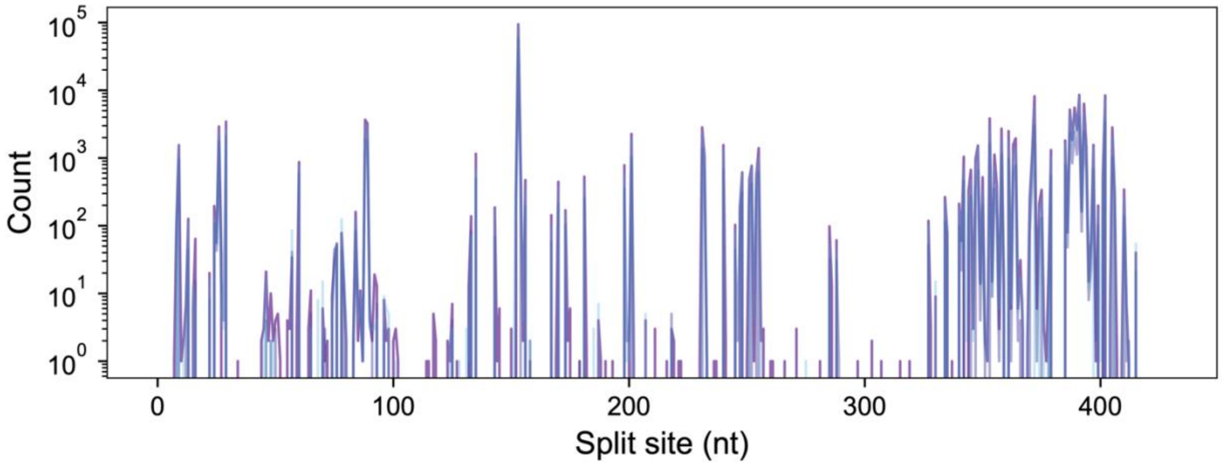

Supplementary Fig. 7. FACS of the split-ribozyme library results in enrichment at specific split sites. Abundance of ribozyme split sites within the split-ribozyme library following FACS. NGS was performed on the sorted split-ribozyme library and the read counts processed to identify split sites. Read counts were measured from three technical replicates (blue, light purple, dark purple). After sorting, coverage was 51.3% across all splits, with 215 of 419 possible split sites present.

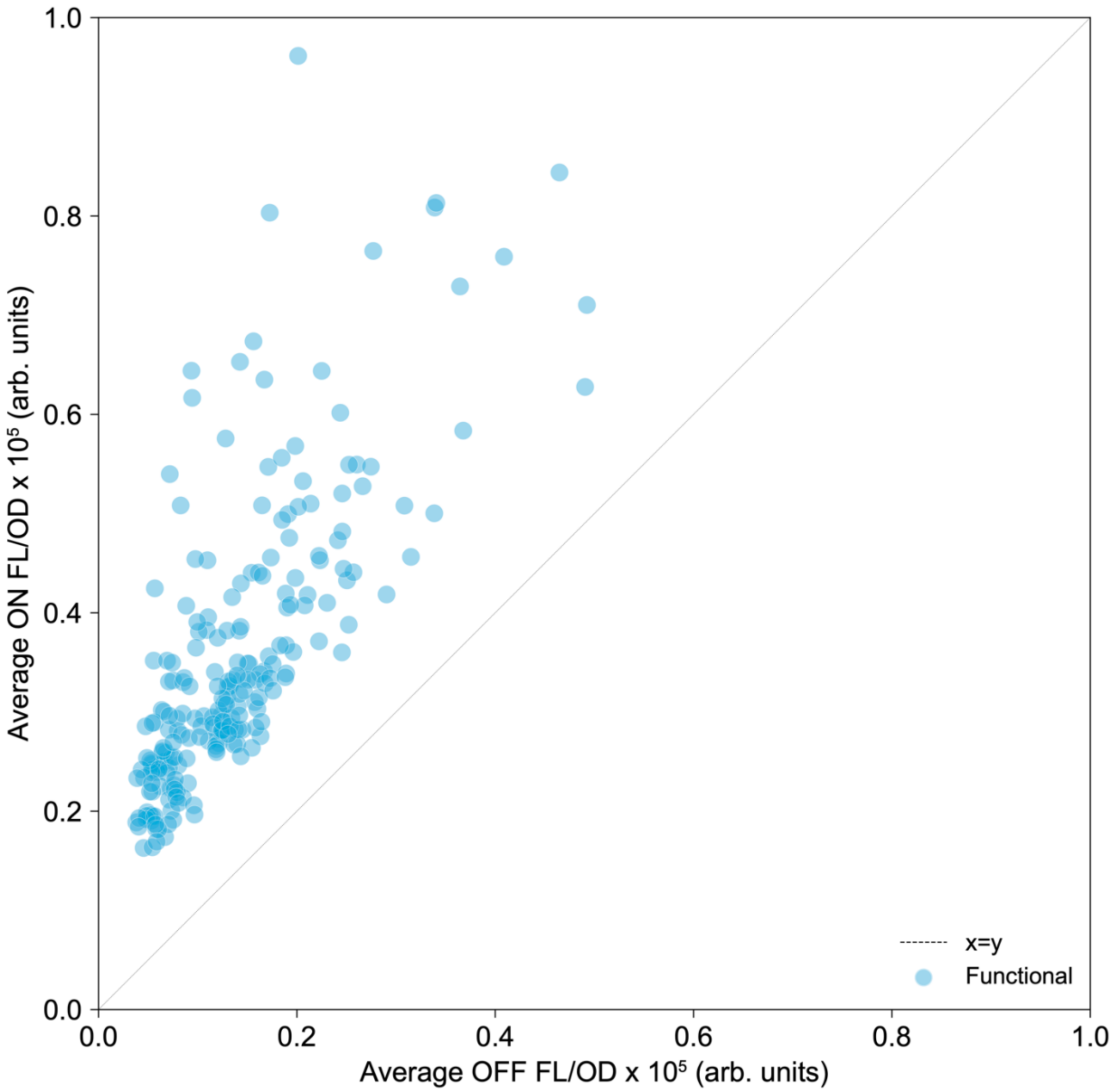

Supplementary Fig. 8. Identification of functional split ribozymes. Fluorescence characterization of individual split ribozyme variants from the sorted library. Fluorescence characterization (measured in units of fluorescence [FL]/optical density [OD] at 600 nm) was performed with *E. coli* transformed with plasmids encoding different split-ribozyme variants and a plasmid encoding the RNA inhibitor under the control of an AHL-inducible promoter. Fluorescence was characterized in the presence (OFF) and absence (ON) of 1 µM AHL. Functional split variants (ON>OFF) were binned based on their distance from the x=y line. 225 split variants are shown and data shows mean of n = 2 biological replicates.

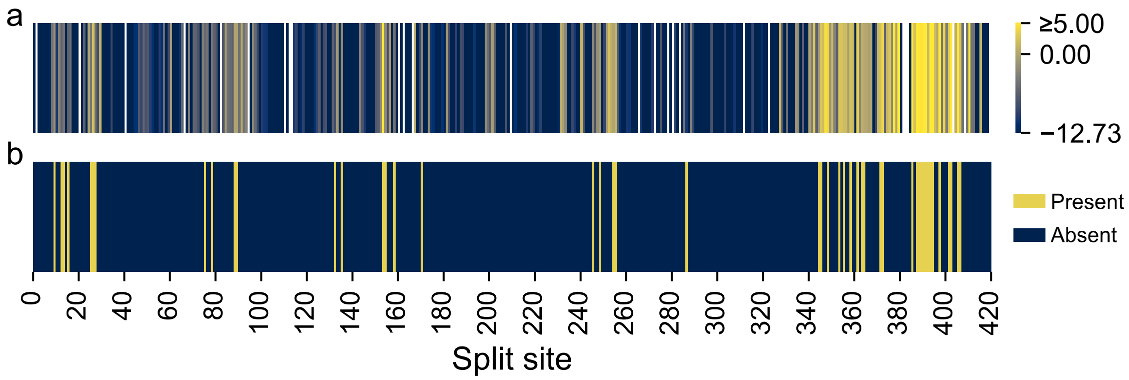

Supplementary Fig. 9. Comparison of split variants identified through FACS-seq and individual colony screening. (a) Average relative enrichment of split variants along the ribozyme from FACS-seq experiments (data from **Fig. 2B**). Relative enrichment values calculated from NGS of three technical replicates. **(b)** Functional split sites were identified from individual colony screening of sorted ribozyme variants (**Supplementary Fig. 8**). Validated functional sites are yellow (Present) and those that were not present (i.e., not picked as colonies or not identified as highly functional) are blue (Absent).

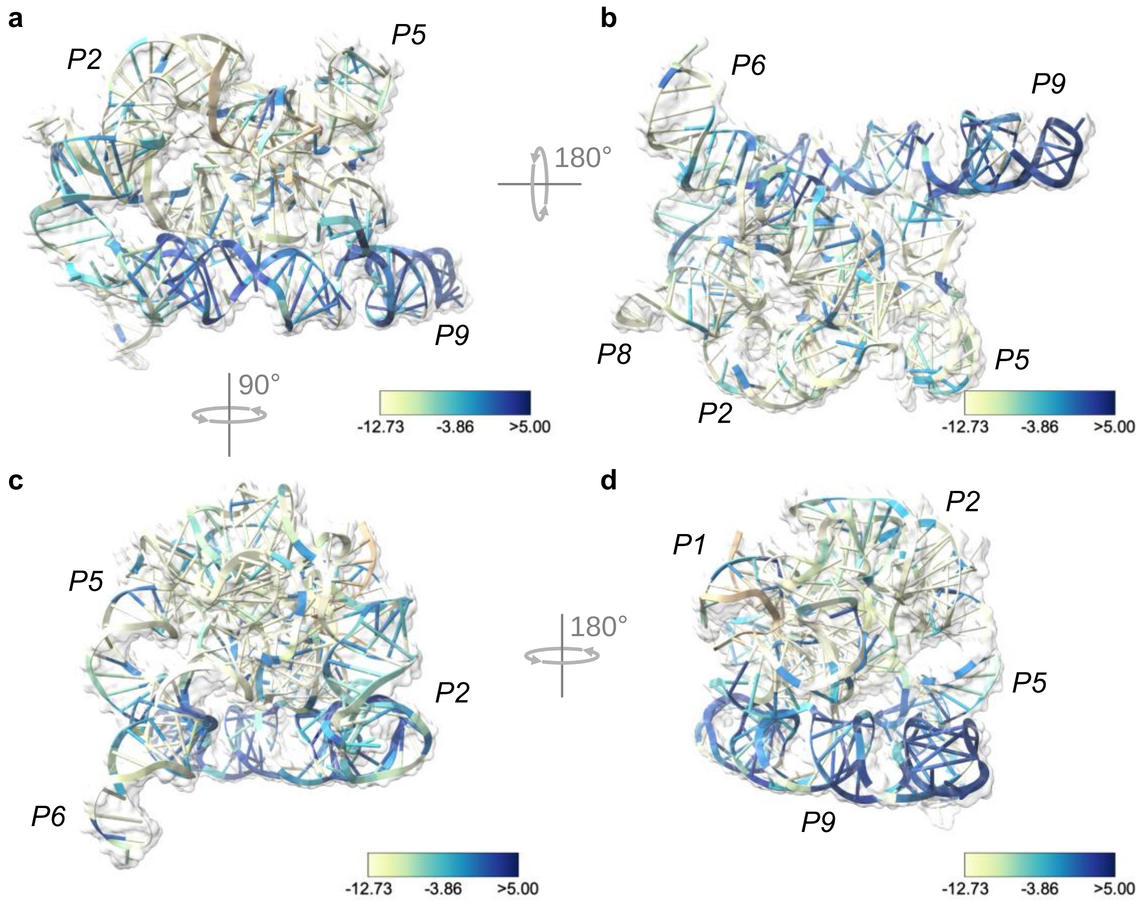

Supplementary Fig. 10. Three-dimensional structure shows functional ribozyme split sites in surface accessible regions. Relative enrichment of each split site overlayed onto the tertiary structure of the *Tetrahymena thermophila* ribozyme (PDB accession code: 7EZ2 [https://www.rcsb.org/structure/7EZ2])[1], at angles showing (**a**) the P9 domain wrapping around the structure, (**b**) the structure in panel (a) turned 180° along the x-axis with P2, P5, P6, P8, and P9 domains annotated (**c**) the structure in panel (a) turned 90° counterclockwise along the y-axis with the P2, P5, and P6 domains annotated, and (**d**) the structure in panel (c) turned 180° along the y-axis with the P1, P2, P5, and P9 domains annotated. Beige strands are the substrate sequences. Split sites not present in the initial library are colored in white.

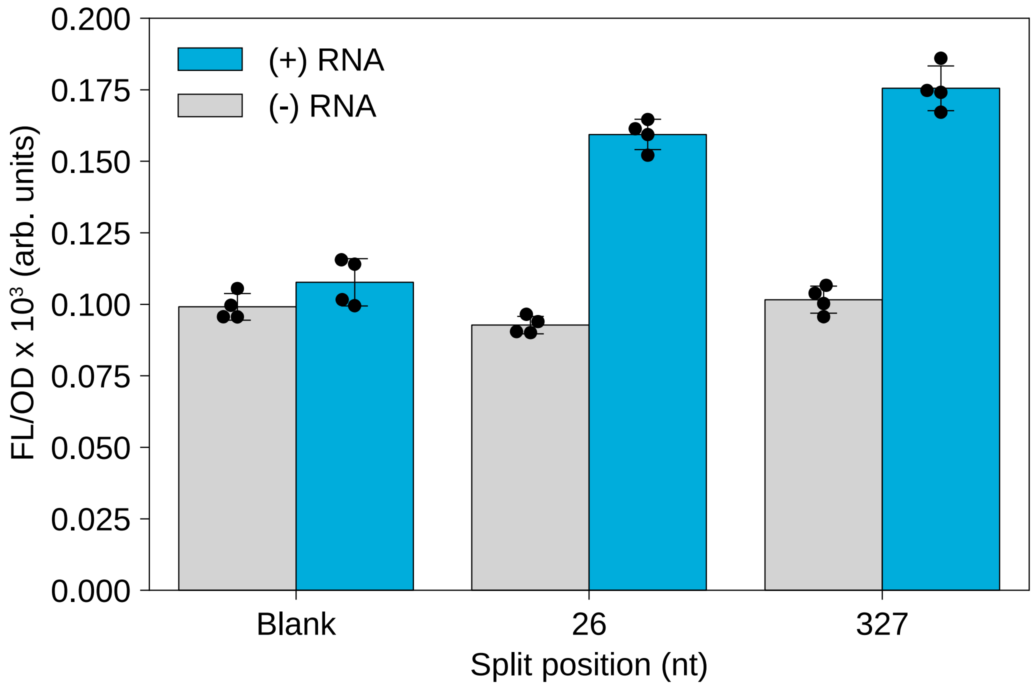

Supplementary Fig. 11. Two enriched split sites resulted in RENDR variants with low-fold activation. RENDR variants with split site 26 and 327 showed low (<2-fold) activation of sfGFP expression in the presence of a constitutively expressed RNA input (blue, (+) RNA) relative to these RENDR variants without RNA input (grey, (-) RNA). Fluorescence characterization (measured in units of fluorescence [FL]/optical density [OD] at 600 nm) was performed on *E. coli* co-transformed with the RENDR plasmid and either an empty plasmid or a plasmid constitutively expressing the RNA input. Autofluorescence of *E. coli* was determined using cells transformed with empty plasmids (blank). Bars show mean values and error bars represent s.d. of $n = 4$ biological replicates shown as points.

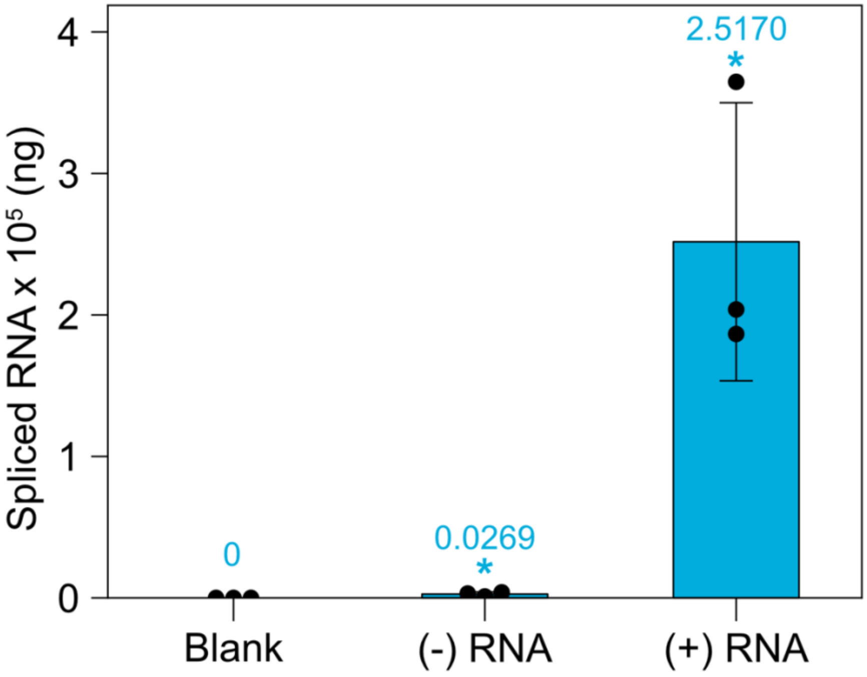

Supplementary Fig. 12. Reverse transcription quantitative PCR (RT-qPCR) of RENDR spliced output in the presence and absence of RNA input. Spliced RNA products from RENDR were measured using RT-qPCR using relative normalization to a standard curve (ng). Measurements were performed on total RNA extracted from *E. coli* co-transformed with the RENDR plasmid and either an empty plasmid ((-) RNA) or a plasmid constitutively expressing the RNA input ((+) RNA). *E. coli* cells transformed with empty plasmids (blank) were used as a negative control. Bars show the mean and error bars show the s.d. of n = 3 biological replicates. Mean values are shown above each bar. Significance was determined using a two-tailed t-test (alpha=0.05), which resulted in p-values of 0.036 and 0.011 for (-) RNA and (+) RNA relative to Blank, respectively. Statistically significant differences ($p < 0.05$) are indicated by asterisks above bars.

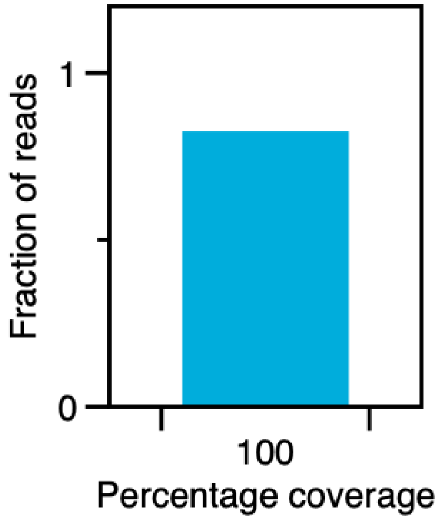

Supplementary Fig. 13. Amplicon sequencing of RENDR spliced output. Alignment of NGS reads of RENDR spliced output from *E.coli* cells. Alignments used a 20 nucleotide sequence that is the expected product surrounding the uracil splice site. The majority of NGS reads showed perfect alignment (~83%) to the expected product.

.

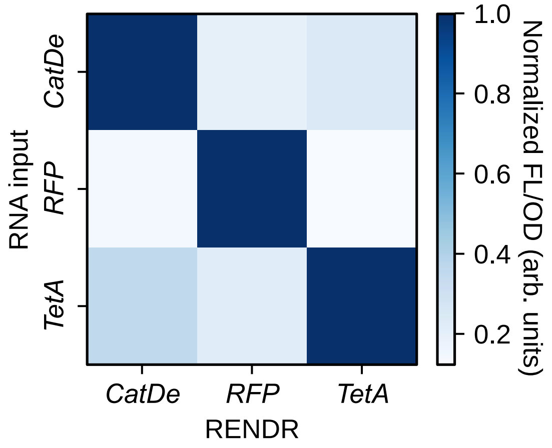

Supplementary Fig. 14. RENDR variants are specifically activated by their cognate RNA inputs. Orthogonality matrix showing RENDR variants on the x-axis and their response to different RNA input sequences on the y-axis. RENDR constructs containing RNA guides that target sequences from catechol 2,3-diooxygenase (*CatDe*), red fluorescent protein (*RFP*), and tetracycline resistant protein (*TetA*) were co-transformed into *E. coli* with each of the three constitutively expressed cognate and non-cognate RNA inputs. Fluorescence output was measured in units of fluorescence [FL]/optical density [OD] at 600 nm and values for each RENDR variant were normalized to the output of the cognate RENDR-RNA input pair. Data shown are the mean values of $n = 4$ biological replicates.

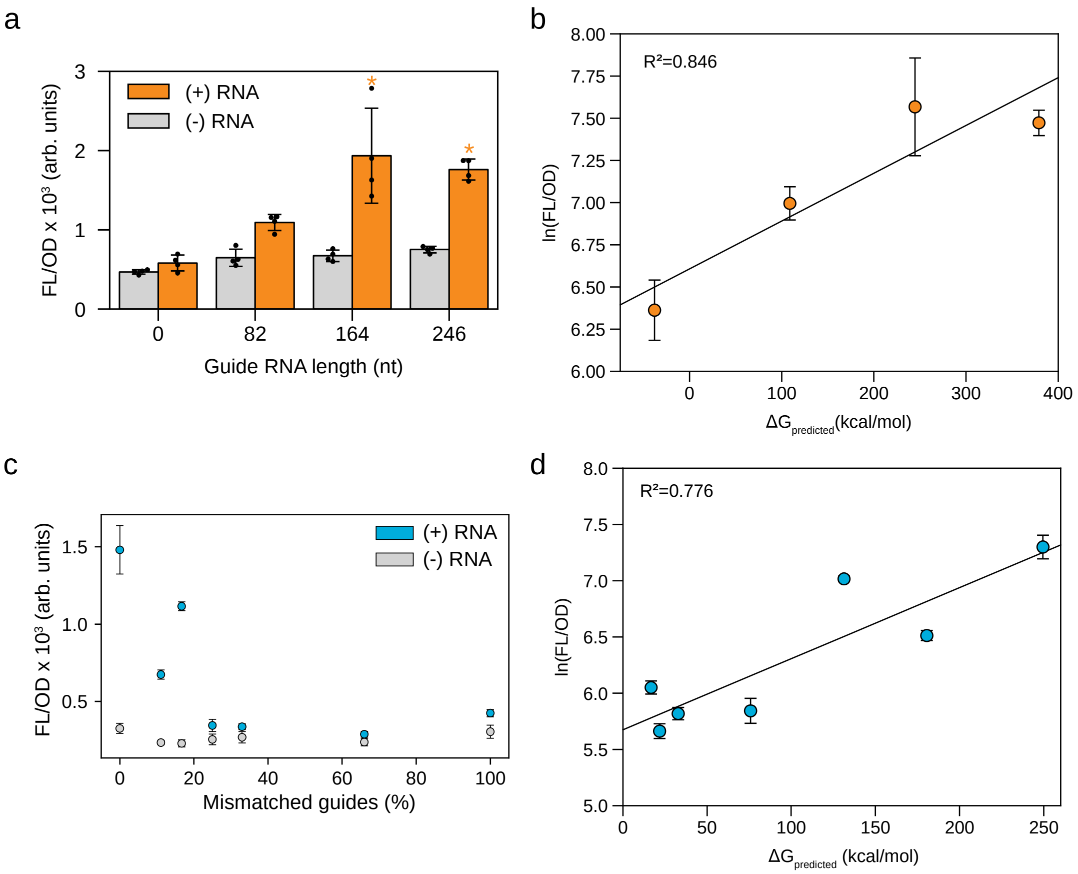

Supplementary Fig. 15. RNA guide length influences RENDR output levels and can be used as a predictive parameter. (**a**) Fluorescence characterization of RENDR with varying RNA guide lengths targeting a constant length RNA input. Fluorescence characterization (measured in units of fluorescence [FL]/optical density [OD] at 600 nm) was performed on *E. coli* co-transformed with each RENDR design and a plasmid containing an arabinose-inducible RNA input sequence, where cells were subjected to 1% arabinose (orange) or no arabinose (grey). Autofluorescence of *E. coli* was determined using cells transformed with empty plasmids (blank). Significance was determined using a two-tailed t-test (alpha=0.05), which resulted in p-values of 0.78, 0.074, 5.76e-3, and 6.46e-6 for (+) RNA samples of guide length 0, 82, 164, and 246 relative to (-) RNA, respectively. Statistically significant differences ($p < 0.05$) are indicated by asterisks above bars. (**b**) Observed correlations between the fluorescence characterization of protein output (natural log FL/OD) and $\Delta G_{Predicted}$ of different RNA guide lengths. Coefficient of determination (R^2) is displayed in the top left corner. (**c**) Fluorescence characterization of RENDR with RNA guide of constant length containing different percentages of mismatched base pairs to an RNA input. Fluorescence characterization (measured in units of fluorescence [FL]/optical density [OD] at 600 nm) was performed on *E. coli* co-transformed with each RENDR design and plasmid containing an arabinose-inducible RNA input, where cells were subjected to 1% arabinose (blue) or no arabinose (grey). Autofluorescence of *E. coli* was determined using cells transformed with empty plasmids (blank). (**d**) Observed correlations between the fluorescence characterization of protein output (natural log FL/OD) and $\Delta G_{Predicted}$ of RNA guide with different percentages of mismatched base pairs. Coefficient of determination (R^2) is displayed in the top left corner. Bars in (**a**) and points in (**b**, **c,** and **d**) represent mean values and error bars represent s.d. of n = 4 biological replicates shown as points in (**a**), with one exception where n=3 (induced, 16.7% mismatch).

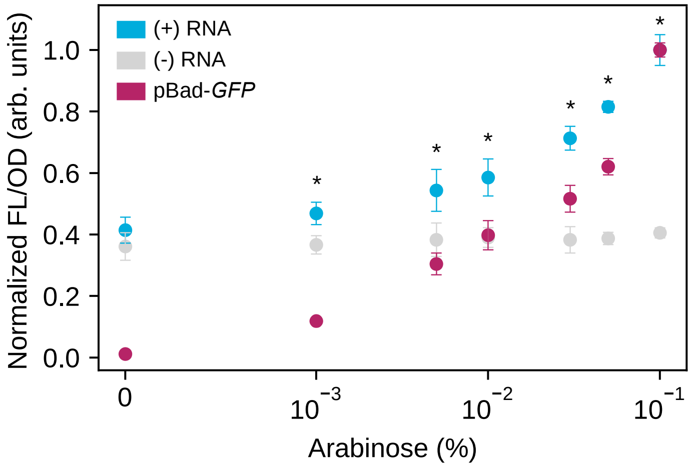

Supplementary Fig. 16. Dose-response curve of RENDR. GFP output from cells transformed with RENDR was measured in response to varying levels of RNA input that was controlled using an arabinose inducible (pbad) promoter ((+) RNA). As a control, output from cells transformed with RENDR with the pbad-RNA absent ((-) RNA) was measured in parallel. Fluorescence characterization (measured in units of fluorescence [FL]/optical density [OD] at 600 nm) was performed and the data normalized to the maximum RENDR output at 0.1% arabinose. In parallel, the transcriptional output from the pbad promoter was characterized using a GFP reporter (pbad-*GFP*) and this data was normalized to the maximum promoter output at 0.1% arabinose. Data points show mean values and error bars represent s.d. of $n = 4$ biological replicates shown as points. Significance was determined using a two-tailed t-test (alpha=0.05), which resulted in p-values of 0.135, 4.8e-3, 0.01, 1.22e-3, 2.67e-5, 6.98e-8, and 4.97e-7 for (+) RNA samples with Arabinose (%) = 0, 0.001, 0.005, 0.01, 0.03, 0.05, 0.1 relative to (-) RNA, respectively. Statistically significant differences ($p < 0.05$) are indicated by asterisks above points.

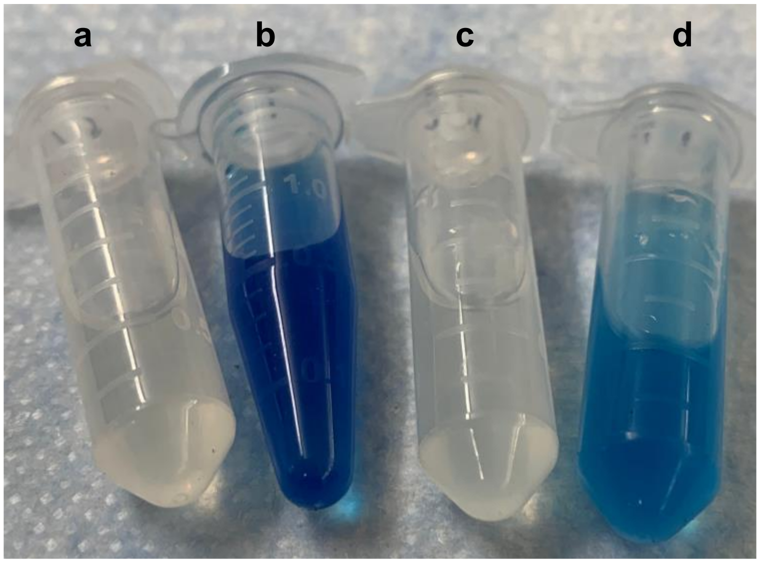

Supplementary Fig. 17. RENDR variant using a flavin-containing monooxygenase (FMO) output produces visually discernable indigo levels upon detection of RNA. Photograph of DMSO-extracted indigo obtained from *E. coli* cultures transformed with (**a**) an empty plasmid, (**b**) a constitutively expressed ribozyme-*FMO* output, (**c**) a RENDR-FMO plasmid with an empty plasmid, and (**d**) a RENDR-FMO plasmid with a plasmid expressing the RNA input.

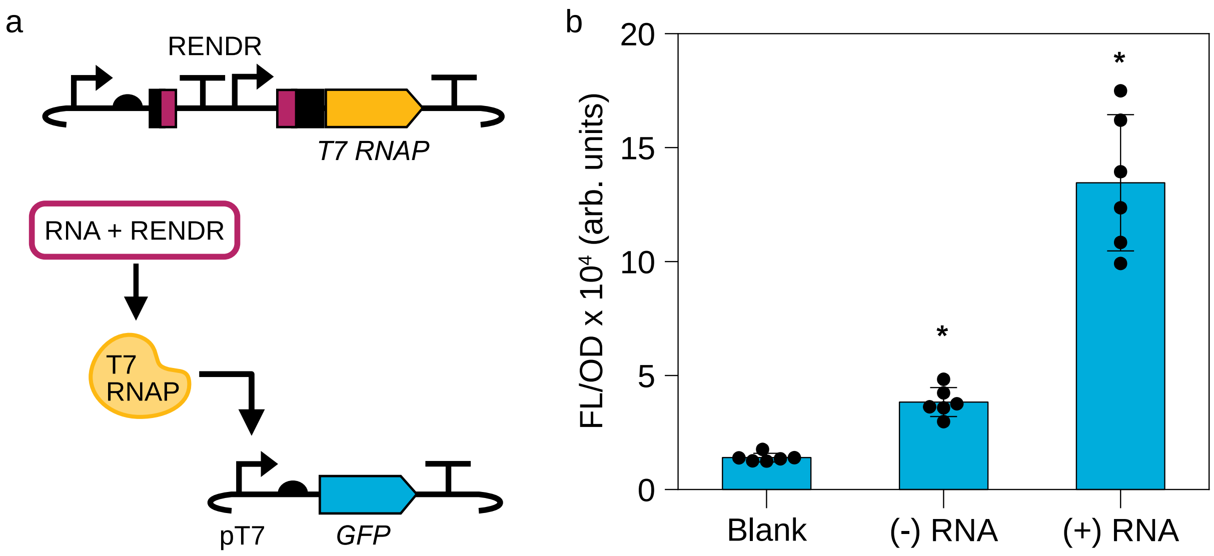

Supplementary Fig. 18. RENDR can couple the production of T7 RNAP to an RNA input. (**a**) Schematic of RENDR activating the expression of T7 RNAP in response to an RNA input. T7 RNAP activates the expression of *sfGFP* through T7 promoter binding (**b**) Fluorescence characterization (measured in units of fluorescence [FL]/optical density [OD] at 600 nm) was performed with *E. coli* transformed with plasmids encoding for empty plasmids (Blank), RENDR-T7RNAP and a T7-inducible promoter-driven *GFP* ((-) RNA), and the (-) RNA plasmids with a plasmid encoding the RNA input ((+) RNA). Bars in (**b**) represent mean values and error bars represent s.d. of n = 6 biological replicates shown as points. Significance was determined using a two-tailed t-test (alpha=0.05), which resulted in p-values of 3.99e-6 and 1.8e-6 for (-) RNA and (+) RNA relative to Blank, respectively. Statistically significant differences ($p < 0.05$) are indicated by asterisks above bars.

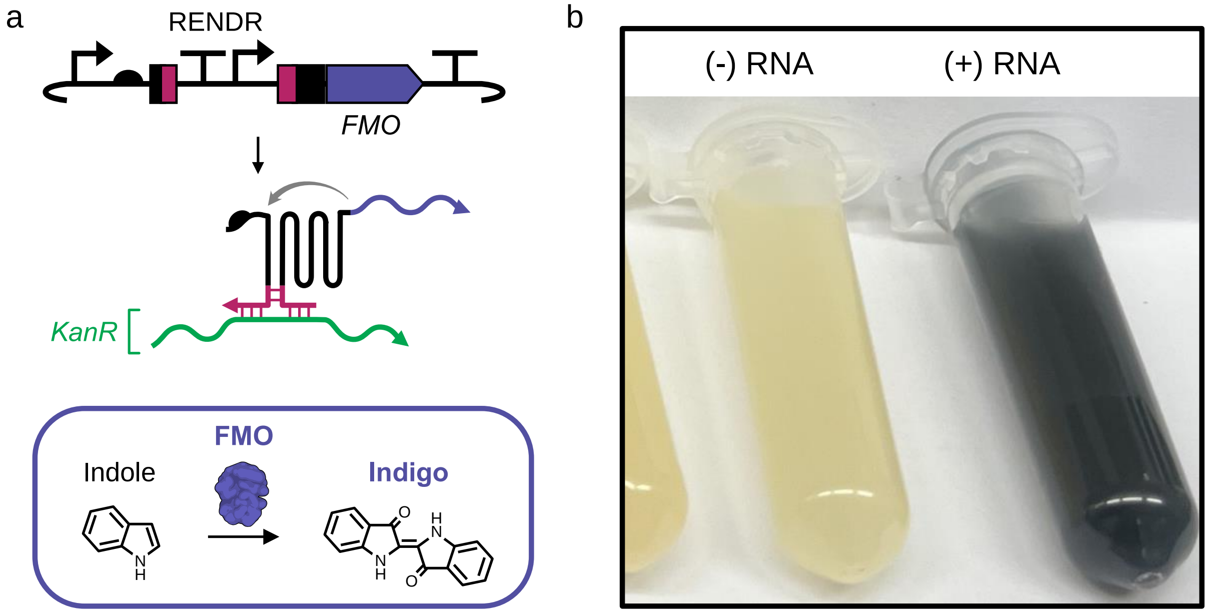

Supplementary Fig. 19. Cell culture turns blue in response to the presence of antibiotic resistance. (**a**) Schematic of RENDR-FMO activating in the presence of *KanR* mRNA and FMO converting indole into the visually-identifiable indigo. (**b**) Cells and indigo were collected from 5 mL of culture after 48 hrs of growth. Cultures contain cells transformed with a plasmid encoding RENDR-FMO and either an empty vector plasmid ((-) RNA) or a plasmid encoding the constitutively-expressed *kanR* mRNA ((+) RNA).

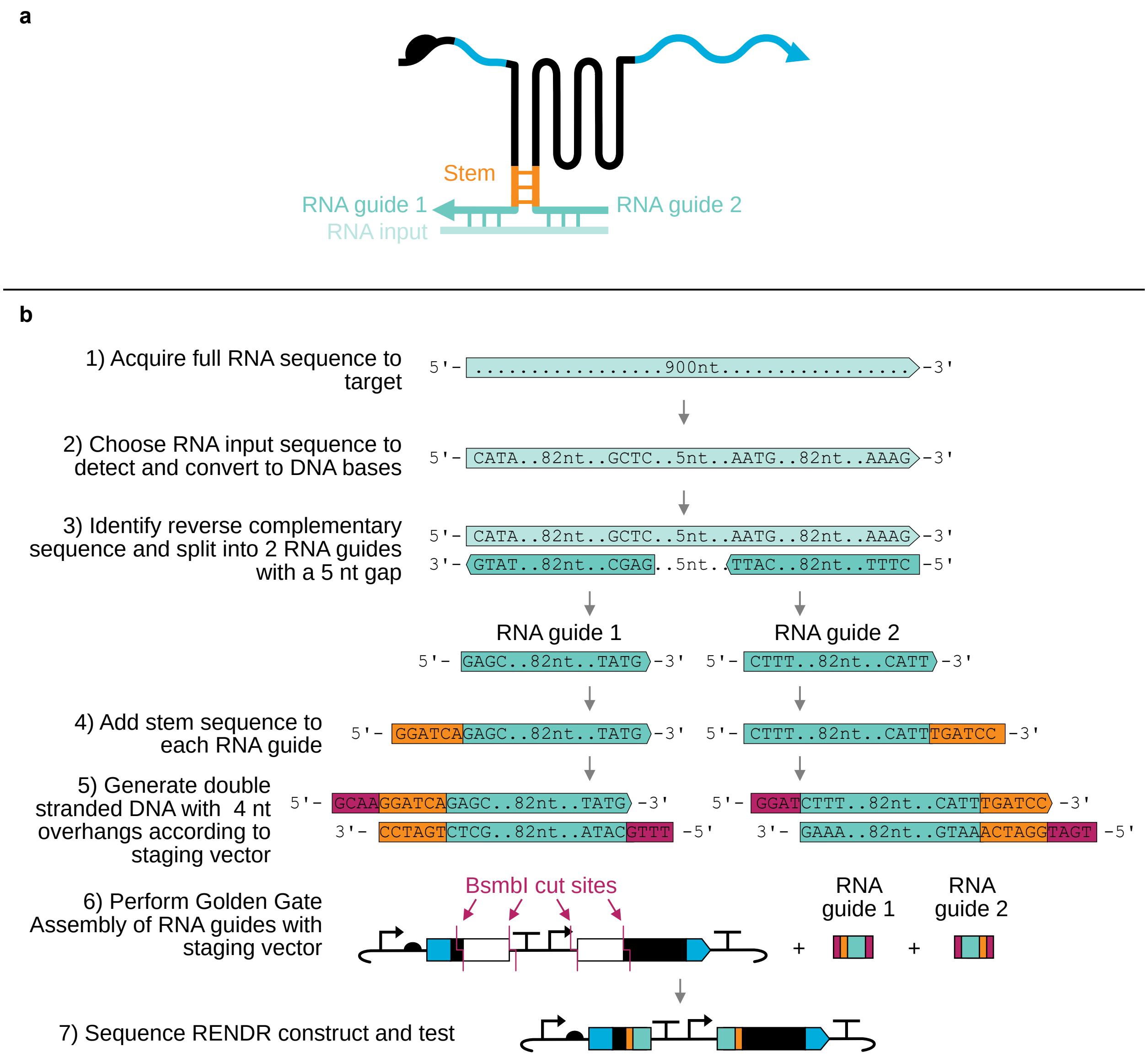

Supplementary Fig. 20. Schematic of RENDR design and cloning. Figure shows an overview of the final RENDR system (**a**) and the steps required to design and clone RENDR variants (**b**). A detailed description of the design and cloning approach is provided in **Supplementary Note 1**.

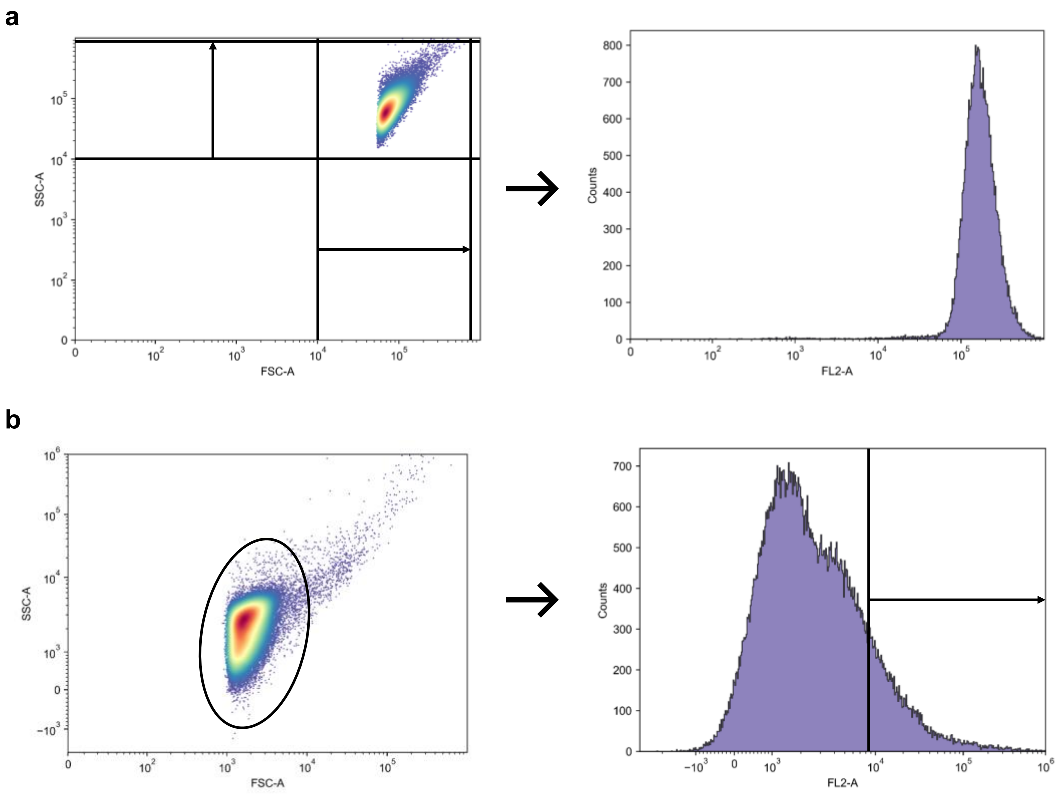

Supplementary Figure 21. Gating strategy for flow cytometry experiments. (**a**) For the flow cytometry experiment, which is shown in **Supplementary Figure 3**, samples were gated using two linear gates - one horizontal and one vertical - on a chart plotting the forward scatter (FSC, x-axis) values against the side scatter (SSC, y-axis) values in an effort to capture the entire distribution of cells. Gated samples were then plotted as histograms, with GFP fluorescence (FL2-A) on the x-axis and the cell count on the y-axis. (**b**) In the FACS experiment, which data is shown in **Figure 2a, 2b, and in Supplementary Figures 6, 7, 9, and 10**, cells run through the flow cytometer were initially gated on an FSC vs SSC plot using an ellipse shaped gate to capture the densest region. Gated cells were then plotted on a histogram showing GFP fluorescence (FL2-A). In sorting rounds where cells were uninduced, we expected functional split-ribozyme variants to be present in the region with high fluorescence, and therefore used a linear gate to sort the most highly fluorescent cells (top 14% of the population). In sorting rounds where cells were induced, we gated cells in the same way, except that the linear gate on the histogram was set to capture the lowly fluorescent cells (bottom 85% of the population).

Supplementary Note 1. Designing RENDR for the detection of a new RNA input

To enable users to create new RENDR systems we have created a golden-gate staging vector (JEC847) and provide a description of how to use this plasmid. As shown in **Supplementary Fig. 20**, each RENDR system contains two unique RNA guides that bind to corresponding sequences that are 5 nt apart in an RNA input. To begin the design of a new RENDR variant, the following steps should be followed: (1) acquire the RNA input to be detected, (2) identify the specific region of the RNA to be sensed and convert to DNA, and (3) identify RNA guide sequences that are the reverse complement of the target sequence and separated by a 5 nt gap. (4) Add a 6 nt sequence that will encode a stem between the guide and the ribozyme sequence that is used to enhance split-ribozyme complementation. (5) Generate double-stranded DNA that contains the 5' overhangs complementary to the golden gate staging vector. We generate these by either annealing oligonucleotides or adding golden gate sites by PCR. (6) Add the DNA fragments encoding each RNA guide along with the golden gate staging vector (JEC847) and perform a golden gate reaction with the BsmbI enzyme. (7) Transform reactions, isolate DNA plasmids, and sequence to validate the construct.

Supplementary Note 2. Describing RENDR design principles with a thermodynamic model.

Our goal was to establish design rules for the RENDR input detection reaction by linking the sequences of the RNA guide and RNA input to the observed output levels. We began with the consideration that by splitting the ribozyme into two halves, neither fragment alone would be catalytically active (*i.e.,* able to produce the spliced RNA output). However, in the presence of the RNA input, interactions between the RNA guides attached to each half of the ribozyme and the RNA input form, promoting complementation of the split ribozyme to form the catalytically active structure. Since the RNA guides are fully complementary to the RNA input, an extended duplex (ED) can form between these species regardless of their length. In our experimental data (**Fig. 3b** and **Supplementary Fig. 15**) we observed that increasing the length, and hence the strength, of interaction in the ED resulted in proportional increases in splicing. Using this observation, we predicted that the rate of splicing is directly related to the rate of ED formation, which we reasoned could be captured in a sequence-function thermodynamic model.

To begin with, we assume the rate of splicing (k_s) is proportional to the expression of our sfGFP output, which in turn, is proportional to the observed fluorescence/optical density (FL/OD) measurements:

$$k_s \propto FL/OD \quad (1)$$

The rate of splicing (k_s) can be estimated from the activation energy barrier (E_a) for the transition of the initial states (IS) to the ED using the Arrhenius equation: (k_B = Boltzman's constant, T is temperature).

$$k_s \sim e^{-E_a/k_BT} \quad (2)$$

To estimate E_a, we can use a linear free energy relationship in which $E_a \sim \Delta G_{ED} - \Delta G_{IS}$ [2]:

$$E_a \sim \Delta G_{ED} - \Delta G_{IS} \quad (3)$$

Taking the following definition of ΔG_{IS}:

$$\Delta G_{IS} \sim \Delta G_{Guide\,1} + \Delta G_{Guide\,2} + \Delta G_{RNA\,input} \quad (4)$$

We have:

$$FL/OD \sim k_s \sim e^{-(\Delta G_{ED} - \Delta G_{Guide\,1} - \Delta G_{Guide\,2} - \Delta G_{RNA\,input})/k_BT} \quad (5)$$

Which can be simplified to:

$$ln(FL/OD) \sim (\Delta G_{Guide\,1} + \Delta G_{Guide\,2} + \Delta G_{RNA\,input} - \Delta G_{ED}) \quad (6)$$

Therefore, using this model we predict a linear relationship between the ln(FL/OD) and the difference in free energies between the IS of the RNA guides and RNA input, and the extended duplex (ED). Importantly, this model captures how intramolecular folding within each of the individual RNA species competes for folding of the ED (intermolecular interactions).

To test this model, we first calculated the individual ΔG terms. We estimated each ΔG term as follows:

- ΔG_{Guide1} and ΔG_{Guide2} = The minimum free energy (MFE) of the two RNA guide sequences attached to each fragment of RENDR. The MFE was calculated in NUPACK[3,4] with

temperature = 37 °C, material = 'rna95' free energy parameter set, ensemble = 'stacking', sodium = 0.25 M, and magnesium = 0.02 M[5].

- $\Delta G_{RNAinput}$ = The MFE of the RNA input was calculated from the RNA input (not-including the terminator hairpin). The MFE was calculated in NUPACK with temperature = 37 °C, material = 'rna95' free energy parameter set, ensemble = 'stacking', sodium = 0.25 M, and magnesium = 0.02 M.
- ΔG_{ED} = The duplex binding energy of the ED. The MFE of the ED of all three strands (Guide 1, Guide 2, and RNA input) was calculated in NUPACK with temperature = 37 °C, material = 'rna95' free energy parameter set, ensemble = 'stacking', sodium = 0.25 M, and magnesium = 0.02 M. We note that for the RNA input only the portion complementary to each guide RNA was used. This was because using the full RNA input for the ΔG_{ED} dramatically increases the NUPACK run time.

Using these free energy terms and our model, we compared a series of RENDR datasets to the predicted activity. First, we compared our $\Delta G_{predicted}$ term to experimentally measured outputs for a library of RNA guide length variants targeting an RFP mRNA (**Figure 3b** and **Figure 3d**). Second, we performed the same experiment on a distinct target RNA (**Supplementary Fig. 15a,b**). Finally, we compared our ΔG predicted term to experimentally measured outputs from a library of RNA guides that were fixed in length but contained different degrees of mismatch base pairs to the input RNA (**Supplementary Fig. 15c,d**). From all these experiments, we observed strong correlation between our predicted activity and the experimentally observed activity.

Supplementary Note 3. NGS data analysis pipeline

NGS reads processing

Forward and reverse NGS sequence reads were imported and the unique indices were used to bin reads into categories and assign directionality. For reads with at least one mutation in the index, the Hamming distance was calculated to attempt to match the mutated index with one of the indices used and thereby assign the read to the correct category. Reads that could not be conclusively assigned to an index were excluded from the dataset. Paired reads without one forward and one reverse read were also excluded. To determine the ribozyme split site, reverse reads were then aligned to reference sequences of both the ribozyme and the insertion containing the RNA guides. Briefly, we used the Biopython API[6] to perform alignments, running the Smith-Waterman local alignment algorithm with the "Nuc.4.4" substitution matrix, an open gap score of -10, and an extend gap score of -0.5. To determine where the ribozyme was split, reads were first aligned to the insertion reference sequence and reads with alignment scores below 1000 were removed along with any reads containing ambiguous alignment endpoints. The sequence following the insertion alignment endpoint, which we termed the ribozyme split sequence (RSS), was then aligned to the ribozyme reference sequence and resulting alignment scores were normalized to the length of the RSS. Reads with normalized RSS alignment scores below 4.5 were removed. Insertion sequences with RSS lengths too short to identify a unique split site, those without an RSS, and those with ambiguous split sites were also removed. Ribozyme split sites were determined from each RSS alignment.

Determining relative enrichment from FACS-seq

For each split site in the unsorted and sorted library NGS run, read counts were summed and a frequency was calculated by normalizing the read count of each split site to the total number of processed reads for that NGS run. The average frequency for technical triplicate NGS runs of the unsorted and sorted libraries was calculated and the relative enrichment of each split site was determined by dividing the average frequency of the sorted library by the average frequency of the unsorted library. Significant enrichment between the unsorted and sorted libraries was assessed through p-values using a homoscedastic two-sample t-Test with an alpha of 0.05 on the average frequencies at each split site.

References

1. Su, Z. *et al.* Cryo-EM structures of full-length Tetrahymena ribozyme at 3.1 Å resolution. *Nature* **596**, 603–607 (2021).
2. Dill, K. A. & Bromberg, S. *Molecular driving forces: statistical thermodynamics in biology, chemistry, physics, and nanoscience*. (Garland Science, 2011).
3. Fornace, M. E., Porubsky, N. J. & Pierce, N. A. A Unified Dynamic Programming Framework for the Analysis of Interacting Nucleic Acid Strands: Enhanced Models, Scalability, and Speed. *ACS Synth. Biol.* **9**, 2665–2678 (2020).
4. Dirks, R. M., Bois, J. S., Schaeffer, J. M., Winfree, E. & Pierce, N. A. Thermodynamic Analysis of Interacting Nucleic Acid Strands. *SIAM Rev.* **49**, 65–88 (2007).
5. Szatmári, D. *et al.* Intracellular ion concentrations and cation-dependent remodelling of bacterial MreB assemblies. *Sci. Rep.* **10**, 12002 (2020).
6. Cock, P. J. A. *et al.* Biopython: freely available Python tools for computational molecular biology and bioinformatics. *Bioinformatics* **25**, 1422–1423 (2009).
