# Supplementary material for: A split ribozyme that links detection of a native RNA to orthogonal protein outputs
Source: Nat Commun. 2023 Feb 1;14:543. doi: 10.1038/s41467-023-36073-3 (PMC9892565; doi:10.1038/s41467-023-36073-3)
Supplement: Supplementary file 2 — Reporting Summary [file 41467_2023_36073_MOESM2_ESM.pdf]

nature portfolio

Corresponding author(s): James Chappell

Last updated by author(s): 12/01/2023

Reporting Summary

Nature Portfolio wishes to improve the reproducibility of the work that we publish. This form provides structure for consistency and transparency in reporting. For further information on Nature Portfolio policies, see our Editorial Policies and the Editorial Policy Checklist.

Statistics

For all statistical analyses, confirm that the following items are present in the figure legend, table legend, main text, or Methods section.

n/a	Confirmed	
☐	☒	The exact sample size (n) for each experimental group/condition, given as a discrete number and unit of measurement
☐	☒	A statement on whether measurements were taken from distinct samples or whether the same sample was measured repeatedly
☐	☒	The statistical test(s) used AND whether they are one- or two-sided<br>*Only common tests should be described solely by name; describe more complex techniques in the Methods section.*
☒	☐	A description of all covariates tested
☐	☒	A description of any assumptions or corrections, such as tests of normality and adjustment for multiple comparisons
☐	☒	A full description of the statistical parameters including central tendency (e.g. means) or other basic estimates (e.g. regression coefficient) AND variation (e.g. standard deviation) or associated estimates of uncertainty (e.g. confidence intervals)
☐	☒	For null hypothesis testing, the test statistic (e.g. F, t, r) with confidence intervals, effect sizes, degrees of freedom and P value noted<br>*Give P values as exact values whenever suitable.*
☒	☐	For Bayesian analysis, information on the choice of priors and Markov chain Monte Carlo settings
☒	☐	For hierarchical and complex designs, identification of the appropriate level for tests and full reporting of outcomes
☒	☐	Estimates of effect sizes (e.g. Cohen's d, Pearson's r), indicating how they were calculated

Our web collection on statistics for biologists contains articles on many of the points above.

Software and code

Policy information about availability of computer code

Data collection	Flow cytometry and sorting data was collected using a Sony Biotechnology SH800 Cell Sorter with accompanying software. Bulk fluorescence data was collected using Tecan M1000 and the Spark Control Method Editor (v3.0) on the Tecan Spark plate reader. Data collected for cells producing Methyl Bromide was performed using Agilent MassHunter Workstation Quantitative Analysis software. For modeling, free energies were calculated using a custom script in Python (v3.9) with the NUPACK package (v4.0.0.27).
Data analysis	Flow cytometry data was analyzed using Python (v3.9) with custom scripts containing the FlowCal package (v1.3.0). Sequencing data was analyzed using a custom script written in Python (v3.9) and containing the Biopython package (v1.79).

For manuscripts utilizing custom algorithms or software that are central to the research but not yet described in published literature, software must be made available to editors and reviewers. We strongly encourage code deposition in a community repository (e.g. GitHub). See the Nature Portfolio guidelines for submitting code & software for further information.

Data

Policy information about availability of data

All manuscripts must include a data availability statement. This statement should provide the following information, where applicable:

- Accession codes, unique identifiers, or web links for publicly available datasets
- A description of any restrictions on data availability
- For clinical datasets or third party data, please ensure that the statement adheres to our policy

All data files, including original plate reader output spreadsheets, FCS files, GC-MS output files, and FastQ files, are available upon request to the corresponding

author. Additionally, we have included data source file for the main figure data.

Human research participants

Policy information about studies involving human research participants and Sex and Gender in Research.

Reporting on sex and gender	*Use the terms sex (biological attribute) and gender (shaped by social and cultural circumstances) carefully in order to avoid confusing both terms. Indicate if findings apply to only one sex or gender; describe whether sex and gender were considered in study design whether sex and/or gender was determined based on self-reporting or assigned and methods used. Provide in the source data disaggregated sex and gender data where this information has been collected, and consent has been obtained for sharing of individual-level data; provide overall numbers in this Reporting Summary. Please state if this information has not been collected. Report sex- and gender-based analyses where performed, justify reasons for lack of sex- and gender-based analysis.*
Population characteristics	*Describe the covariate-relevant population characteristics of the human research participants (e.g. age, genotypic information, past and current diagnosis and treatment categories). If you filled out the behavioural & social sciences study design questions and have nothing to add here, write "See above."*
Recruitment	*Describe how participants were recruited. Outline any potential self-selection bias or other biases that may be present and how these are likely to impact results.*
Ethics oversight	*Identify the organization(s) that approved the study protocol.*

Note that full information on the approval of the study protocol must also be provided in the manuscript.

Field-specific reporting

Please select the one below that is the best fit for your research. If you are not sure, read the appropriate sections before making your selection.

☒ Life sciences ☐ Behavioural & social sciences ☐ Ecological, evolutionary & environmental sciences

For a reference copy of the document with all sections, see nature.com/documents/nr-reporting-summary-flat.pdf

Life sciences study design

All studies must disclose on these points even when the disclosure is negative.

Sample size	All data had at least 3 biological replicates (n>=3) in order to reduce the chances that results are due to variability or external factors.
Data exclusions	For all data, on rare occasions a sample failed to grow which was excluded based upon applying a threshold for OD 600 measurements.
Replication	Most plate reader experiments were performed at least 3 times across multiple days and they validated the results shown in the paper. Technical triplicates were performed for sequencing of the split-ribozyme library to reduce variability due to amplification of the sorted/ unsorted libraries.
Randomization	For induction experiments, each sample was grown as a single pre-culture and separated into identical, well-mixed experimental cultures representing (+) inducer and (-) inducer conditions.
Blinding	No blinding was needed as our experiments contained no potential for participant bias.

Reporting for specific materials, systems and methods

We require information from authors about some types of materials, experimental systems and methods used in many studies. Here, indicate whether each material, system or method listed is relevant to your study. If you are not sure if a list item applies to your research, read the appropriate section before selecting a response.

Materials & experimental systems

n/a	Involved in the study
[x]	[ ] Antibodies
[x]	[ ] Eukaryotic cell lines
[x]	[ ] Palaeontology and archaeology
[x]	[ ] Animals and other organisms
[x]	[ ] Clinical data
[x]	[ ] Dual use research of concern

Methods

n/a	Involved in the study
[x]	[ ] ChIP-seq
[ ]	[x] Flow cytometry
[x]	[ ] MRI-based neuroimaging

Flow Cytometry

Plots

Confirm that:

- [ ] The axis labels state the marker and fluorochrome used (e.g. CD4-FITC).
- [x] The axis scales are clearly visible. Include numbers along axes only for bottom left plot of group (a 'group' is an analysis of identical markers).
- [x] All plots are contour plots with outliers or pseudocolor plots.
- [x] A numerical value for number of cells or percentage (with statistics) is provided.

Methodology

Sample preparation	All cell samples were derived from transformation of DNA plasmids into bacterial cells and subsequent growth in suitable media.
Instrument	Sony Biotechnology SH800 Cell Sorter
Software	Accompanying software for Sony Biotechnology SH800 Cell Sorter and custom Python scripts using FlowCal package (v1.3.0) were used to collect and analyze cytometry data. Custom scripts available upon request from the corresponding author.
Cell population abundance	Sorting of cells was performed on the SH800 Cell Sorter using the Semi-Purity setting, which increases purity of cells sorted to ~97% from ~95% in Normal mode.
Gating strategy	For flow cytometry experiments, a "high_low" gate was set on the FSC/SSC plots using the FlowCal package, where only cells contained within 10^4 and 8x10^5 arb for both axes were kept. These values were determined based on the sample set, where the full distribution of cells was captured without consideration for clear outliers.<br>For sorting experiments, we set an ellipse gate on FSC/SSC plots to capture the full distribution of our population. We then set a sorting gate to capture and sort cells in the top 14% of this population (for the first round of FACS, uninduced library) and the bottom 85% of the population (for the 2nd round of FACS, induced library).

- [x] Tick this box to confirm that a figure exemplifying the gating strategy is provided in the Supplementary Information.
